# CDKL Family Kinases Have Evolved Distinct Structural Features and Ciliary Function

**DOI:** 10.1016/j.celrep.2017.12.083

**Published:** 2018-01-28

**Authors:** Peter Canning, Kwangjin Park, João Gonçalves, Chunmei Li, Conor J. Howard, Timothy D. Sharpe, Liam J. Holt, Laurence Pelletier, Alex N. Bullock, Michel R. Leroux

**Affiliations:** 1 Structural Genomics Consortium, University of Oxford, Old Road Campus, Roosevelt Drive, Oxford OX3 7DQ, UK; 2 Department of Molecular Biology and Biochemistry, and Centre for Cell Biology, Development, and Disease, Simon Fraser University, 8888 University Drive, Burnaby, BC V5A 1S6, Canada; 3 Lunenfeld-Tanenbaum Research Institute, Mount Sinai Hospital, 600 University Avenue, Toronto, ON M5G 1X5, Canada; 4 Department of Molecular Genetics, University of Toronto, Toronto, ON M5S 1A8, Canada; 5 Department of Molecular & Cell Biology, University of California, Berkeley, Berkeley, CA 94720, USA

**Keywords:** cilium length, Cyclin-Dependent Kinase-Like, CDKL, kinase, neurological disorder, protein structure

## Abstract

Various kinases, including a cyclin-dependent kinase (CDK) family member, regulate the growth and functions of primary cilia, which perform essential roles in signaling and development. Neurological disorders linked to CDK-Like (CDKL) proteins suggest that these underexplored kinases may have similar functions. Here, we present the crystal structures of human CDKL1, CDKL2, CDKL3, and CDKL5, revealing their evolutionary divergence from CDK and mitogen-activated protein kinases (MAPKs), including an unusual αJ helix important for CDKL2 and CDKL3 activity. *C. elegans* CDKL-1, most closely related to CDKL1–4 and localized to neuronal cilia transition zones, modulates cilium length; this depends on its kinase activity and αJ helix-containing C terminus. Human CDKL5, linked to Rett syndrome, also localizes to cilia, and it impairs ciliogenesis when overexpressed. CDKL5 patient mutations modeled in CDKL-1 cause localization and/or cilium length defects. Together, our studies establish a disease model system suggesting cilium length defects as a pathomechanism for neurological disorders, including epilepsy.

## Highlights

• CDKL2 and CDKL3 structures have distinct C-terminal αJ helix important for activity
• Human CDKL5 and *C. elegans* CDKL-1 localize to and have roles in cilia
• CDKL-1 cilia length control depends on kinase activity and C terminus with αJ helix
• CDKL5 disease-linked mutations cause defects in CDKL-1 cilia length regulation

## Graphical Abstract

**Figure:**
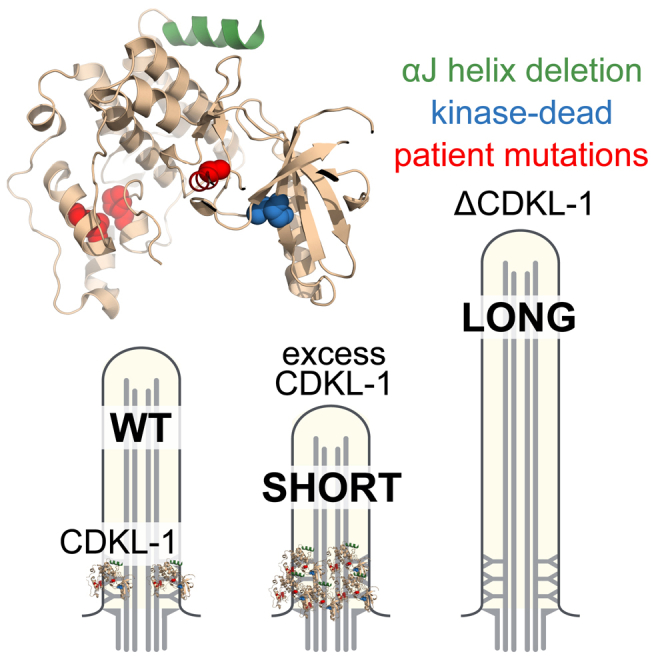

## Teaser

Canning et al. reveal distinct structural features of CDKL kinases and a role for *C. elegans* CDKL-1 in cilium length control, which is lost in a kinase-dead mutant and by introducing CDKL5 disease-linked mutations. The study suggests ciliary length impairment as a potential mechanism that contributes to human neurological disorders.

## Introduction

Primary (non-motile) cilia are organelles found in most eukaryotic cells, including neurons, that perform essential roles in human sensory physiology, cell signaling, and development (May-Simera and Kelley, 2012, Mukhopadhyay and Rohatgi, 2014, Oh and Katsanis, 2012). They are anchored by a basal body that templates the growth of the microtubule-based axoneme (Carvalho-Santos et al., 2011). The first ciliary subcompartment formed is the transition zone (TZ), which harbors Y-shaped links that make axoneme-to-membrane connections. The TZ functions as a membrane diffusion barrier that maintains the correct ciliary composition of the compartment, which is enriched in signaling proteins (Reiter et al., 2012, Williams et al., 2011). The axoneme is built by an intraflagellar transport (IFT) system that uses kinesin/dynein motors and adaptors that mobilize cargo into and out of cilia (Blacque and Sanders, 2014, Sung and Leroux, 2013). Disruption of basal body, TZ, and IFT proteins results in ciliopathies that exhibit a broad spectrum of clinical ailments (Reiter and Leroux, 2017).

The length of cilia must be tightly regulated to ensure optimal functions in their given cell types (Keeling et al., 2016). IFT plays a central role in cilium length control (Broekhuis et al., 2013). Length regulation further involves depolymerizing kinesins and tubulin modifications that influence microtubule stability (Liang et al., 2016). Kinases represent another category of proteins that influence cilium formation as well as length (Avasthi and Marshall, 2012, Cao et al., 2009).

**Figure 1:**
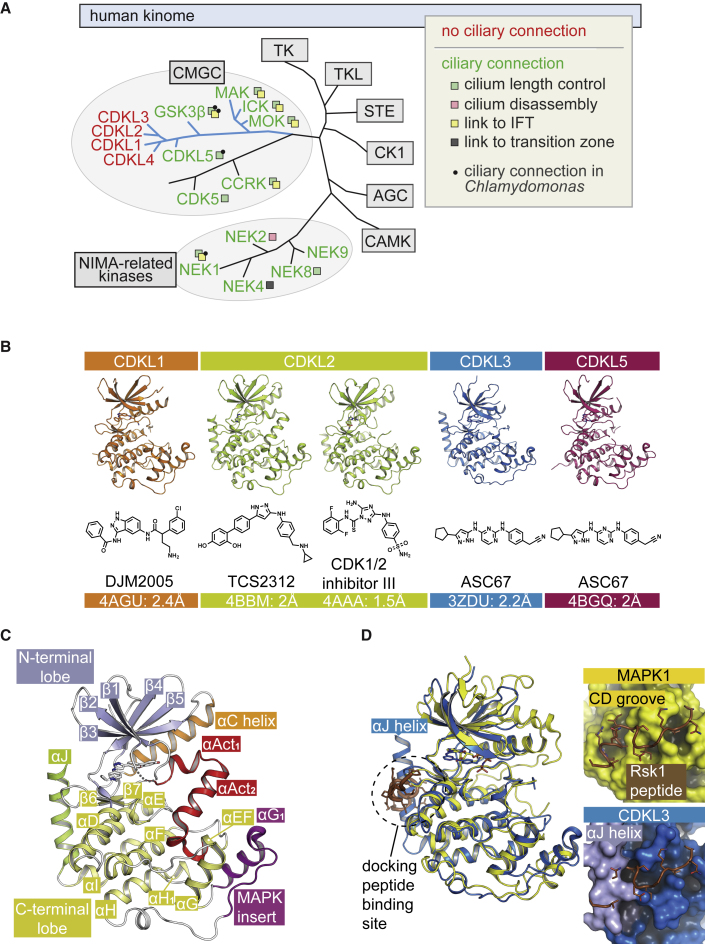
Unusual Structural Features of the CDKL Kinase Domains (A) Phylogenetic distribution of NIMA-related kinases (Neks) or CMGC group kinases with known ciliary functions (green), including cilium length control, disassembly, association with intraflagellar transport (IFT), and TZ localization. A branch of the CMGC group (blue) includes several kinases (mammalian MAK, ICK, and MOK; *Chlamydomonas* GSK3β and CDKL5) that regulate cilium length, several of which also influence IFT. Human CDKL1, CDKL2, CDKL3, and CDKL4 (red) have no previously known links to cilia. (B) Crystal structures of human CDKL1, CDKL2, CDKL3, and CDKL5 with the indicated inhibitors. (C) Structural features of the CDKL2-TCS2312 complex, the most complete/ordered CDKL structure. (D) Structural comparison of CDKL3 with MAPK1 (ERK2; PDB ID: 3TEI). Inset panels show Rsk1 docking peptide bound to MAPK1 and superimposed onto CDKL3.

Kinases implicated in cilium length control largely belong to two groups. One is the NIMA-related kinase (Nek) family, which includes mammalian Nek1 and Nek8, and CNK2, CNK4, and CNK11 from *Chlamydomonas* (Bradley and Quarmby, 2005, Hilton et al., 2013, Lin et al., 2015, Meng and Pan, 2016, Shalom et al., 2008, Sohara et al., 2008). CMGC kinases (CDKs, mitogen-activated protein kinases [MAPK], glycogen synthase kinases [GSK], and CDK-like kinases [CLK]) represent the other group, with mammalian Cyclin-Dependent Kinase 5 (CDK5) and Cell Cycle-Related Kinase (CCRK) influencing cilium length (Husson et al., 2016, Phirke et al., 2011, Tam et al., 2007, Yang et al., 2013). Several members from a specific branch of CMGC kinases (Figure 1A) also regulate cilium length, namely ICK, MAK, MOK, GSK3β, and CDK-Like 5 (CDKL5) (Bengs et al., 2005, Berman et al., 2003, Broekhuis et al., 2014, Burghoorn et al., 2007, Hu et al., 2015, Omori et al., 2010, Tam et al., 2013, Wilson and Lefebvre, 2004). Notably, human CDKL5 belongs to a family of CDKL kinases encompassing CDKL1, CDKL2, CDKL3, and CDKL4.

CDKL proteins share a high degree of sequence similarity with CDKs, and they contain the MAPK TXY phosphorylation motif needed for activity (Yee et al., 2003). They have putative cyclin-binding domains, but there is no evidence of interaction with cyclins. However, no CDKL family member has been structurally characterized. Moreover, aside from CDKL5, little is known about CDKL protein function. Disrupting CDKL5 causes Rett syndrome, a neurodevelopmental disorder that exhibits early-onset seizures, mental retardation, and autism (Castrén et al., 2011, Kilstrup-Nielsen et al., 2012). Consistent with having neuronal functions, CDKL5 facilitates dendritic spine and excitatory synapse formation, possibly via AKT/GSK-3β signaling (Fuchs et al., 2014). Intriguingly, *Chlamydomonas* CDKL5 orthologs regulate cilium length (Hu et al., 2015, Tam et al., 2013); however, such a function in metazoans has not been reported. Knockdown of zebrafish CDKL1 causes Hedgehog signaling defects (Hsu et al., 2011) that hint at a ciliary role, but the localization and function of the protein remain unknown.

Here we present the crystal structures of CDKL1, CDKL2, CDKL3, and CDKL5, solved in various active and inactive kinase domain conformations. The structures reveal an unusual αJ helix important for CDKL2 and CDKL3 function and further structural changes to putative substrate docking sites that support the divergence of CDKL kinases from the CDK and MAPK families. We show that, unlike other TZ proteins, the sole *C. elegans* CDKL protein family member (CDKL-1), which localizes to the ciliary TZ (Li et al., 2016), does not regulate the diffusion barrier. Instead, CDKL-1 regulates cilium length, in a kinase activity- and αJ helix C-terminal region-dependent manner. We present evidence that human CDKL5 is a ciliary protein with a potential role in ciliogenesis, and we show that *C. elegans* CDKL-1 variants modeling CDKL5 human patient mutations exhibit cilium length defects, with or without loss of TZ localization. Together, our structure-function studies provide the first high-resolution structural insights into the CDKL protein family; reveal that CDKL proteins may share a common function in cilium length control; and show that CDKL5-associated Rett syndrome may stem, at least in part, from ciliary dysfunction.

## Results and Discussion

### The CDKL Kinase Domain Contains an Unusual αJ Helix

CDKL family proteins contain a conserved N-terminal kinase domain and variable C termini (Figure S1). The kinase domains of CDKL1, CDKL2, CDKL3, and CDKL5 were crystallized using a phosphomimetic Asp-X-Glu (DXE) substitution and in the presence of identified inhibitors (Table S1). Their structures were solved at resolutions from 1.5 to 2.4 Å (Figure 1B; Table S2). The prototypical structure, exemplified by CDKL2, conformed to the classic bilobal kinase architecture (Figure 1C). Of note, the C-terminal lobe diverged at the two MAPK family docking sites, suggesting that CDKL family members mediate alternative protein interactions. First, the MAPK insert folded into a single large αG_1_ helix and loop, whereas MAPKs typically contain two shorter helices for recruiting the substrate Asp-Glu-Phe (DEF) motif (Figure 1D). The typical MAPK insert also packed against the αG helix, orientated roughly perpendicular to the equivalent motif in CDKL2 (Figure 1D). Second, the CDKL2 kinase domain was extended at the C terminus by an unusual αJ helix that occupied a site equivalent to the MAPK common docking (CD) groove and, thus, occluded part of the recruitment site for the D(ocking) motifs of MAPK substrates (Figure 1D). Interestingly, the packing and orientation of the αJ helix was distinct from the C-terminal extensions of other kinases, such as PAK1, CDK2, BUB1, and NEK1 (Figure S2). Hence, it will be interesting to explore the proteomics of these kinases, with or without the αJ helix region, to uncover any specific protein interactions. Other divergent docking sites, such as the MAPK insert, further indicate the status of CDKL proteins as a distinct kinase family, for which key regulatory and substrate partners remain to be identified.

### Small-Molecule Inhibitors Can Bind to Both Active and Inactive CDKL Conformations

**Figure 2:**
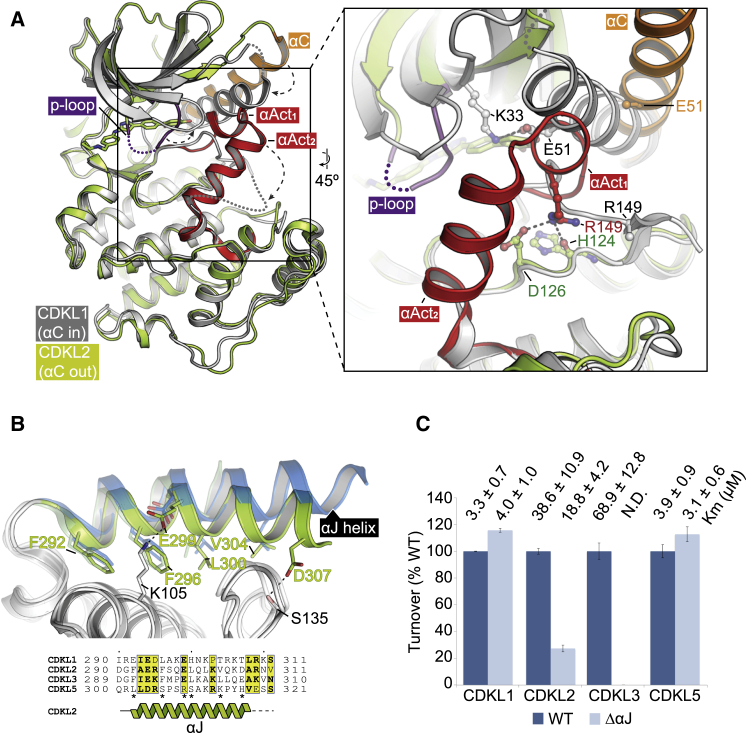
Structural Features Determining CDKL Activation (A) Superposition of CDKL1 (αC-in, gray) and CDKL2 (αC-out, light green). Inset highlights changes in the positions of the αC and activation segments. (B) Sequence and structural comparisons show the αJ conservation in CDKL2 (green) and CDKL3 (blue). An asterisk denotes αJ interactions with the kinase domain. (C) A radiometric *in vitro* kinase assay reveals that the αJ region is critical for CDKL2 and CDKL3 activities but dispensable for CDKL1 and CDKL5. K_m_ values are shown for the Ime2 peptide substrate. N.D. denotes not determined for CDKL3(ΔαJ) due to diminished catalytic activity.

The CDKL structures all contain broad spectrum ATP-competitive inhibitors that bind to the kinase hinge region via 3 hydrogen bonds (Figures S3A–S3C). CDKL2 co-crystallized in an inactive αC-out conformation with two inhibitors, CDK1/2 Inhibitor III and CHK1 inhibitor TCS2312 (Figure 2A). Their binding was stabilized by a collapsed P loop conformation that establishes potential allosteric sites for inhibitor design (Figures S3A and S3B). While an inactive conformation appears necessary for TCS2312 binding, the CDK1/2 Inhibitor III is compatible with an active kinase conformation, suggesting that the inactive configuration of CDKL2 is not driven solely by inhibitor interactions.

By contrast, the CDKL1, CDKL3, and CDKL5 structures displayed characteristics of active kinases. Inhibitor DJM2005 showed a preference for CDKL1, and it formed an extra H-bond to the catalytic loop residue N131 (Figure S3C). Inhibitor ASC67 was designed as an affinity reagent (Statsuk et al., 2008) and was among the top screening hits for all CDKLs tested (Table S1). Its nitrile H bonded with the catalytic lysines of CDKL3 and CDKL5 (Figure S3C). Catalytic site interactions may help support the active conformation of these kinases. Together, the structures and inhibitor screens suggest potential for generating isoform- as well as conformation-selective inhibitors of the CDKL family.

### CDKL Structures Reveal Conformational Changes during Kinase Activation

Superposition of CDKL1 and CDKL2 highlighted conformational changes needed for kinase activation (Figure 2A). In CDKL2, the ATP-binding pocket was sterically occluded by the P loop and activation segment (αAct_1_ and αAct_2_). This forced the αC helix to swing outward, breaking the salt bridge between the catalytic lysine (K33) and glutamate (E51, αC). The CDKL1 structure showed an ∼11-Å shift in αC position that restored the salt bridge (Figure 2A). In this active configuration, the P loop also formed the expected β1-β2 hairpin. However, the substrate-binding pocket was disrupted by a disordered activation segment, showing incomplete activation by the phosphomimetic DXE motif (Figure 2A).

### The αJ Helix Is Critical for the Kinase Activity of CDKL2 and CDKL3

To date, no activating partners are known for the CDKL family. Structural comparisons with the αC PSTAIRE motif of CDK2 reveal several bulky substitutions in CDKLs that likely preclude binding to cyclins, although novel interaction partners cannot be excluded (Figure S3D). MAPKs establish a similar αC interaction intramolecularly through their C-terminal helix αL16. However, we found no evidence for an equivalent structural element in CDKLs. Instead, CDKL2 and CDKL3 showed an unusual amphipathic helix, αJ, while the constructs for CDKL1 and CDKL5 were truncated and lacked this region (Figure 2B).

To determine the functional relevance of the αJ, we expressed the wild-type (WT) proteins in yeast, and we performed *in vitro* kinase assays (Figure 2C). Proline-directed activity was observed against an Ime2 peptide substrate (RPRSPGARR), consistent with other CMGC kinases. Turnover was low (<10 phosphorylations/min) for all CDKLs, perhaps reflecting a requirement for activating partners. Notably, deleting the αJ region reduced the activities of CDKL2 and CDKL3, whereas CDKL1 and CDKL5 were largely unchanged (Figure 2C). These results are consistent with the observed structures, and they further show the importance of the unprecedented αJ helix for CDKL2 and CDKL3 function.

### *C. elegans* CDKL-1 Localizes to the Ciliary Transition Zone but Appears Dispensable for Cilium Gate Function

**Figure 3:**
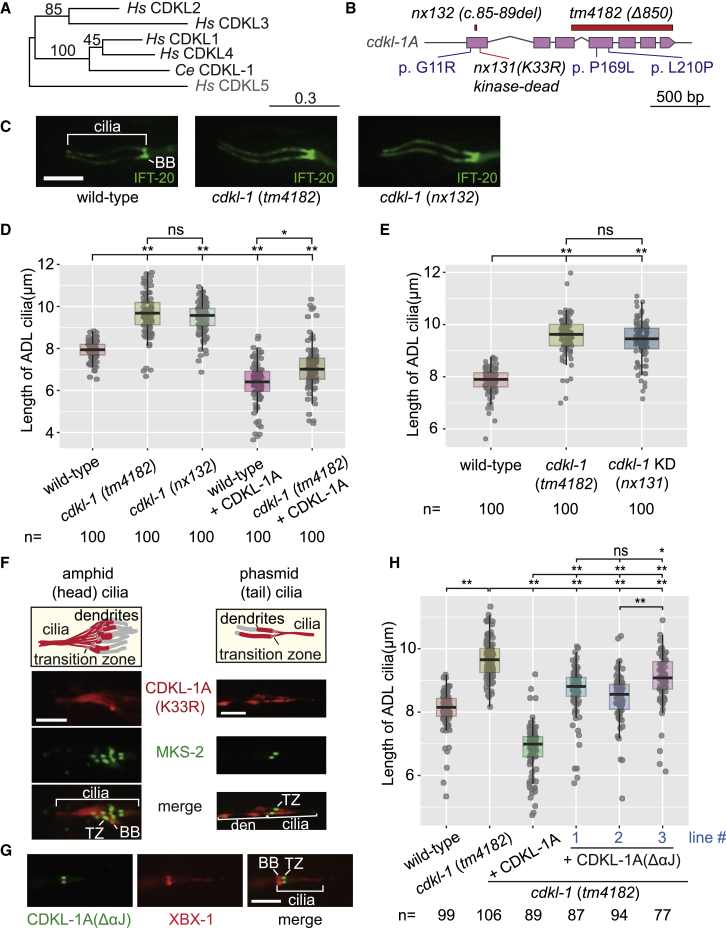
*C. elegans* CDKL-1 Requires Its Kinase Activity and C-Terminal Region (Including αJ Helix) to Regulate Cilium Length (A) Phylogenetic relationship between *H. sapiens* (*Hs*) and *C. elegans* (*Ce*) CDKL proteins. (B) Gene structure of *cdkl-1A*, highlighting the deletion or missense mutants analyzed. (C) Representative images of the GFP-tagged IFT-20 marker expressed specifically in ADL neurons (L4 larvae), used to measure the length of cilia in WT and *cdkl-1* mutants (*tm4182* and *nx132*). ADL doublet cilia are longer in mutants than WT. BB, basal body. Scale bar, 4 μm. (D) ADL cilia lengths (L4 larvae) of WT and *cdkl-1* mutants with/without expression of WT CDKL-1A construct. Each dot represents one cilium. Kruskal-Wallis test (Dunn Kruskal-Wallis multiple comparison [Holm-Sidak method]) was used for significance in (D), (E), and (H). ^∗^p < 0.01 and ^∗∗^p < 0.001; ns, not significant. (E) ADL cilia length in WT, *cdkl-1* null (*tm4182*), and *cdkl-1* kinase-dead (KD) (*nx131*) mutant L4 larvae. Dot, one cilium. ^∗∗^p < 0.001; ns, not significant. (F) The kinase-dead variant CDKL-1A(K33R)::tdTomato no longer concentrates at the TZ (marked by MKS-2::GFP); it mislocalizes to dendrites (den) and cilia in amphid and phasmid neurons. BB, basal body. Scale bar, 4 μm. (G) CDKL-1A(ΔαJ)::mNeonGreen protein predominantly accumulates at the TZ in cilia. The BB and ciliary axoneme are marked by XBX-1::tdTomato. Scale bar, 4 μm. (H) ADL cilia lengths (L4 larvae) measured in WT, *cdkl-1* null (*tm4182*), and *cdkl-1* null (*tm4182*) expressing CDKL-1A or CDKL-1A(ΔαJ). Dot, one cilium. ^∗^p < 0.05 and ^∗∗^p < 0.001; ns, not significant.

To investigate the collective function of CDKL proteins, we chose to study the sole member encoded by *C. elegans*, CDKL-1. This kinase is most closely related to mammalian CDKL1–4 and more distantly related to CDKL5 (Figure 3A). The *cdkl-1* gene is specifically expressed in ciliated sensory neurons (Figure S4A), likely due to the presence of an X-box motif found in the promoters of most ciliary genes (Blacque et al., 2005). The CDKL-1 protein localizes to ciliary TZs in head (amphid) and tail (phasmid) neurons (Li et al., 2016) (Figure S4B), whose ascribed functions are as a membrane diffusion barrier, or ciliary gate, that maintains the protein composition of the organelle (Reiter et al., 2012).

Given its TZ localization, we wondered if CDKL-1 plays a role in ciliary gating. To test this, we probed if two proteins normally found at the periciliary membrane, namely TRAM-1a and RPI-2, could inappropriately enter the ciliary compartment in a strain lacking CDKL-1. TRAM-1a and RPI-2 entered cilia in most TZ mutants tested (Huang et al., 2011, Jensen et al., 2015, Williams et al., 2011). In contrast, they remained at the periciliary membrane in the *cdkl-1* mutant, as in the WT control (Figures S4C and S4D). We also queried for the leakage of ARL-13 from its normal localization (ciliary middle segment) to the periciliary membrane, as seen in various TZ mutants (Cevik et al., 2013, Li et al., 2016). ARL-13 ciliary localization was unchanged in the *cdkl-1* mutant, similar to WT (Figure S4E).

Although the *cdkl-1*(*tm4182*) mutant used above contains a large out-of-frame deletion (850 bp) of exons 3–7 and is likely null, we also made another *null* (*nx132*) mutant using CRISPR-Cas9, which has a 5-bp deletion in the first coding exon, causing an early stop (Figure 3B). TRAM-1a, RPI-2, and ARL-13 localization remained unperturbed in this *cdkl-1* mutant (Figures S4C–S4E), providing further evidence that CDKL-1 performs a non-canonical function unrelated to ciliary gating at the TZ.

### CDKL-1 Modulates Cilium Length

Given that MAK, ICK, MOK, GSK3β, and CDKL5 regulate cilium length (Figure 1A), we hypothesized that *C. elegans* CDKL-1 plays a similar role. To test this, we expressed GFP-tagged IFT-20 in the bi-ciliated ADL neuron to measure cilium length accurately (Mohan et al., 2013). IFT-20::GFP, which marks the basal body and axoneme, was introduced into WT and *cdkl-1* mutant (*tm4182* and *nx132*) animals. Whereas the median length of WT ADL cilia was 8.0 μm, *cdkl-1* mutant cilia were 9.6 μm, or ∼20% longer (Figures 3C and 3D). We confirmed that the long ADL cilia correctly penetrated amphid channels, which are formed by sheath and socket cells (Figure S4F).

We sought to rescue the cilium length defect of *cdkl-1* mutants by expressing WT *cdkl-1*, but we found that this shortens ciliary length by ∼11%, to 7.1 μm (Figures 3D and S4G). Similarly, overexpressing *cdkl-1* in a WT background reduced ciliary length. Hence, loss or increased levels of CDKL-1 activity led to longer or shorter cilia, suggesting that the correct level of CDKL-1 kinase activity is needed to maintain correct cilium length.

Phylogenetically, CDKL proteins are split into two ancestral groups, namely CDKL1–4 and CDKL5. Our results provide the first evidence for a CDKL1–4-related protein in cilium length regulation. As *C. elegans* only encodes one CDKL protein, related to CDKL1–4 (as in *Drosophila*), our results suggest that both CDKL5 and CDKL1–4 proteins may share functions in cilium length regulation. Vertebrates/mammals encode CDKL5 and CDKL1/2/3/4 proteins. This may indicate divergent functions for the different members, which may be cilium dependent and/or independent, including cell proliferation and tumorigenesis, neuronal differentiation, cognition, and learning (Gomi et al., 2010, Kilstrup-Nielsen et al., 2012, Liu et al., 2010, Sun et al., 2012).

### CDKL-1 Kinase Activity Is Required for Cilium Length Control and Transition Zone Localization

We examined if CDKL-1 kinase activity is needed to regulate cilium length. Using CRISPR-Cas9, we generated a *cdkl-1* kinase-dead mutant (*nx131*) by converting a conserved lysine (K) in the ATP-binding site to arginine (R) (Figures 3B and S5D). Like the *cdkl-1* (*tm4182*) null mutant, the kinase-dead mutant exhibited cilia ∼20% longer than WT (Figure 3E). In addition, the CDKL-1(K33R) protein no longer concentrated at the TZ; it dispersed in the dendrite and ciliary axoneme (Figure 3F). Thus, CDKL-1 kinase activity is critical for regulating cilium length and proper TZ localization of the protein. Whether CDKL-1 phosphorylates itself and/or target(s) within the TZ, which promotes TZ localization, is unclear. Future studies aimed at uncovering targets (perhaps IFT proteins), and interaction partners, will shed light on the mechanism by which CDKL-1 regulates cilium length.

### C-terminal αJ Helix Region of CDKL-1 Is Needed to Maintain Ciliary Length

Since our structure-function studies uncovered the αJ helix as crucial for CDKL2/CDKL3 kinase activity, we probed its role in *C. elegans*. An mNeonGreen-tagged CDKL-1A(ΔαJ) protein variant was expressed, and it was found to localize correctly to the TZ, suggesting that this region does not overtly affect protein stability (Figure 3G). However, unlike WT CDKL-1A, the CDKL-1A(ΔαJ) protein only partially rescued the cilium length defect in a *cdkl-1* mutant, suggesting that the αJ helix region is important for cilium length control (Figures 3H and S4H).

### CDKL-1 Variants Carrying Human CDKL5 Pathogenic Mutations Disrupt Ciliary Length Control

**Figure 4:**
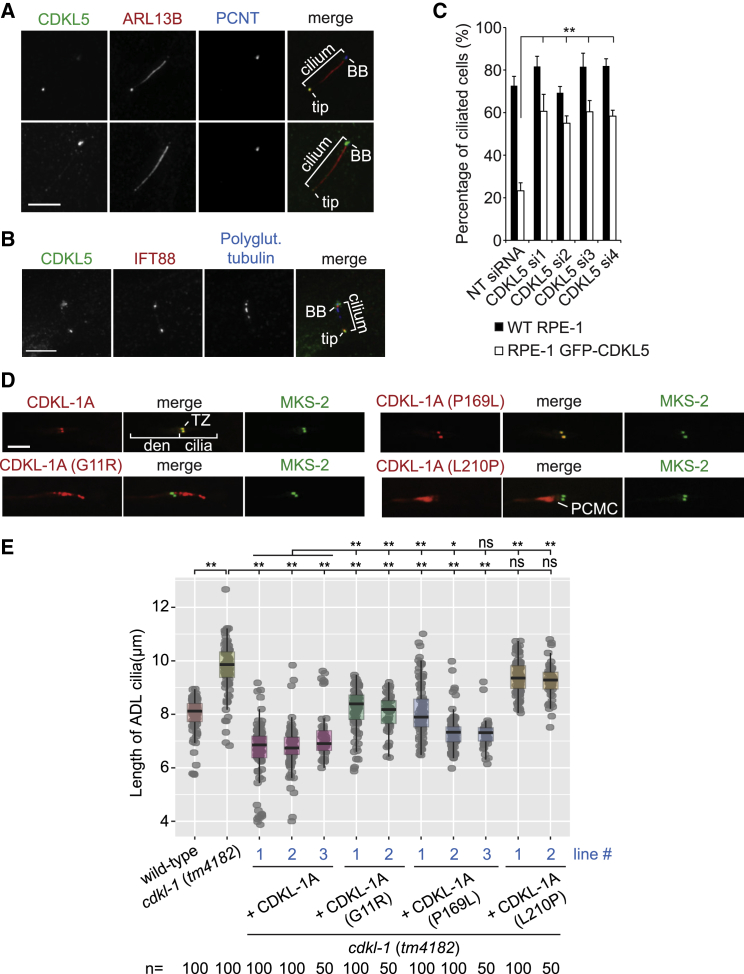
Human CDKL5 Localizes to Cilium and Affects Ciliogenesis When Overexpressed in RPE-1 Cells and Mutations Modeled in *C. elegans* CDKL-1 Disrupt Localization and/or Cilium Length Regulation (A) Immunofluorescence analysis of serum-starved hTERT RPE-1 cells expressing GFP-CDKL5 stained with antibodies against GFP, ARL13B (cilium marker), and PCNT (centrosome marker). The top and bottom panels are representative images showing different levels of GFP-CDKL5 localization to the BB and cilium tip. Scale bar, 5 μm. (B) Serum-starved hTERT RPE-1 GFP-CDKL5 cells were stained with antibodies against GFP, IFT88, and polyglutamylated tubulin (centriole/BB and axoneme marker). Scale bar, 5 μm. (C) Bar graph shows mean percentage of ciliated cells (n > 300 cells per sample, 3 independent experiments) in the serum-starved populations (72 hr) of hTERT RPE-1 and hTERT RPE-1 GFP-CDKL5 cells transfected with the indicated siRNAs. Error bars indicate SD. ^∗∗^p < 0.01 (Student’s two-tailed t test). (D) WT CDKL-1A and the P169L variants localize to the TZ, whereas the G11R mutant localizes along the length of cilia, and the L210P mutant accumulates in dendrites (den) and is weakly present at the periciliary membrane compartment (PCMC). MKS-2 is a TZ marker. Scale bar, 4 μm. (E) ADL cilia lengths (L4 larvae) of WT and strains expressing CDKL-1 variants. Expressing WT CDKL-1A leads to short cilia. Similar levels of length reduction are not seen upon expression of the G11R and L210P variants, or lines 1 and 2 of the P169L variant (line 3 is not significant), suggesting functional defects with these variants. Dot is one cilium. Significance (p value) was calculated by Dunn Kruskal-Wallis multiple comparison (Holm-Sidak adjustment). ^∗^p < 0.01 and ^∗∗^p < 0.001; ns, not significant.

CDKL5 mutations, most occurring in the kinase domain, are linked to neurological disorders, including epilepsy, atypical Rett syndrome, and autism. We sought to model such CDKL5 mutations using *C. elegans* CDKL-1, but first we wanted to reveal a functional connection between human CDKL5 and cilia to ensure the relevance of our studies. To this end, we found that GFP-tagged human CDKL5 localizes at the basal body, as well as ciliary tip in ciliated RPE-1 cells (Figures 4A and 4B). In non-ciliated cells, GFP-CDKL5 localized to the centrosome in a microtubule-independent manner (Figure S5A). Compared to serum-starved WT RPE-1 cells, cells expressing GFP-CDKL5 exhibited compromised ciliogenesis (Figures 4C and S5C). This negative effect of GFP-CDKL5 overexpression was rescued by RNAi-mediated depletion of CDKL5 (Figures 4C, S5B, and S5C). Our findings confirm the first association between vertebrate/mammalian CDKL5 and cilia.

We chose to model three pathogenic CDKL5 missense mutations (G20R, P180L, and L220P) present in patients with epileptic encephalopathy, severe mental retardation, developmental delay, or spasms (Kilstrup-Nielsen et al., 2012). The residues are conserved in all human CDKL proteins and *C. elegans* CDKL-1 (Figures S1D and S5D). The corresponding mutations (G11R, P169L, and L210P) were introduced in *C. elegans* CDKL-1 to model their influence on TZ localization (Figure 3B). Strikingly, CDKL-1 proteins harboring the G11R or L210P mutations no longer concentrated at the TZ (Figure 4D). CDKL-1(G11R) was primarily dispersed in the ciliary axoneme, whereas CDKL-1(L210P) showed weak localization to cilia and periciliary membrane. In contrast, CDKL-1(P169L) localized to the TZ, similar to WT.

Next, we assessed the functionality of each CDKL-1 variant by testing their effect on ADL cilium length when expressed in a *cdkl-1* mutant. Relative to WT CDKL-1, expression of each variant gave statistically longer cilia in almost all lines, with the phenotypic severity ranked L210P > G11R > P169L (Figure 4E). Substituting proline in the L210P variant likely disrupted the αG helix, potentially causing protein misfolding (Figure S5E) and, thus, dysfunction due to mislocalization and/or loss of kinase activity. The G11R missense mutation was positioned at the start of the P loop GXGXXG motif, where a bulky substitution was predicted to preclude ATP binding (Figure S5E). This may have reduced CDKL-1 catalytic activity and, similar to the kinase-dead mutant (Figure 3F), induced mislocalization. The weaker P169L phenotype may have reflected a partial loss of function (2 of 3 lines showed different ciliary lengths), with the protein remaining TZ localized (Figures 4D and 4E).

Together, our modeling of CDKL5 patient mutations using *C. elegans* CDKL-1 suggests that cilium length may, at least in part, cause the observed neurological phenotypes present in patients with CDKL5 mutations. Dissecting the relationship among kinase activity, TZ localization, and mechanism of ciliary length regulation will necessitate further studies.

### Model for CDKL Protein Regulation of Cilium Length

**Figure 5:**
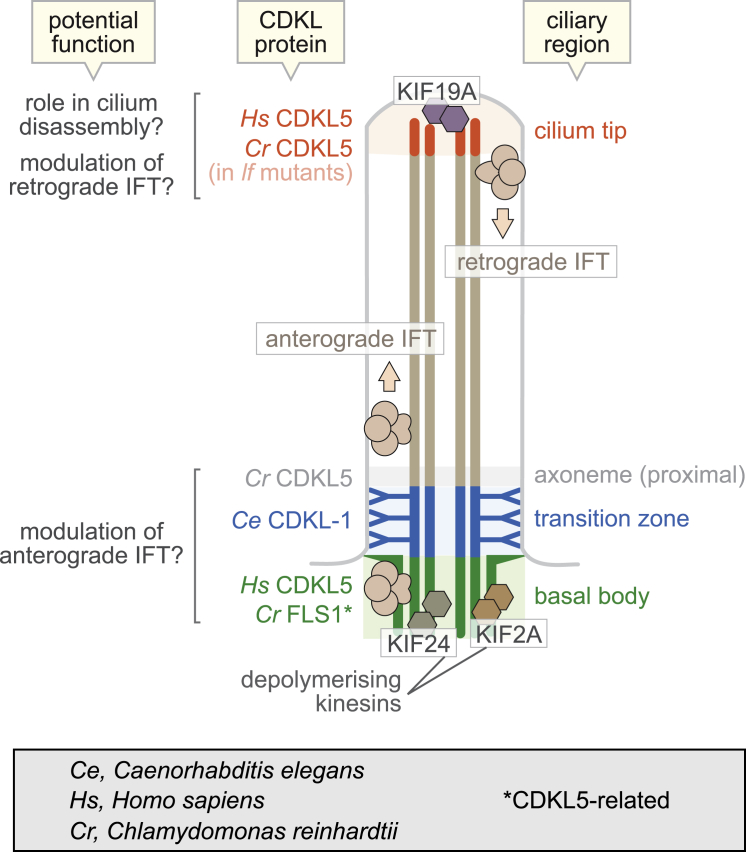
Model for CDKL Protein Ciliary Localization and Potential Roles in Cilium Length Regulation CDKL proteins localize to the base and/or tip of cilia, and they likely influence IFT (anterograde/retrograde) machinery and/or depolymerizing kinesins to regulate cilium growth and disassembly, respectively, and thus control cilium length.

One specific branch of the kinome (Figure 1A) is enriched with proteins having ciliary functions (Avasthi and Marshall, 2012). ICK, MOK, and MAK localize to cilia and negatively regulate their length (Broekhuis et al., 2014, Chaya et al., 2014, Omori et al., 2010). Notably, IFT components accumulate inside cilia when ICK or MAK are lost. Moreover, ICK phosphorylates a subunit of kinesin-2 and affects IFT speeds, consistent with the link between cilium length control and IFT. *Chlamydomonas* orthologs of CDKL5 and GSK3β (LF5/FLS1 and GSK3) also localize to, and control the length of, cilia (Hu et al., 2015, Tam et al., 2013, Wilson and Lefebvre, 2004). *Chlamydomonas* CDKL5 (LF5) localizes at the ciliary base and its disruption lengthens cilia (Tam et al., 2013), similar to loss of *C. elegans* CDKL-1. Interestingly, *Chlamydomonas* CDKL5 moves to ciliary tips in long-flagella (*lf*) mutants, suggesting a link to length control. Specifically, CDKL5 is influenced by LF3, whose loss causes IFT protein accumulation at ciliary tips (Tam et al., 2003). This is of interest, since human CDKL5 localizes at the base, and also tip, of cilia (Figures 4A, 4B, and 5). Hence, CDKL5 (and perhaps other CDKL proteins) influence IFT at the base, or tip, of cilia to regulate cilium length. Consistent with such a role, overexpressing CDKL5 impairs cilium formation in RPE-1 cells.

Interestingly, *C. elegans* CDKL-1 localizes to the TZ, whereas human CDKL5 and another CDKL5-related *Chlamydomonas* protein (FLS1) are at the basal body (Hu et al., 2015) (Figure 5). This might reflect different functions of CDKL1–4 or CDKL5 family members, although the *Chlamydomonas* CDKL5 is present just distal to the TZ. Hence, CDKL proteins exist in three regions at the ciliary base—basal body, TZ, and distal to the TZ (Figure 5). At any of these locations, CDKL proteins may be well positioned to interact with the IFT machinery. Such a transient interaction has been described for the TZ protein B9D1 (Zhao and Malicki, 2011). Another possibility, not mutually exclusive, is that CDKL5 and perhaps other CDKL proteins act via depolymerizing kinesin(s). This is reported for *Chlamydomonas* FLS1, which phosphorylates a kinesin-13 member in flagella (Hu et al., 2015). In mammals, two cilium base-localized kinesin-13 proteins, KIF2A and KIF24, regulate cilium disassembly/length control (Kim et al., 2015, Kobayashi et al., 2011, Miyamoto et al., 2015) (Figure 5). CDKL proteins may also influence another depolymerizing kinesin, KIF19A, found at the ciliary tip (Niwa et al., 2012). In sum, CDKL proteins may control cilium structure and length through the IFT machinery and/or depolymerizing kinesins, at the ciliary base and/or tip (Figure 5). Understanding their ciliary roles may be relevant to human neurological disorders, including epilepsy.

## Experimental Procedures

### Cloning of Human CDKL Kinase Domains

The CDKL1 kinase domain sequence (UniProt: Q00532; residues 1–303; T159D, Y161E, and N301D) was cloned into the bacterial vector pNIC-CTHF. The CDKL2 (UniProt: Q92772; residues 1–308; T159D and Y161E), CDKL3 (UniProt: Q8IVW4; residues 1–324; T158D and Y160E), and CDKL5 (UniProt: O76039; residues 1–303; T169D and Y171E) domains were cloned into the baculoviral vector pFB-LIC-Bse. More details are in the Supplemental Experimental Procedures.

### *In Vitro* Kinase Assays

Kinase activity was profiled in 50 mM HEPES (pH 7.5), 50 mM NaCl, 10 mM MgCl_2_, 500 μM ATP, 83.3 μg/mL BSA, 0.833% glycerol, and 0.2 μCi [γ-^32^P]ATP. Minimal kinase concentrations sufficient for signal (0.05–10 μM) were determined empirically. See also the Supplemental Experimental Procedures.

### Construction of *C. elegans* cdkl-1A Translational Fusions

For the various *cdkl-1A* (Y42A5A.4A) translational fusions, all introns and exons of *cdkl-1A*, including its 1.8-kb 5′ UTR, were amplified from the genomic DNA, and they were fused to GFP or tdTomato with the *unc-54* 3′ UTR or *cdkl-1* 3′ UTR without any fluorescent proteins tags. The mutations found in CDKL5 human patients (G20R, P180L, and L220P) or kinase-dead (CDKL5 K42R) mutations were introduced into the *cdkl-1* gene by replacing the corresponding residues, and they were fused to tdTomato to assess localization. Plasmids encoding tdTomato-tagged or untagged CDKL-1A variants harboring CDKL5 mutations, and those encoding CDKL-1A(ΔαJ) (residues 1–286) with/without mNeonGreen, were prepared with the CloneJET PCR Cloning Kit.

### *C. elegans* Strains and Imaging

All nematode strains (Table S3) were maintained at 20°C and imaged using a spinning-disc confocal microscope, as described in Li et al. (2016).

### ADL Ciliary Length Measurement and Statistical Analysis

ADL cilia lengths (from basal body to tip) of L4 larvae were measured and plotted using Dot and Boxplots in R software. The distribution of each dataset was determined by Shapiro-Wilk test. The statistical significance (p value) was calculated by the Dunn’s Kruskal-Wallis Multiple Comparisons with Holm-Sidak adjustment.

### Human CDKL5 Constructs

A Gateway entry clone with the coding sequence of human CDKL5 (NM_003159.2) was obtained from the LTRI plasmid repository, and it was used to clone CDKL5 in fusion with GFP in the pcDNA5-FRT/TO-GFP vector. The GFP-CDKL5 fusion was subsequently subcloned into the lentiviral vector pHR-SIN-SFFV to generate the pHR-SIN-SFFV-GFP-CDKL5 plasmid.

### Mammalian RNAi and Statistical Analyses

To silence CDKL5, hTERT RPE-1 and hTERT-RPE-1 GFP-CDKL5 cells (5 × 10^4^ cells seeded in 12-well plates) were transfected with 40 nM (final concentration) of 4 small interfering RNAs (siRNAs) targeting CDKL5 obtained from Dharmacon (ON-TARGET plus) using Lipofectamine RNAiMAX transfection reagent (Invitrogen). The Luciferase GL2 Duplex non-targeting siRNA from Dharmacon was used as a negative control. The cells were transfected and serum-starved for 72 hr to induce the formation of primary cilia. The p values are from two-tailed unpaired Student’s t tests.

## Data and Software Availability

The accession numbers for the structures reported in this paper are PDB: 4AGU, 4AAA, 4BBM, 3ZDU, and 4BGQ.

## Supplemental Information

Document S1. Supplemental Experimental Procedures, Figures S1–S5, and Tables S1–S3

Document S2. Article plus Supplemental Information
