# Supplementary material for: CDKL Family Kinases Have Evolved Distinct Structural Features and Ciliary Function
Source: Cell Rep. 2018 Jan 28;22(4):885–94. doi: 10.1016/j.celrep.2017.12.083 (PMC5846859; doi:10.1016/j.celrep.2017.12.083)
Supplement: Document S1. Supplemental Experimental Procedures, Figures S1–S5, and Tables S1–S3 [file mmc1.pdf]

Cell Reports, Volume *22*

Supplemental Information

CDKL Family Kinases Have Evolved

Distinct Structural Features and Ciliary Function

Peter Canning, Kwangjin Park, João Gonçalves, Chunmei Li, Conor J. Howard, Timothy D. Sharpe, Liam J. Holt, Laurence Pelletier, Alex N. Bullock, and Michel R. Leroux

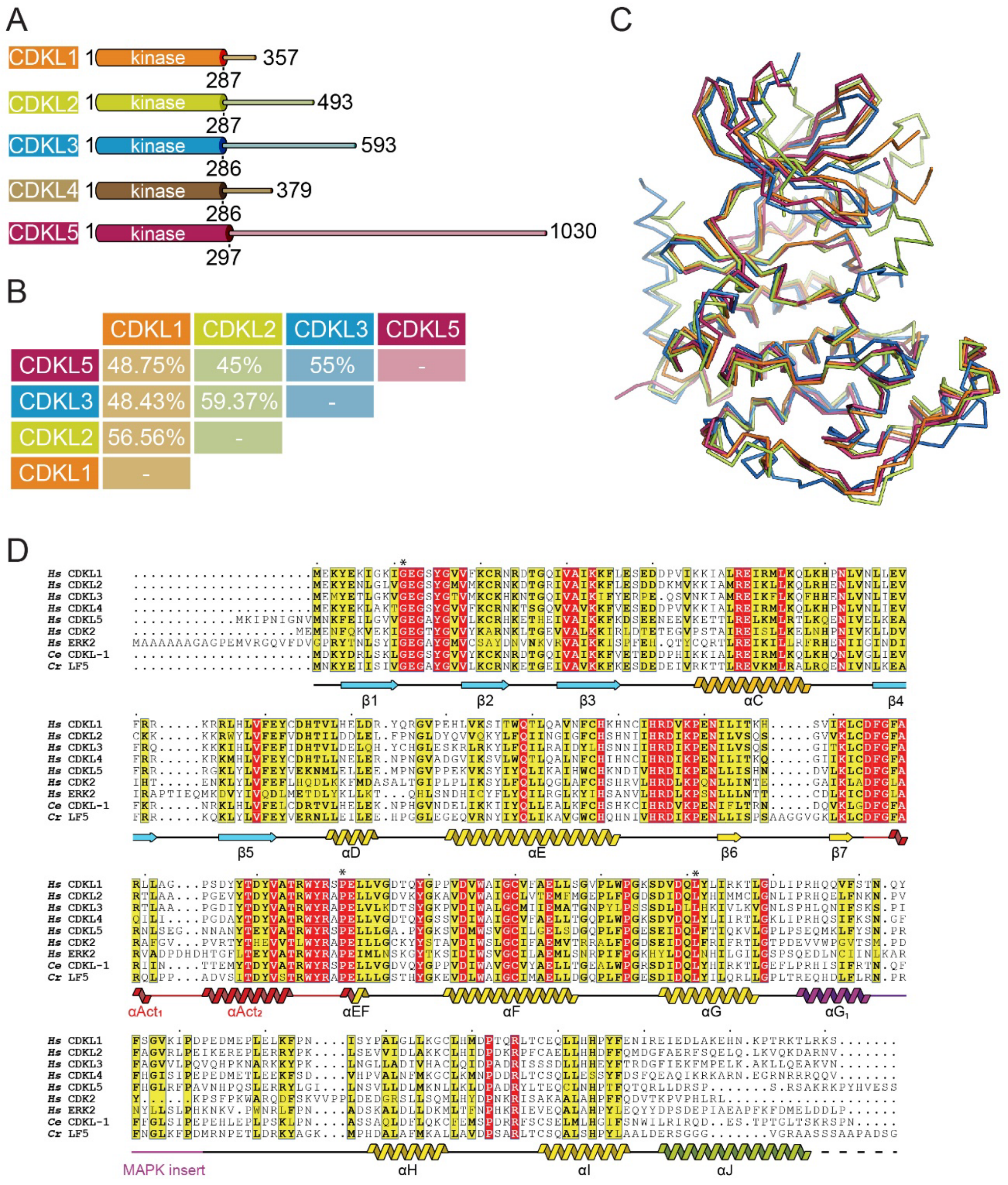

Figure S1. Comparison of the CDKL kinase domain structures. Related to Figure 1.
(**A**) The predicted kinase domains (UNIPROT annotations) of human CDKL1-5 are shown relative to the size of the full-length proteins
(**B**) Pairwise sequence identities between the kinase domains of the four CDKL family members for which structures were solved.
(**C**) Superposition of Cα backbones of all four CDKL kinase domains colored as panel (**B**).
(**D**) Sequence alignment of the kinase domains of *H. sapiens* (*Hs*) CDKL1-5, CDK2, ERK2 (MAPK1), *C. elegans* (*Ce*) CDKL-1 and *Chlamydomonas reinhardtii* (*Cr*) LF5. The secondary structure elements from the CDKL2 complex with TCS2312 are shown. Asterisks mark the sites of disease-causing mutations analyzed in this study.

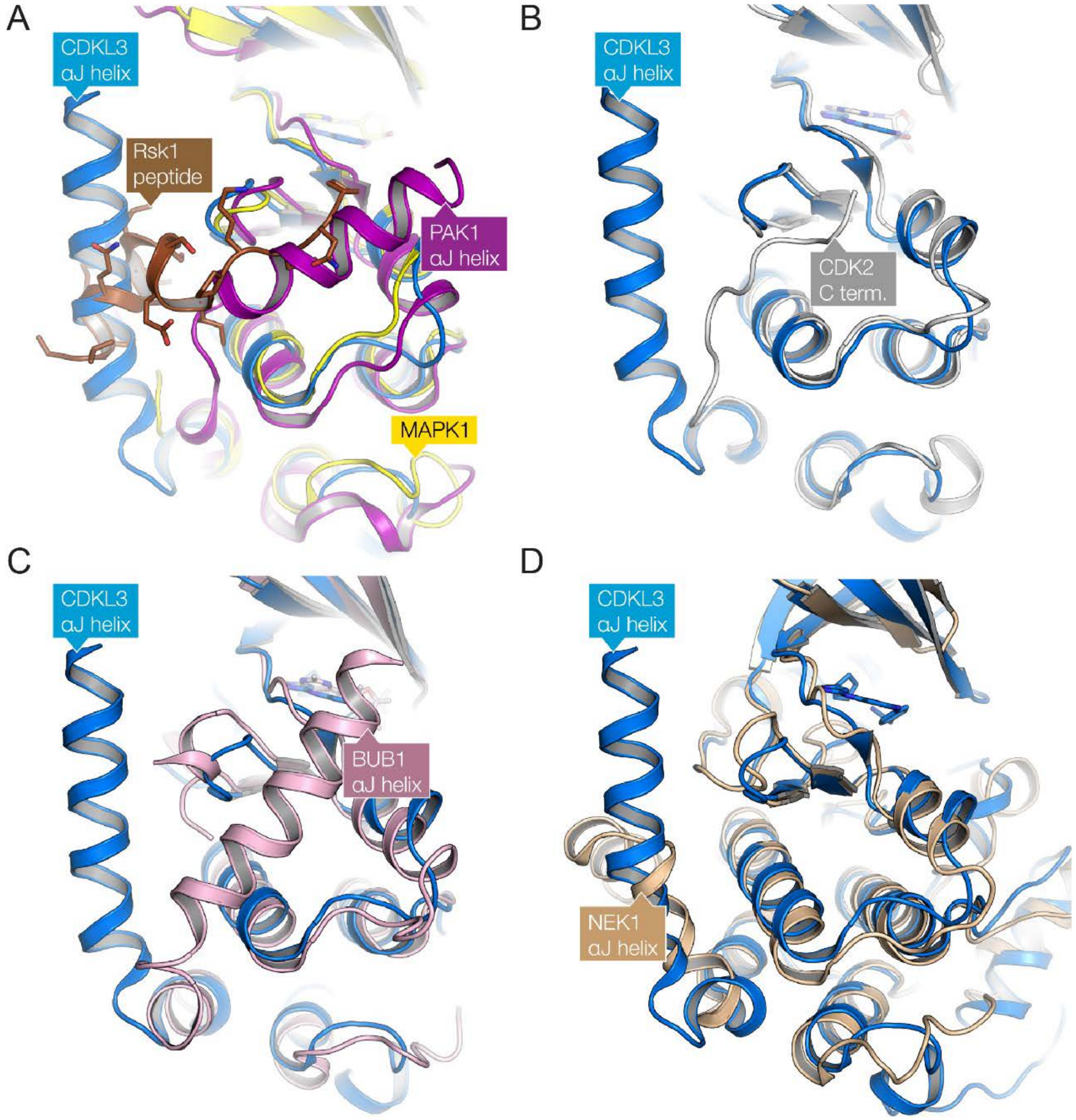

Figure S2. The CDKL αJ helix occupies an unusual position compared to the C-terminal extensions in other protein kinases. Related to Figure 1.

(**A**) Comparison of the MAPK1 (ERK2) peptide docking site with other kinases. MAPK1 (yellow, PDB ID: 3TEI), CDKL3 (blue) and PAK1 (magenta, PDB ID: 1F3M) are shown as cartoons and superimposed, and the MAPK1-bound Rsk1 peptide is shown as a cartoon with stick side-chains. PAK1 is displayed as a representative member of the STE family kinases.

(**B**) Superposition of the CDKL3 (blue) and CDK2 (gray, PDB ID: 1QMZ) structures. The CDK2 C-terminus occupies a site similar to the Rsk1 peptide bound to MAPK1, whereas this site is largely vacated in the CDKL3 structure by the unusual positioning of the αJ helix.

(**C**) Superposition of the CDKL3 (blue) and BUB1 (pink, PDB ID: 4QPM) structures. The BUB1 C-terminus occupies a similar position to PAK1 above, but displays a longer helix. BUB1 is an example of a remote kinase falling outside the major kinase subfamilies.

(**D**) Superposition of the CDKL3 (blue) and NEK1 (bronze, PDB ID: 4APC) structures. The NEK1 C-terminus folds more similarly to CDKL3 at the N-terminus of the αJ helix, but then turns perpendicularly to follow the path of the αE helix. NEK kinases also form a remote kinase clade falling outside the major kinase subfamilies.

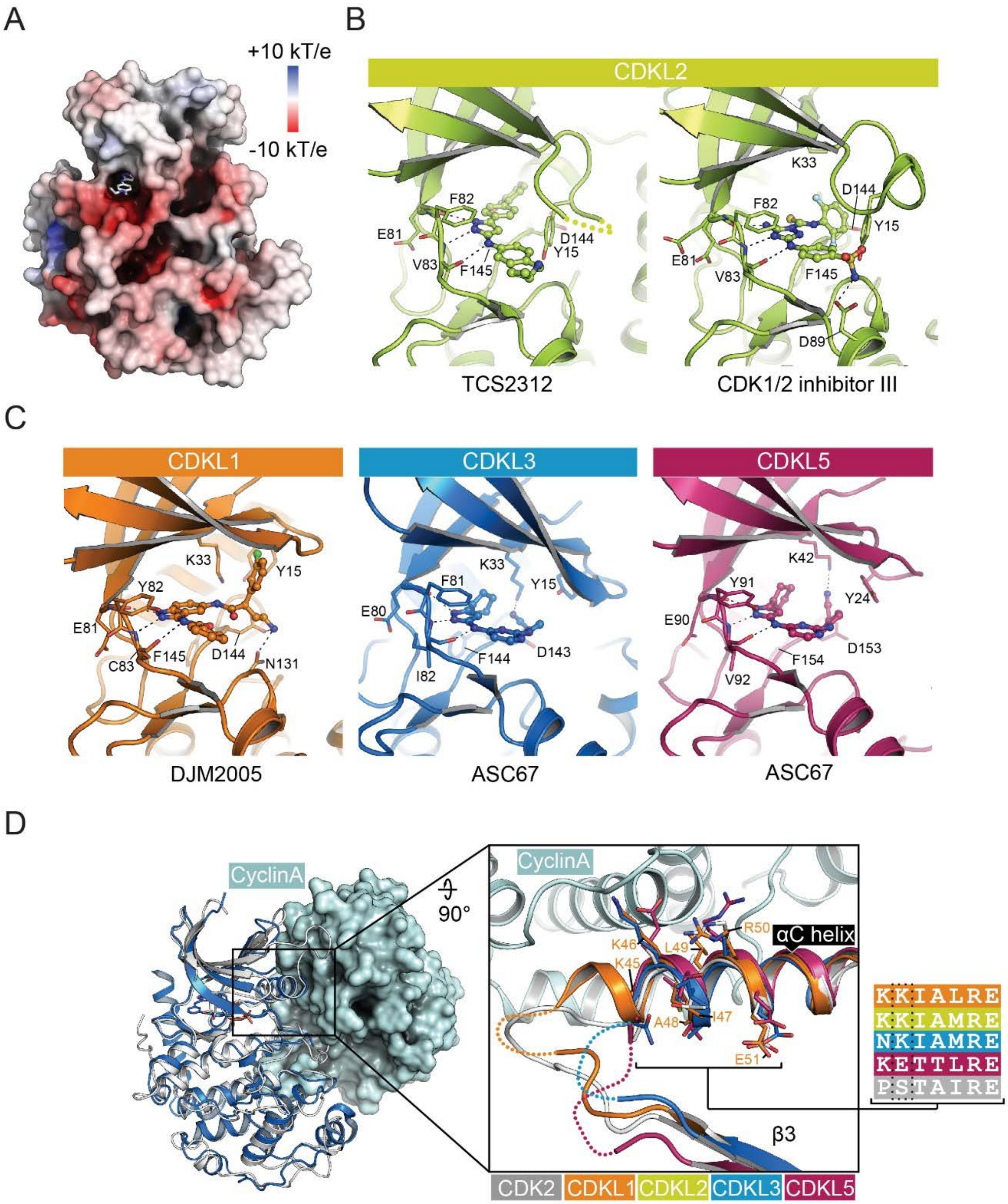

Figure S3. Inhibitor interactions and partial conservation of the CDK2 PSTAIRE motif in the CDKL family structures. Related to Figure 2.

(**A**) Surface representation of CDKL2 (PDB ID: 4BBM) colored by electrostatic potential.

(**B**) The ATP-binding pocket of CDKL2 with the two bound inhibitors. The Kinase domain is shown as a cartoon and the inhibitors as ball-and-stick representations. Key residues involved in inhibitor binding are shown as sticks and labeled.

(**C**) The ATP binding pockets of CDKL1, 3 and 5, shown as described for (**B**).

(**D**) Comparison of the CDKL3 structure (blue) with the structure of CDK2 (gray) bound to Cyclin A (pale blue surface representation) (PDB ID: 1FIN). The inset panel shows the superposition of the αC region of CDK2 and

indicated CDKLs; the alignment panel shows the sequences of these proteins across this region. Side chains are shown as sticks and numbering shown for residues in CDKL1. The sidechains marked in bold have been modeled as they were not built during crystal structure refinement due to poor electron density.

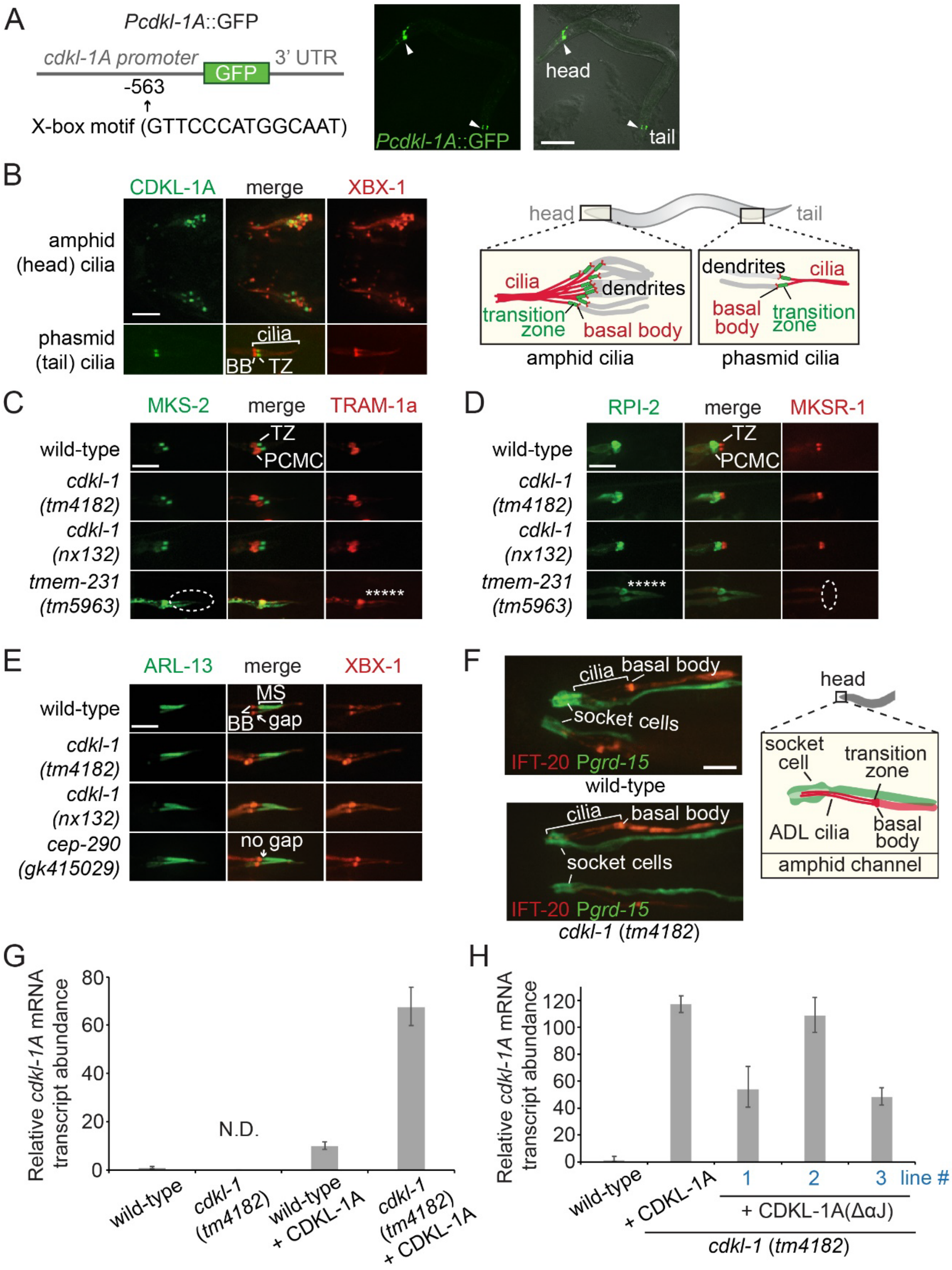

Figure S4. CDKL-1, the sole *C. elegans* CDKL family member, localizes at the transition zone but does not appear to influence cilium gate function. Related to Figure 3.

(**A**) The *C. elegans cdkl-1A* promoter-GFP transcriptional fusion shown in the schematic (and containing a ciliogenic X-box regulatory motif) is specifically expressed in head and tail ciliated sensory neurons (arrow head), as shown in the fluorescent and DIC overlay images. Scale bar, 100 μm.
(**B**) GFP-tagged CDKL-1A specifically localizes at the transition zone (TZ) in head and tail cilia; the tdTomato-XBX-1 IFT protein serves to mark the basal body (BB) and axonemes in head (amphid) and tail (phasmid) sensory neuron cilia (see schematic). Scale bar, 4 μm.
(**C-E**) Disruption of CDKL-1 does not visibly influence cilium gate function. The plasma membrane proteins TRAM-1a (**C**) and RPI-2 (**D**) concentrate just outside of cilia at the periciliary membrane compartment (PCMC) in wild-type animals and *cdkl-1* mutants, indicating the presence of a normal diffusion barrier; these proteins leak into cilia in transition zone (TZ) mutant (*tmem-231*) (asterisk) where TZ function is known to be compromised. In the *tmem-231* mutant, the TZ protein co-markers, MKS-2 and MKSR-1, mislocalize (dotted ellipse). ARL-13 (**E**) localizes correctly in the middle segment (MS) in wild-type and *cdkl-1* mutants, indicating an intact ciliary gate. However, ARL-13 leaks out of cilium in the TZ mutant known to affect gate function, *cep-290* (*gk415029*). XBX-1 marks the basal body (BB) and axoneme. PCMC, periciliary membrane compartment. Scale bar, 4 μm.
(**F**) The cilia of ADL in L3 larvae of wild-type and *cdkl-1* (*tm4182*) mutant are correctly guided into amphid channels (Inglis et al., 2007). The schematic depicts how ADL cilia (red) penetrate an amphid channel formed by the sheath and socket (green) cells. The fluorescence images from wild-type or *cdkl-1* mutant animals show a cilium marker (tdTomato-tagged IFT-20 expressed in the neuron using the P*srh-220* promoter) and socket cell marker (GFP expressed using the *grd-15* promoter). Scale bar, 4μm.
(**G-H**) Relative mRNA transcript level of *cdkl-1A* or *cdkl-1A(ΔαJ)* in indicated strains. The gene expression was normalized to a reference gene (*tba-1*). The level of *cdkl-1A* transcript in wild-type is set to 1. N.D., no detection

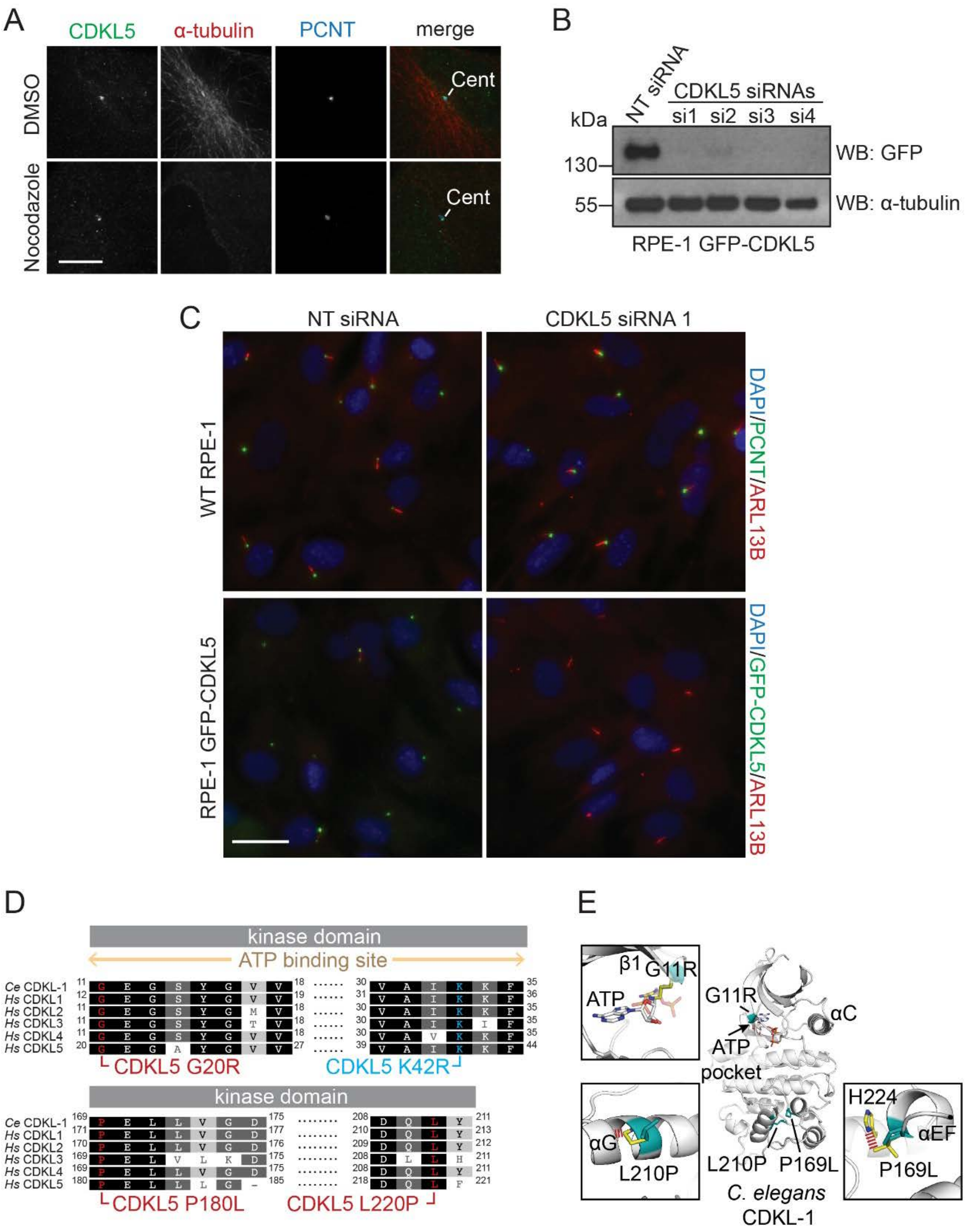

Figure S5. Negative effect on ciliogenesis of human CDKL5 overexpression in RPE-1 cells and the structural modeling of human CDKL5 pathogenic mutations in *C. elegans* CDKL-1. Related to Figure 4.
(**A**) Immunofluorescence analysis of hTERT RPE-1 GFP-CDKL5 cells were treated with DMSO or 30µM nocodazole for 1h after which they were fixed and stained with antibodies against GFP, α-tubulin and PCNT. Cent, centrosome, Scale bar, 10µm.
(**B**) Western blot analysis of GFP-CDKL5 levels in hTERT RPE-1 GFP-CDKL5 cells transfected with control non-targeting (NT) or CDKL5-directed siRNAs for 72h.

(**C**) Immunofluorescence analysis of control (NT siRNA) and CDKL5 siRNA treated hTERT RPE-1 and hTERT RPE-1 GFP-CDKL5 cells transfected with the indicated siRNAs for 72h and stained with the indicated antibodies. DNA was stained with DAPI. Scale bar, 25µm.
(**D**) Conserved CDKL5 missense mutations associated with neurological disorders (red) and catalytic lysine residue (blue) highlighted in a partial sequence alignment of human (*Hs*) and *C. elegans* (*Ce*) CDKL kinase domains.
(**E**) Locations of missense mutation residues (cyan) in a homology model of the *C. elegans* CDKL-1A structure (gray) prepared using SWISS-MODEL (Biasini et al., 2014). Inset boxes show wild-type (cyan) and mutant side chains (yellow) modelled using the DUET server (Pires et al., 2014). Steric clashes are indicated by red bars.

Table S1. Selected thermal shift screening results. Related to Figure 1 and Supplemental Experimental Procedures.

**CDKL1**			
Compound	Supplier	Supplier ID	Tm shift (°C)
ASC67	K. Shokat UCSF	ASC67	8.4
DJM2005	K. Shokat UCSF	DJM2005	7.5
K252a	Calbiochem	420298	6.9
Staurosporine	AXXORA	S-9300	6.6
Indirubin E804	Calbiochem	402081	6.1
TCS 2312	Tocris	3038	5.6
Cdk1/2 Inhibitor III	Calbiochem	217714	4.9
PI3K Inhibitor IV	Calbiochem	528111	4.7
Cdk2/9 Inhibitor	Calbiochem	238806	4.3
Curcumin	Calbiochem	239802	3.9
Indirubin-3monoxime	Calbiochem	402085	3.8
Wee1/Chk1 inhibitor	Calbiochem	681637	3.4
GSK-3 inhibitor X	Calbiochem	361551	3.2
IKK Inhibitor VII	Calbiochem	401486	3.1
JNK inhib V	Calbiochem	420129	3.1
BIM 8	AXXORA	B-7457	2.7
BIM 9	AXXORA	B-7334	2.2
VEGFR 2/3 Inhibitor	Calbiochem	676499	1.9
Quercetin	Calbiochem	551600	1.6
GSK inhibitor XIII	Calbiochem	361555	1.5
**CDKL2**			
Compound	Supplier	Supplier ID	Tm shift (°C)
TCS 2312	Tocris	3038	6.6
ASC67	K. Shokat UCSF	ASC67	5.8
K252a	Calbiochem	420298	5.6
Curcumin	Calbiochem	239802	5.5
Flt3 Inhibitor III	Calbiochem	343022	5.5
Cdk1/2 Inhibitor III	Calbiochem	217714	5.4
Dovitinib	LC Labs	D-3608	4.0
Cdk2 Inhibitor II	Calbiochem	219445	3.6
BIM 9	AXXORA	B-7334	3.6

Quercetin	Calbiochem	551600	3.4
SU9516	Calbiochem	572650	3.3
Cdk2/9 Inhibitor	Calbiochem	238806	3.1
GSK-3 Inhibitor XVI	Calbiochem	361559	3.0
SB 218078	Calbiochem	559402	2.6
Staurosporine	AXXORA	S-9300	2.5
Aurora/Cdk Inhibitor	Calbiochem	189406	2.2
Oxindole I	Calbiochem	499600	2.2
Indirubin E804	Calbiochem	402081	2.1
DJM2005	K. Shokat UCSF	DJM2005	2.1
PF 573228	Tocris	3239	2.0
**CDKL3**			
Compound	Supplier	Supplier ID	Tm shift (°C)
ASC67	K. Shokat UCSF	ASC67	7.1
K252a	Calbiochem	420298	5.4
Cdk2/9 Inhibitor	Calbiochem	238806	5.3
Quercetin	Calbiochem	551600	5.1
PI3K Inhibitor VIII	Calbiochem	528116	5.0
Curcumin	Calbiochem	239802	4.5
SU9516	Calbiochem	572650	4.2
SB 218078	Calbiochem	559402	3.6
DJM2005	K. Shokat UCSF	DJM2005	3.1
Apigenin	Calbiochem	178278	2.6
Raf1 kinase inhibitor II	Calbiochem	553011	2.4
Cdk1/2 Inhibitor III	Calbiochem	217714	2.3
Indirubin E804	Calbiochem	402081	2.2
Flt3 Inhib III	Calbiochem	343022	2.2
Staurosporine	AXXORA	S-9300	2.0
Debromohymenialdisine	Calbiochem	252010	1.8
Cdc7/CDK9 inhibitor	Calbiochem	217707	1.7
Alsterpaullone	Calbiochem	126870	1.6
Cdk2 Inhibitor II	Calbiochem	219445	1.4
TCS 2312	Tocris	3038	0.4
**CDKL5**			
Compound	Supplier	Supplier ID	Tm shift (°C)

ASC67	K. Shokat UCSF	ASC67	7.4
K252a	Calbiochem	420298	5.3
Wee1/Chk1 inhibitor	Calbiochem	681637	5.3
Indirubin E804	Calbiochem	402081	5.0
SU9516	Calbiochem	572650	4.6
BIBX1382	Calbiochem	324832	4.5
Flavopiridol	AXXORA	ALX-430	4.5
Quercetin	Calbiochem	551600	4.5
Curcumin	Calbiochem	239802	4.2
Staurosporine	AXXORA	S-9300	4.0
Apigenin	Calbiochem	178278	3.8
Cdk2/9 Inhibitor	Calbiochem	238806	3.8
Cdk1/2 Inhibitor III	Calbiochem	217714	3.3
Oxindole I	Calbiochem	499600	3.3
BIM I	Calbiochem	203291	3.3
Cdc7/CDK9 inhibitor	Calbiochem	217707	3.3
5-Iodotubercidin	Calbiochem	407900	3.2
Alsterpaullone	Calbiochem	126870	2.9
TCS 2312	Tocris	3038	0.3
DJM2005	K. Shokat UCSF	DJM2005	0.1

Table S2. Crystallographic data collection and refinement statistics. Related to Figure 1.

Protein	CDKL1	CDKL2	CDKL2	CDKL3	CDKL5
**Inhibitor**	DJM2005	Cdk1/2 Inhibitor III	TCS2312	ASC67	ASC67
**Data collection**					
**Beamline**	I24	I02	I04-1	I04	I03
**Wavelength (Å)**	0.9778	0.9795	0.9173	0.9611	0.9795
**Resolution range (Å)**	29.78 - 2.4 (2.53 - 2.4)	42.14 - 1.6 (1.69 - 1.6)	39.39 - 2.0 (2.072 - 2.0)	54.54 - 2.2 (2.27 - 2.2)	55.02 - 2.0 (2.05 - 2.0)
**Space group**	$P\ 3_2$	$P\ 2_1\ 2_1\ 2_1$	$P\ 1$	$P\ 3_1\ 2\ 1$	$P\ 2_1\ 2_1\ 2_1$
**Cell dimensions**					
**a, b, c (Å)**	124 124 49.3	67.8 70.2 83.7	31.6 68 82.3	63.9 63.9 163.6	51.9 65.5 102.9
**α, β, γ (°)**	90 90 120	90 90 90	71.5 89.9 89	90 90 120	90 90 90
**Total reflections**	157705 (22867)	733038 (60837)	148975 (11552)	106415 (9026)	293728 (15016)
**Unique reflections**	33165 (4877)	53461 (7693)	42854 (3326)	20446 (1723)	23934 (2187)
**Multiplicity**	4.8 (4.7)	13.7 (7.9)	3.5 (3.5)	5.2(5.2)	12.2 (8.9)
**Completeness (%)**	99.98 (100.00)	100.00 (100.00)	97.6 (98.4)	99.9 (100)	99.04 (92.55)
**Mean I/sigma(I)**	8.2 (2)	13.4 (2.1)	11.2 (2.4)	10.7 (2.5)	22.6 (4.8)
**Wilson B-factor**	44.82	20.24	16.14	32.45	24.76
**R-meas**	0.123 (0.777)	0.148 (1.223)	0.153 (1.017)	0.117 (0.782)	0.079 (0.414)
**CC1/2**	0.93 (0.738)	0.999 (0.645)	0.945 (0.696)	0.997 (0.906)	0.999 (0.935)
**Phasing model(s)**	4AAA	3NIZ	4AAA	4AAA, 4AGU	4BBM
**Mols ASU**	3	1	2	1	1
**Refinement**					
**R-work**	0.1865	0.1803	0.2425	0.2142	0.1738
**R-free**	0.2261	0.2082	0.269	0.2625	0.2014
**Number of atoms**	6810	2826	4956	2516	2413
**macromolecules**	6399	2515	4686	2319	2217
**ligands**	96	52	80	38	44
**water**	315	259	190	159	152
**Protein residues**	831	295	606	297	276
**RMS(bonds) (Å)**	0.013	0.014	0.009	0.013	0.009
**RMS(angles) (Å)**	1.66	1.53	1.24	1.49	1.29
**Ramachandran favored (%)**	98	98	97	97	98
**Ramachandran outliers (%)**	0	0	0	0	0

**Clashscore**	1.89	4.49	2.37	2.81	2
**Average B-factor (Å²)**	60.7	28.8	29.6	56.9	29.5
**macromolecules**	60.6	28	29.6	57.6	29
**ligands**	64.4	27.7	32.4	45.9	32.2
**solvent**	60.5	36.4	29.5	49.5	35.9
**PDB ID**	4AGU	4AAA	4BBM	3ZDU	4BGQ

Table S3. ***C. elegans*** **strains used in this study. Related to Experimental Procedures.**

Strain	Genotype
N2	*Bristol wild-type*
MX1420	*nxEx172[Pbbs-8::tram-1::tdtomato; Pbbs-8::mks-2::gfp; rol-6(su1006)]*
MX1388	*nxEx231[arl-13::gfp; Posm-5::xbx-1::tdtomato; rol-6(su1006)]*
MX1813	*tmem-231*(*tm5963*); *nxEx172[Pbbs-8::tram-1::tdtomato; Pbbs-8::mks-2::gfp; rol-6(su1006)]*
MX1924	*raIs12964[Pgrd-15::gfp]; nxEx256[Psrh-220::ift-20::tdtomato; rol-6(su1006)]*
MX1929	*nxEx969[cdkl-1A::gfp; xbx-1::tdtomato; rol-6(su1006)]*
MX1932	*nxEx250[rpi-2::gfp; mksr-1::tdtomato; rol-6(su1006)]*
MX1938	*cdkl-1(tm4182); nxEx250[rpi-2::gfp; mksr-1::tdtomato; rol-6(su1006)]*
MX1940	*tmem-231*(*tm5963*); *nxEx250[rpi-2::gfp; mksr-1::tdtomato; rol-6(su1006)]*
MX1943	*cdkl-1(tm4182); nxEx231[arl-13::gfp; Posm-5::xbx-1::tdtomato; rol-6(su1006)]*
MX1965	*cep-290*(*gk415029*); *nxEx231[arl-13::gfp; Posm-5::xbx-1::tdtomato; rol-6(su1006)]*
MX2100	*cdkl-1(tm4182); nxEx172[Pbbs-8::tram-1::tdtomato; Pbbs-8::mks-2::gfp; rol-6(su1006)]*
MX2102	*cdkl-1(tm4182)*
MX2246	*cdkl-1(nx132); nxEx172[Pbbs-8::tram-1::tdtomato; Pbbs-8::mks-2::gfp; rol-6(su1006)]*
MX2247	*cdkl-1(nx132); nxEx231[arl-13::gfp; Posm-5::xbx-1::tdtomato; rol-6(su1006)]*
MX2277	*cdkl-1(nx131) (c. G90T, A98G)(p.K33R)*
MX2278	*cdkl-1(nx132) (c. 85ATTGT89del)*
MX2293	*nxEx258[cdkl-1A(K33R)::tdtomato; Pbbs-8::mks-2::gfp; rol-6(su1006)]*
MX2301	*nxEx1193[cdkl-1A(G11R)::tdtomato; Pbbs-8::mks-2::gfp; rol-6(su1006)]*
MX2303	*nxEx1195[cdkl-1A(L210P)::tdtomato; Pbbs-8::mks-2::gfp; rol-6(su1006)]*
MX2304	*nxEx1329[cdkl-1A::tdtomato; Pbbs-8::mks-2::gfp; rol-6(su1006)]*
MX2362	*nxEx1232[cdkl-1A(P169L)::tdtomato; Pbbs-8::mks-2::gfp; rol-6(su1006)]*
MX2397	*cdkl-1(nx132); nxEx250[rpi-2::gfp; mksr-1::tdtomato; rol-6(su1006)]*
MX2415	*cdkl-1(tm4182); raIs12964[Pgrd-15::gfp]; nxEx256[Psrh-220::ift-20::tdtomato; rol-6(su1006)]*
MX2426	*nxIs30[Psrh-220::IFT-20::gfp; cc::gfp]*
MX2427	*cdkl-1(nx132); nxIs30[Psrh-220::ift-20::gfp; cc::gfp]*
MX2428	*cdkl-1(nx131); nxIs30[Psrh-220::ift-20::gfp; cc::gfp]*
MX2429	*cdkl-1(tm4182); nxIs30[Psrh-220::ift-20::gfp; cc::gfp]*
MX2447	*cdkl-1(tm4182); nxIs30[Psrh-220::ift-20::gfp; cc::gfp]; nxEx261[cdkl-1A; coel::rfp]*
MX2471	*nxEx1282[Pcdkl-1A::gfp; rol-6(su1006)]*
MX2527	*nxIs30[Psrh-220::ift-20::gfp; cc::gfp]; nxEx261[cdkl-1A; coel::rfp]*
MX2538	*cdkl-1(tm4182); nxIs30[Psrh-220::ift-20::gfp; cc::gfp]; nxEx1344[cdkl-1A(G11R); coel::rfp] line1*
MX2539	*cdkl-1(tm4182); nxIs30[Psrh-220::ift-20::gfp; cc::gfp]; nxEx1345[cdkl-1A(G11R); coel::rfp] line2*
MX2540	*cdkl-1(tm4182); nxIs30[Psrh-220::ift-20::gfp; cc::gfp]; nxEx1346[cdkl-1A(P169L); coel::rfp] line1*
MX2541	*cdkl-1(tm4182); nxIs30[Psrh-220::ift-20::gfp; cc::gfp]; nxEx1347[cdkl-1A(L210P); coel::rfp] line1*
MX2542	*cdkl-1(tm4182); nxIs30[Psrh-220::ift-20::gfp; cc::gfp]; nxEx1348[cdkl-1A(L210P); coel::rfp] line2*
MX2544	*cdkl-1(tm4182); nxIs30[Psrh-220::ift-20::gfp; cc::gfp]; nxEx2544[cdkl-1A(P169L); coel::rfp] line3*
MX2546	*cdkl-1(tm4182); nxIs30[Psrh-220::ift-20::gfp; cc::gfp]; nxEx2546[cdkl-1A(P169L); coel::rfp] line2*
MX2560	*cdkl-1(tm4182); nxIs30[Psrh-220::ift-20::gfp; cc::gfp]; nxEx2560[cdkl-1A; coel::rfp] line1*
MX2561	*cdkl-1(tm4182); nxIs30[Psrh-220::ift-20::gfp; cc::gfp]; nxEx2561[cdkl-1A; coel::rfp] line2*
MX2642	*cdkl-1(tm4182); nxIs30[Psrh-220::ift-20::gfp; cc::gfp]; nxEx2642[cdkl-1A; coel::rfp] line3*

MX2761	*nxEx2761[cdkl-1A(ΔαJ)::mNeonGreen; Posm-5::xbx-1::tdTomato; rol-6(su1006)]*
MX2764	*cdkl-1(tm4182); nxIs30[Psrh-220::ift-20::gfp; cc::gfp]; nxEx2764[cdkl-1A(ΔαJ); coel::rfp] line1*
MX2765	*cdkl-1(tm4182); nxIs30[Psrh-220::ift-20::gfp; cc::gfp]; nxEx2765[cdkl-1A(ΔαJ); coel::rfp] line2*
MX2766	*cdkl-1(tm4182); nxIs30[Psrh-220::ift-20::gfp; cc::gfp]; nxEx2766[cdkl-1A(ΔαJ); coel::rfp] line3*

SUPPLEMENTAL EXPERIMENTAL PROCEDURES

Cloning of human CDKL kinase domains

Bacmids were prepared in *E. coli* strain DH10Bac and used to generate baculoviruses in Sf9 insect cells. For activity assays, wild-type sequences (residues corresponding to CDKL1, 1-357; CDKL1(ΔαJ), 1-287; CDKL2, 1-312; CDKL2(ΔαJ), 1-287; CDKL3, 1-313; CDKL3(ΔαJ), 1-286; CDKL5, 1-831; CDKL5(ΔαJ), 1-299) were cloned into a 2 μm P_{GAL1}-kinase-TAP plasmid (pRSAB1234 backbone, originally a gift from Erin O'Shea) for expression in *S. cerevisiae.*

Expression and purification of human CDKL kinase domains

For structural studies, CDKL1 was expressed in *E. coli* strain BL21(DE3)-R3-pRARE using 0.4 mM isopropyl 1-thio-β-D-galactopyranoside for overnight induction at 18°C. Harvested cells were resuspended in binding buffer (50 mM HEPES pH 7.5, 500 mM NaCl, 5% glycerol, 5 mM Imidazole) supplemented with protease inhibitor cocktail set V (Calbiochem) at 1:1000 dilution and 1 mM tris(2-carboxyethyl)-phosphine (TCEP). To express CDKL2, CDKL3 and CDKL5, baculoviruses were used to infect Sf9 cells grown in suspension at a density of $2x10^6$ cells/mL in Insect-Xpress media (Lonza). Cells were incubated at 27°C and harvested 72 hr post-infection. Harvested cells were resuspended in binding buffer supplemented similarly with protease inhibitors and 1 mM TCEP. All cells were disrupted by sonication or high-pressure homogenization. Polyethylenimine (PEI) was added to a final concentration of 0.5% to precipitate DNA and the cell lysate clarified by centrifugation at 21,000 RPM at 4°C for 1 hr. Proteins were purified by nickel-affinity and size exclusion chromatography. Tobacco etch virus protease A (TEV) was used to proteolytically cleave the polyhistidine tag overnight at 4°C from all proteins except CDKL2.

For activity assays, W303 *S. cerevisiae* strains were grown overnight to log phase in SC-URA media containing 2% raffinose (Sigma), and then expression of N-terminal kinase domains was induced by addition of 2% galactose (Sigma) for 4 hr at 30°C. Cells were harvested by centrifugation at 8000 RPM, cell pellet washed and resuspended in 1x cell volume of lysis buffer containing 25 mM HEPES pH 8.0, 300 mM NaCl, 0.1% NP-40, 30 mM EGTA, 1 mM EDTA, and a protease/phosphatase inhibitor set was added immediately prior to harvest including 80 mM β-glycerophosphate, 50 mM NaF, 1 mM DTT, 1 mM Na_3O_4V, and 1 mM PMSF. The cell slurry was slowly dripped into liquid nitrogen to produce frozen pellets. These pellets were then pulverized in a cryogenic ball mill (Retsch MM301 with 50 ml stainless steel grinding jars) by five rounds of agitation at 15 Hz for 2 min, re-cooling the grinding jars in liquid N_2 after each cycle. The grindate was then thawed and cell debris was cleared by centrifugation at 8000 rpm for 30 min followed by sequential filtration through 2.7 and 1.6 μm Whatman GD/X filters (GE). C-terminally TAP-tagged kinases were immobilized on IgG Sepharose 6 Fast Flow beads (GE). These beads (~500 μL slurry per 1 L culture) had been pre-equilibrated in lysis buffer with inhibitors and were then incubated with lysate for 1 hr at 4°C. Bound beads were then loaded into a disposable Bio-Spin column (cat. #732-6008; BioRad) by pipette and washed with 20 mL total wash buffer (lysis buffer + 10% glycerol, 1 mM DTT) at 4°C. The column was then rotated for 20 min at 23°C in 700 μL wash buffer as a final wash to mimic elution conditions. The bound protein was then cleaved from the IgG beads by TEV protease in 600 μL elution buffer (0.21 mg/mL TEV protease [QB3 MacroLab, UC Berkeley], 25 mM HEPES pH 8.0, 310 mM NaCl, 0.09% NP-40, 26.9 mM EGTA, 0.9 mM EDTA, 1 mM DTT, and 10% glycerol) for 1 hr at 23°C.

Thermal melting shift assay

Proteins were buffered in 10 mM HEPES pH 7.5, 500 mM NaCl, 5% glycerol and a 1:1000 dilution of SYPRO Orange fluorescent dye (Invitrogen, CA). Proteins at 2 μM were mixed with inhibitor compounds at 10 μM and thermal melting shift (Tm Shift) assays run using a Mx3005p real-time PCR machine (Agilent) as described (Niesen et al., 2007).

Structure determination

Crystals were grown using the sitting-drop vapor-diffusion technique and cryo-protected by addition of 25% ethylene glycol before vitrification in liquid nitrogen. Crystallization conditions and co-crystallized inhibitors are listed in Table EV5. Diffraction data were collected at 100K using beam lines at Diamond Light Source, UK. Data were indexed and integrated using XDS (Kabsch, 2010) or MOSFLM (Leslie and Powell, 2007) and scaled using SCALA (Evans, 2006; Evans, 2011) or AIMLESS (Evans and Murshudov, 2013) in the CCP4 suite of programs (Winn et al., 2011). Phases were found using molecular replacement in PHASER (McCoy et al., 2007). SCULPTOR was used to optimize PDB entries for use as search models. The structures were built using PHENIX. AUTOBUILD

(Adams et al., 2010) and then refined and modified using alternate rounds of REFMAC5 (Murshudov et al., 2011) or BUSTER (Bricogne et al., 2011) and COOT (Emsley and Cowtan, 2004; Emsley et al., 2010). TLS groups were determined using the TLSMD server (Painter and Merritt, 2006). The refined structures were validated with MolProbity (Chen et al., 2010) and the atomic coordinate files deposited in the Protein Data Bank (PDB) with Autodep (Yang et al., 2004). Structure figures were prepared with PyMOL (The PyMOL Molecular Graphics System, Version 1.2r3pre, Schrödinger, LLC) and electrostatic surface maps calculated using DelPhi (Li et al., 2012). Sequence alignments were prepared with Clustal Omega (Sievers and Higgins, 2014) and ESPript (http://espript.ibcp.fr) (Robert and Gouet, 2014).

In vitro kinase assays
Peptide substrate (acetyl-RPRSPGARR-amide) was obtained from Tufts University Core Facility and added to a final concentration of 200 μM to start the reaction, or at variable concentration for Michaelis–Menten curves. Reactions were aliquoted onto Whatman P81 phosphocellulose (GE) strips, which were then quenched and washed 5 x in 75 mM phosphoric acid to remove free [γ-^{32}P] ATP. Samples were dried on a slab gel dryer (Model 1125B; BIORAD) and exposed to a phosphor screen (Molecular Dynamics) to determine the rate of [γ-^{32}P] ATP incorporation. Phosphor screens were analyzed with a Typhoon 9400 scanner (Amersham) using ImageQuant software (GE). Final Image quantification was performed using ImageJ (http://imagej.nih.gov/ij/). Data were fit by nonlinear regression to the Michaelis–Menten model $V_0 = V_{max}*[S]/K_M+[S]$ using Prism (GraphPad software) and Matlab (MathWorks).

C. elegans mutant and transgenic strains
C. elegans cep-290 (*gk415029*), *tmem-231* (*tm5963*) and *cdkl-1 (tm4182)* mutant strains were obtained from the *C. elegans* Gene Knockout Consortium or National BioResource Project. *cdkl-1* kinase-dead (*nx131*, p. K33R) and *cdkl-1* null (*nx132,* c. 85-89 deletion) mutants were generated with the CRISPR/CAS9 system (Friedland et al., 2013; Kim et al., 2014). All mutant strains were outcrossed to N2 (wild-type) strain at least five times and genotyped with single-worm PCR. The *cdkl-1* CRISPR guide RNA sequence (5'-AGGGATACTGGACAAATTG-3') was predicted by the CRISPR Design Tool (http://crispr.mit.edu/) and was substituted for *unc-119* sequence in pU6::unc-119 small-guide RNA (sgRNA) vector (Addgene #46169) (Friedland et al., 2013) by site-directed mutagenesis using overlap extension PCR (Zoller, 1991). The *nx131* allele was made by CRISPR-mediated homologous recombination. 2Kb homologous donor DNA template of *cdkl-1A* harboring two mutations, V30V (silence mutation in PAM site) and K33R (kinase-dead), was amplified by stitch-PCR (Reikofski and Tao, 1992) and cloned into a pJET1.2 vector. 50ng/μl each of *cdkl-1* sgRNA vector, Cas9 plasmid (Addgene #46168), pRF4::*rol-6(su1006)* and with or without the *cdkl-1* donor plasmid were mixed and injected into 20 young adult worms. F1 heterozygous (roller) and F2 homozygous (non-roller) worms of either the *nx131* or the *nx132* mutant were identified by tetra-primer ARMS-PCR (Ye et al., 2001) and confirmed by DNA sequencing.

Transgenic animals carrying separate extrachromosomal DNA arrays with different mutant backgrounds were created by worm microinjection method and classical genetic methods. The extrachromosomal array [*Psrh220::ift-20::gfp*; *cc::gfp*] was integrated into the chromosome with 1.5 krads of X-ray irradiation for 135 seconds at 145kilovolts/5milliamps (TORREX150D X-Ray Inspection System) for ADL ciliary length measurement and analysis (McKay et al., 2003). The X-ray integrated strains were outcrossed to wild-type for six times to remove X-ray-induced mutations.

Lentiviral production and generation of the hTERT RPE-1 GFP-CDKL5 stable cell line
For the production of lentiviral particles, HEK293T cells were co-transfected with pHR-SIN-SFFV-GFP-CDKL5 and the second generation packaging (pCMV-dR8.74psPAX2) and envelope (pMD2.G) plasmids using the Lipofectamine® 3000 transfection reagent (Invitrogen) according to the manufacturer's instructions. Lentiviral particles in conditioned media from HEK 293T cells, collected at 48h post transfection, were used to transduce hTERT RPE-1 cells. GFP positive cells were cell sorted to establish the final hTERT RPE-1 GFP-CDKL5 cell line.

Human cell lines and growth conditions
HEK 293T cells were grown in Dulbecco's modified Eagle's medium (DMEM) supplemented with 10% fetal bovine serum (FBS) and GlutaMAX™. hTERT RPE-1 and hTERT RPE-1 GFP-CDKL5 were grown in DMEM/F12 medium supplemented with 10% FBS. To induce ciliogenesis, hTERT RPE-1 and hTERT RPE-1 GFP-CDKL5 cells were cultured in DMEM/F12 medium without FBS. All cells were cultured in a 5% CO_2 humidified atmosphere at 37°C.

Western blot analyses
For western blot analysis, the cells were lysed in Laemmli buffer and treated with benzonase nuclease (Sigma-Aldrich). Whole cell lysates were incubated at 95ºC for 5 min and loaded onto a 10% SDS-PAGE gel. The proteins were separated by electrophoresis and then transferred to a PVDF membrane (Immobilon-P, Millipore). Membranes were incubated with primary antibodies against GFP (Roche, 11 814 460 001) and α-tubulin (Sigma-Aldrich, T6199) in TBST (TBS, 0.1% Tween-20) in 5% skim milk powder (Bioshop). Blots were washed 3x 10 min in TBST, and then incubated with secondary HRP-conjugated antibodies. After being washed again in TBST, the western blots were developed using SuperSignal reagents from Thermo Scientific.

Nocodazole treatment
hTERT RPE-1 GFP-CDKL5 cells were incubated in the presence of DMSO (control) or 30µM nocodazole for 1 h to promote the depolymerization of microtubules. After the treatment, the cells were washed with PBS and processed for immunofluorescence analysis.

Immunofluorescence microscopy
For immunofluorescence, the cells were fixed with ice-cold methanol (10 min at -20°C), blocked with 0.2% Fish Skin Gelatin (Sigma-Aldrich) in 1x PBS (20 min), incubated with the primary antibodies in blocking solution (1h), washed with blocking solution and incubated with fluorophore-conjugated secondary antibodies and DAPI (0.1µg/ml) in blocking solution (1h). After a final wash in blocking solution the coverslips were mounted on glass slides by inverting them onto mounting solution (ProLong Gold antifade, Molecular Probes). The primary antibodies used were: anti-GFP (Roche, 11 814 460 001), anti-ARL13B (Proteintech, 17711-1), anti-PCNT (Santa Cruz Biotechnology, sc-28143), anti-IFT88(Proteintech, 13967-1-AP), anti-polyglutamylated tubulin (GT335, AdipoGen AG-20B-0020), and α-tubulin (Sigma-Aldrich, T6199). The secondary antibodies used were: Alexa488/594/647 anti-mouse, anti-rabbit and anti-goat antibodies from Life Technologies. The cells were imaged either with a 20 x air objective (0.75 NA; **Figure S5C**) or a 60 x oil-immersion objective (1.42 NA; **Figures 4A,B** and **S5A**) on a Deltavision Elite DV imaging system equipped with a sCMOS 2048x2048 pixels2 camera (GE Healthcare). When the 60 x objective was used, z stacks (0.2 µm apart) were collected, deconvolved using softWoRx (v5.0, Applied Precision) and are shown as maximum intensity projections (pixel size 0.1064 µm).

Phylogenetic analysis
The phylogram showing human and *C. elegans* CDKL protein evolutionary relationships was generated using PHYML (bootstrap 1,000) at http://www.atgc-montpellier.fr/phyml and www.phylogeny.fr (Altschul et al., 1997; Chevenet et al., 2006; Dereeper et al., 2010; Dereeper et al., 2008; Edgar, 2004; Guindon et al., 2010). Branch support values (%) are displayed. Protein sequences: *Hs* CDKL1 (Uniprot Q00532), *Hs* CDKL2 (Q92772), *Hs* CDKL3 (Q8IVW4), *Hs* CDKL4 (Q5MAI5), *Hs* CDKL5 (O76039) and *Ce* CDKL-1 (Q9U2H1).

Quantitation of cdkl-1A transcript
cdkl-1A transcript levels were measured using quantitative real-time PCR. cDNA was prepared using Invitrogen SuperScript III reverse transcriptase by following manufacturer's protocol. The RT-PCR was performed using SsoAdvanced Universal SYBR Green Supermix (Biorad) and the StepOne Real-Time PCR System. The relative levels of *cdkl-1A* mRNA transcripts were compared to wild-type which was set to 1. Primers: tba-1 forward (tcaacactgccatcgccgcc) and reverse (tccaagcgagaccaggcttcag), cdkl-1 forward (agatttcggatttgctcgaa) and reverse (gcatatacacatcctacagccc).

SUPPLEMENTAL REFERENCES

Adams, P.D., Afonine, P.V., Bunkoczi, G., Chen, V.B., Davis, I.W., Echols, N., Headd, J.J., Hung, L.W., Kapral, G.J., Grosse-Kunstleve, R.W., et al. (2010). PHENIX: a comprehensive Python-based system for macromolecular structure solution. Acta Crystallogr. D Biol. Crystallogr. *66*, 213-221.

Altschul, S.F., Madden, T.L., Schaffer, A.A., Zhang, J., Zhang, Z., Miller, W., and Lipman, D.J. (1997). Gapped BLAST and PSI-BLAST: a new generation of protein database search programs. Nucleic Acids Res. *25*, 3389-3402.

Biasini, M., Bienert, S., Waterhouse, A., Arnold, K., Studer, G., Schmidt, T., Kiefer, F., Gallo Cassarino, T., Bertoni, M., Bordoli, L., et al. (2014). SWISS-MODEL: modelling protein tertiary and quaternary structure using evolutionary information. Nucleic Acids Res. *42*, W252-258.

Brenner, S. (1974). The genetics of Caenorhabditis elegans. Genetics *77*, 71-94.

Bricogne, G., Blanc, E., Brandl, M., Flensburg, C., Keller, P., Paciorek, W., Roversi, P., Sharff, A., Smart, O.S., Vonrhein, C., et al. (2011). BUSTER, Version 2.10.0 Ed. Global Phasing Ltd., Cambridge, UK.

Chen, V.B., Arendall, W.B., 3rd, Headd, J.J., Keedy, D.A., Immormino, R.M., Kapral, G.J., Murray, L.W., Richardson, J.S., and Richardson, D.C. (2010). MolProbity: all-atom structure validation for macromolecular crystallography. Acta Crystallogr. D Biol. Crystallogr. *66*, 12-21.

Chevenet, F., Brun, C., Banuls, A.L., Jacq, B., and Christen, R. (2006). TreeDyn: towards dynamic graphics and annotations for analyses of trees. BMC Bioinformatics *7*, 439.

Dereeper, A., Audic, S., Claverie, J.M., and Blanc, G. (2010). BLAST-EXPLORER helps you building datasets for phylogenetic analysis. BMC Evol. Biol. *10*, 8.

Dereeper, A., Guignon, V., Blanc, G., Audic, S., Buffet, S., Chevenet, F., Dufayard, J.F., Guindon, S., Lefort, V., Lescot, M., et al. (2008). Phylogeny.fr: robust phylogenetic analysis for the non-specialist. Nucleic Acids Res. *36*, W465-469.

Edgar, R.C. (2004). MUSCLE: multiple sequence alignment with high accuracy and high throughput. Nucleic Acids Res. *32*, 1792-1797.

Emsley, P., and Cowtan, K. (2004). Coot: model-building tools for molecular graphics. Acta Crystallogr. D Biol. Crystallogr. *60*, 2126-2132.

Emsley, P., Lohkamp, B., Scott, W.G., and Cowtan, K. (2010). Features and development of Coot. Acta Crystallogr. D Biol. Crystallogr. *66*, 486-501.

Evans, P. (2006). Scaling and assessment of data quality. Acta Crystallogr. D Biol. Crystallogr. *62*, 72-82.

Evans, P.R. (2011). An introduction to data reduction: space-group determination, scaling and intensity statistics. Acta Crystallogr. D Biol. Crystallogr. *67*, 282-292.

Evans, P.R., and Murshudov, G.N. (2013). How good are my data and what is the resolution? Acta Crystallogr. D Biol. Crystallogr. *69*, 1204-1214.

Friedland, A.E., Tzur, Y.B., Esvelt, K.M., Colaiacovo, M.P., Church, G.M., and Calarco, J.A. (2013). Heritable genome editing in C. elegans via a CRISPR-Cas9 system. Nat Methods *10*, 741-743.

Guindon, S., Dufayard, J.F., Lefort, V., Anisimova, M., Hordijk, W., and Gascuel, O. (2010). New algorithms and methods to estimate maximum-likelihood phylogenies: assessing the performance of PhyML 3.0. Syst. Biol. *59*, 307-321.

Inglis, P.N., Ou, G., Leroux, M.R., and Scholey, J.M. (2007). The sensory cilia of Caenorhabditis elegans. WormBook, 1-22.

Kabsch, W. (2010). Xds. Acta Crystallogr. D Biol. Crystallogr. *66*, 125-132.

Kim, H., Ishidate, T., Ghanta, K.S., Seth, M., Conte, D., Jr., Shirayama, M., and Mello, C.C. (2014). A co-CRISPR strategy for efficient genome editing in Caenorhabditis elegans. Genetics *197*, 1069-1080.

Leslie, A.G.W., and Powell, H.R. (2007). Processing diffraction data with MOSFLM. (Netherlands: Springer).

Li, L., Li, C., Sarkar, S., Zhang, J., Witham, S., Zhang, Z., Wang, L., Smith, N., Petukh, M., and Alexov, E. (2012). DelPhi: a comprehensive suite for DelPhi software and associated resources. BMC Biophys. *5*, 9.

McCoy, A.J., Grosse-Kunstleve, R.W., Adams, P.D., Winn, M.D., Storoni, L.C., and Read, R.J. (2007). Phaser crystallographic software. J Appl Crystallogr *40*, 658-674.

McKay, S.J., Johnsen, R., Khattra, J., Asano, J., Baillie, D.L., Chan, S., Dube, N., Fang, L., Goszczynski, B., Ha, E., et al. (2003). Gene expression profiling of cells, tissues, and developmental stages of the nematode C. elegans. Cold Spring Harb. Symp. Quant. Biol. *68*, 159-169.

Murshudov, G.N., Skubak, P., Lebedev, A.A., Pannu, N.S., Steiner, R.A., Nicholls, R.A., Winn, M.D., Long, F., and Vagin, A.A. (2011). REFMAC5 for the refinement of macromolecular crystal structures. Acta Crystallogr. D Biol. Crystallogr. *67*, 355-367.

Niesen, F.H., Berglund, H., and Vedadi, M. (2007). The use of differential scanning fluorimetry to detect ligand interactions that promote protein stability. Nat. Protoc. *2*, 2212-2221.

Painter, J., and Merritt, E.A. (2006). Optimal description of a protein structure in terms of multiple groups undergoing TLS motion. Acta Crystallogr. D Biol. Crystallogr. *62*, 439-450.

Pires, D.E., Ascher, D.B., and Blundell, T.L. (2014). DUET: a server for predicting effects of mutations on protein stability using an integrated computational approach. Nucleic Acids Res. *42*, W314-319.

Reikofski, J., and Tao, B.Y. (1992). Polymerase chain reaction (PCR) techniques for site-directed mutagenesis. Biotechnol Adv *10*, 535-547.

Robert, X., and Gouet, P. (2014). Deciphering key features in protein structures with the new ENDscript server. Nucleic Acids Res. *42*, W320-324.

Sievers, F., and Higgins, D.G. (2014). Clustal Omega, accurate alignment of very large numbers of sequences. Methods Mol. Biol. *1079*, 105-116.

Winn, M.D., Ballard, C.C., Cowtan, K.D., Dodson, E.J., Emsley, P., Evans, P.R., Keegan, R.M., Krissinel, E.B., Leslie, A.G., McCoy, A., et al. (2011). Overview of the CCP4 suite and current developments. Acta Crystallogr. D Biol. Crystallogr. *67*, 235-242.

Yang, H., Guranovic, V., Dutta, S., Feng, Z., Berman, H.M., and Westbrook, J.D. (2004). Automated and accurate deposition of structures solved by X-ray diffraction to the Protein Data Bank. Acta Crystallogr. D Biol. Crystallogr. *60*, 1833-1839.

Ye, S., Dhillon, S., Ke, X., Collins, A.R., and Day, I.N. (2001). An efficient procedure for genotyping single nucleotide polymorphisms. Nucleic Acids Res. *29*, E88-88.

Zoller, M.J. (1991). New molecular biology methods for protein engineering. Curr. Opin. Biotechnol. *2*, 526-531.
