# Supplementary material for: CDKL Family Kinases Have Evolved Distinct Structural Features and Ciliary Function
Source: Cell Rep. 2018 Jan 28;22(4):885–94. doi: 10.1016/j.celrep.2017.12.083 (PMC5846859; doi:10.1016/j.celrep.2017.12.083)
Supplement: Document S2. Article plus Supplemental Information [file mmc2.pdf]

Report

Cell Reports

CDKL Family Kinases Have Evolved Distinct Structural Features and Ciliary Function

Graphical Abstract

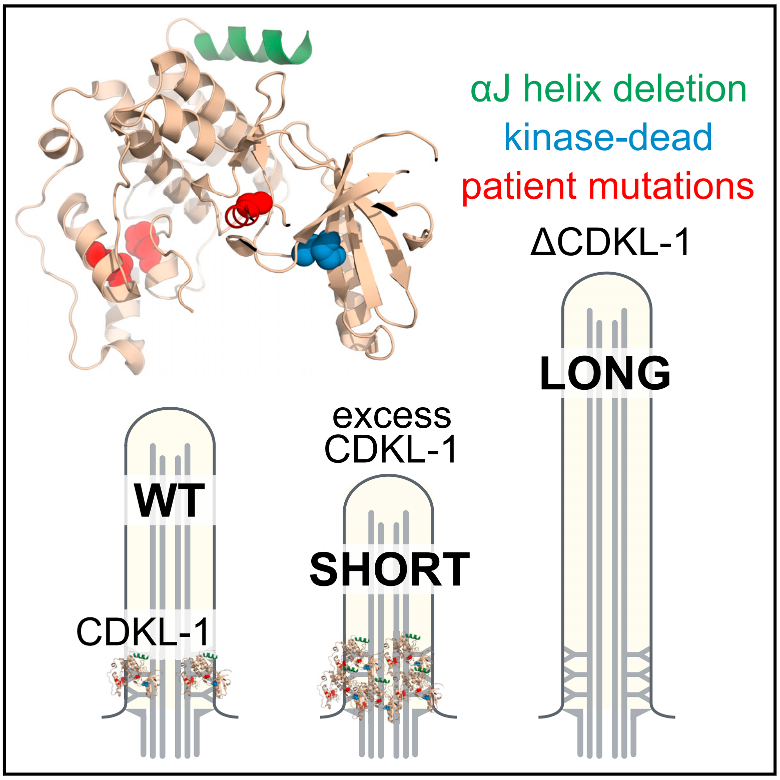

Authors

Peter Canning, Kwangjin Park,
João Gonçalves, ..., Laurence Pelletier,
Alex N. Bullock, Michel R. Leroux

Correspondence

alex.bullock@sgc.ox.ac.uk (A.N.B.),
leroux@sfu.ca (M.R.L.)

In Brief

Canning et al. reveal distinct structural features of CDKL kinases and a role for *C. elegans* CDKL-1 in cilium length control, which is lost in a kinase-dead mutant and by introducing CDKL5 disease-linked mutations. The study suggests ciliary length impairment as a potential mechanism that contributes to human neurological disorders.

Highlights

- CDKL2 and CDKL3 structures have distinct C-terminal αJ helix important for activity
- Human CDKL5 and *C. elegans* CDKL-1 localize to and have roles in cilia
- CDKL-1 cilia length control depends on kinase activity and C terminus with αJ helix
- CDKL5 disease-linked mutations cause defects in CDKL-1 cilia length regulation

Data and Software Availability

4AGU
4AAA
4BBM
3ZDU
4BGQ

Canning et al., 2018, Cell Reports *22*, 885–894
January 23, 2018 © 2018 The Authors.
https://doi.org/10.1016/j.celrep.2017.12.083

Cell Reports
Report

OPEN ACCESS
CellPress

CDKL Family Kinases Have Evolved Distinct Structural Features and Ciliary Function

Peter Canning,[1,6,9] Kwangjin Park,[2,9] João Gonçalves,[3,4] Chunmei Li,[2] Conor J. Howard,[5] Timothy D. Sharpe,[1,7] Liam J. Holt,[5,8] Laurence Pelletier,[3,4] Alex N. Bullock,[1,*] and Michel R. Leroux[2,10,*]
[1]Structural Genomics Consortium, University of Oxford, Old Road Campus, Roosevelt Drive, Oxford OX3 7DQ, UK
[2]Department of Molecular Biology and Biochemistry, and Centre for Cell Biology, Development, and Disease, Simon Fraser University, 8888 University Drive, Burnaby, BC V5A 1S6, Canada
[3]Lunenfeld-Tanenbaum Research Institute, Mount Sinai Hospital, 600 University Avenue, Toronto, ON M5G 1X5, Canada
[4]Department of Molecular Genetics, University of Toronto, Toronto, ON M5S 1A8, Canada
[5]Department of Molecular & Cell Biology, University of California, Berkeley, Berkeley, CA 94720, USA
[6]Present address: LifeArc, SBC Open Innovation Campus, Stevenage SG1 2FX, UK
[7]Present address: Biophysics Facility, Biozentrum, University of Basel, 4056 Basel, Switzerland
[8]Present address: Institute for Systems Genetics, New York University, New York, NY 10016, USA
[9]These authors contributed equally
[10]Lead Contact
*Correspondence: alex.bullock@sgc.ox.ac.uk (A.N.B.), leroux@sfu.ca (M.R.L.)
https://doi.org/10.1016/j.celrep.2017.12.083

SUMMARY

Various kinases, including a cyclin-dependent kinase (CDK) family member, regulate the growth and functions of primary cilia, which perform essential roles in signaling and development. Neurological disorders linked to CDK-Like (CDKL) proteins suggest that these underexplored kinases may have similar functions. Here, we present the crystal structures of human CDKL1, CDKL2, CDKL3, and CDKL5, revealing their evolutionary divergence from CDK and mitogen-activated protein kinases (MAPKs), including an unusual αJ helix important for CDKL2 and CDKL3 activity. *C. elegans* CDKL-1, most closely related to CDKL1–4 and localized to neuronal cilia transition zones, modulates cilium length; this depends on its kinase activity and αJ helix-containing C terminus. Human CDKL5, linked to Rett syndrome, also localizes to cilia, and it impairs ciliogenesis when overexpressed. CDKL5 patient mutations modeled in CDKL-1 cause localization and/or cilium length defects. Together, our studies establish a disease model system suggesting cilium length defects as a pathomechanism for neurological disorders, including epilepsy.

INTRODUCTION

Primary (non-motile) cilia are organelles found in most eukaryotic cells, including neurons, that perform essential roles in human sensory physiology, cell signaling, and development (May-Simera and Kelley, 2012; Mukhopadhyay and Rohatgi, 2014; Oh and Katsanis, 2012). They are anchored by a basal body that templates the growth of the microtubule-based axoneme (Carvalho-Santos et al., 2011). The first ciliary subcompartment formed is the transition zone (TZ), which harbors Y-shaped links that make axoneme-to-membrane connections. The TZ functions as a membrane diffusion barrier that maintains the correct ciliary composition of the compartment, which is enriched in signaling proteins (Reiter et al., 2012; Williams et al., 2011). The axoneme is built by an intraflagellar transport (IFT) system that uses kinesin/dynein motors and adaptors that mobilize cargo into and out of cilia (Blacque and Sanders, 2014; Sung and Leroux, 2013). Disruption of basal body, TZ, and IFT proteins results in ciliopathies that exhibit a broad spectrum of clinical ailments (Reiter and Leroux, 2017).

The length of cilia must be tightly regulated to ensure optimal functions in their given cell types (Keeling et al., 2016). IFT plays a central role in cilium length control (Broekhuis et al., 2013). Length regulation further involves depolymerizing kinesins and tubulin modifications that influence microtubule stability (Liang et al., 2016). Kinases represent another category of proteins that influence cilium formation as well as length (Avasthi and Marshall, 2012; Cao et al., 2009).

Kinases implicated in cilium length control largely belong to two groups. One is the NIMA-related kinase (Nek) family, which includes mammalian Nek1 and Nek8, and CNK2, CNK4, and CNK11 from *Chlamydomonas* (Bradley and Quarmby, 2005; Hilton et al., 2013; Lin et al., 2015; Meng and Pan, 2016; Shalom et al., 2008; Sohara et al., 2008). CMGC kinases (CDKs, mitogen-activated protein kinases [MAPK], glycogen synthase kinases [GSK], and CDK-like kinases [CLK]) represent the other group, with mammalian Cyclin-Dependent Kinase 5 (CDK5) and Cell Cycle-Related Kinase (CCRK) influencing cilium length (Husson et al., 2016; Phirke et al., 2011; Tam et al., 2007; Yang et al., 2013). Several members from a specific branch of CMGC kinases (Figure 1A) also regulate cilium length, namely ICK, MAK, MOK, GSK3β, and CDK-Like 5 (CDKL5) (Bengs et al., 2005; Berman et al., 2003; Broekhuis et al., 2014; Burghoorn et al., 2007; Hu et al., 2015; Omori et al., 2010; Tam et al., 2013; Wilson and Lefebvre, 2004). Notably, human CDKL5 belongs to a family of CDKL kinases encompassing CDKL1, CDKL2, CDKL3, and CDKL4.

This is an open access article under the CC BY license (http://creativecommons.org/licenses/by/4.0/).

OPEN
ACCESS
CellPress

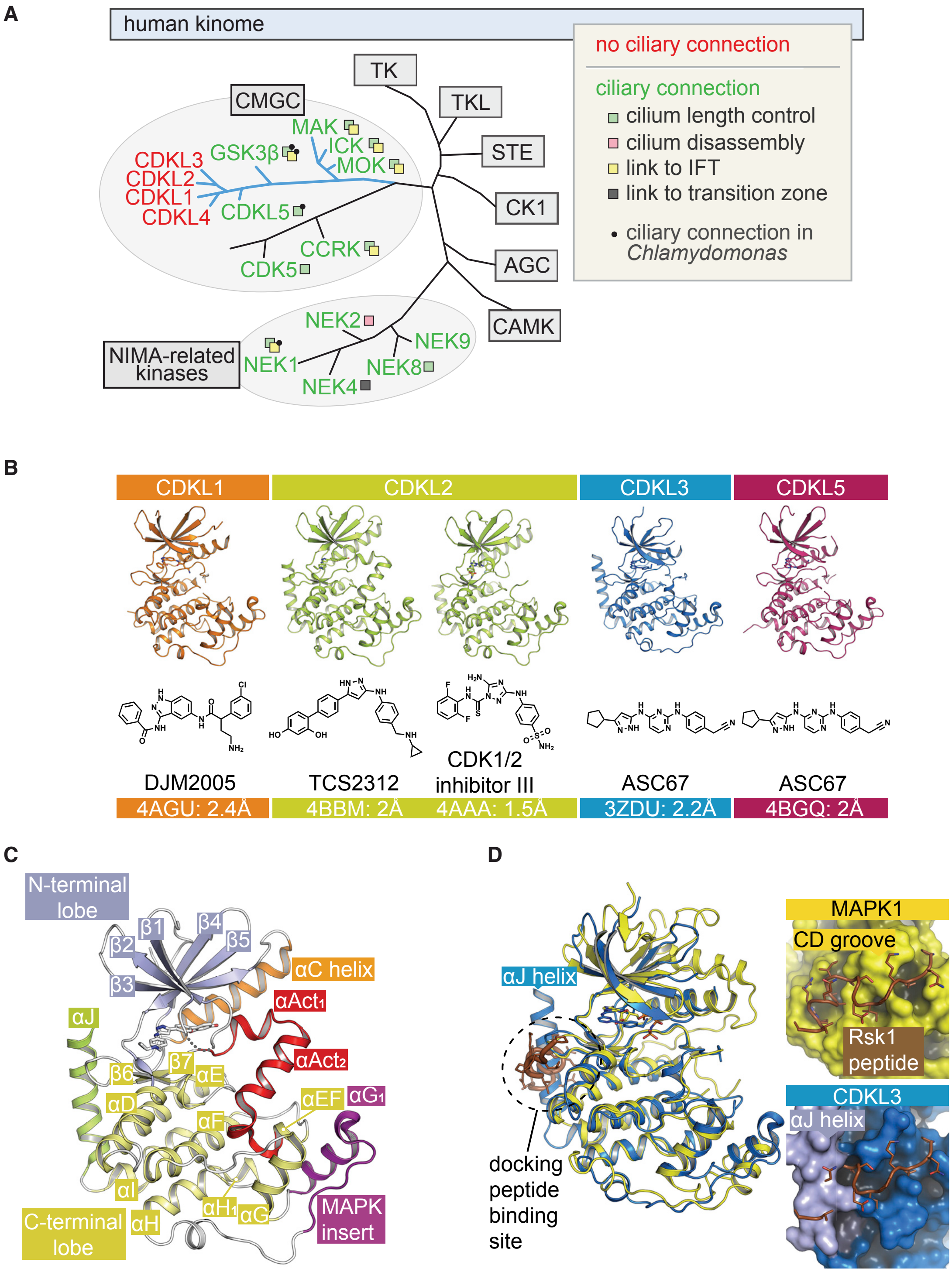

Figure 1. Unusual Structural Features of the CDKL Kinase Domains

(A) Phylogenetic distribution of NIMA-related kinases (Neks) or CMGC group kinases with known ciliary functions (green), including cilium length control, disassembly, association with intraflagellar transport (IFT), and TZ localization. A branch of the CMGC group (blue) includes several kinases (mammalian MAK, ICK, and MOK; *Chlamydomonas* GSK3β and CDKL5) that regulate cilium length, several of which also influence IFT. Human CDKL1, CDKL2, CDKL3, and CDKL4 (red) have no previously known links to cilia.

(B) Crystal structures of human CDKL1, CDKL2, CDKL3, and CDKL5 with the indicated inhibitors.

(C) Structural features of the CDKL2-TCS2312 complex, the most complete/ordered CDKL structure.

(D) Structural comparison of CDKL3 with MAPK1 (ERK2; PDB ID: 3TEI). Inset panels show Rsk1 docking peptide bound to MAPK1 and superimposed onto CDKL3.

CDKL proteins share a high degree of sequence similarity with CDKs, and they contain the MAPK TXY phosphorylation motif needed for activity (Yee et al., 2003). They have putative cyclin-binding domains, but there is no evidence of interaction with cyclins. However, no CDKL family member has been structurally characterized. Moreover, aside from CDKL5, little is known about CDKL protein function. Disrupting CDKL5 causes Rett syndrome, a neurodevelopmental disorder that exhibits early-onset seizures, mental retardation, and autism (Castrén et al., 2011; Kilstrup-Nielsen et al., 2012). Consistent with having neuronal functions, CDKL5 facilitates dendritic spine and excitatory synapse formation, possibly via AKT/GSK-3β signaling (Fuchs et al., 2014). Intriguingly, *Chlamydomonas* CDKL5 orthologs regulate cilium length (Hu et al., 2015; Tam et al., 2013); however, such a function in metazoans has not been reported. Knockdown of zebrafish CDKL1 causes Hedgehog signaling defects (Hsu et al., 2011) that hint at a ciliary role, but the localization and function of the protein remain unknown.

Here we present the crystal structures of CDKL1, CDKL2, CDKL3, and CDKL5, solved in various active and inactive kinase domain conformations. The structures reveal an unusual αJ helix important for CDKL2 and CDKL3 function and further structural changes to putative substrate docking sites that support the divergence of CDKL kinases from the CDK and MAPK families. We show that, unlike other TZ proteins, the sole *C. elegans* CDKL protein family member (CDKL-1), which localizes to the ciliary TZ (Li et al., 2016), does not regulate the diffusion barrier. Instead, CDKL-1 regulates cilium length, in a kinase activity- and αJ helix C-terminal region-dependent manner. We present evidence that human CDKL5 is a ciliary protein with a potential role in ciliogenesis, and we show that *C. elegans* CDKL-1 variants modeling CDKL5 human patient mutations exhibit cilium length defects, with or without loss of TZ localization. Together, our structure-function studies provide the first high-resolution structural insights into the CDKL protein family; reveal that CDKL proteins may share a common function in cilium length control; and show that CDKL5-associated Rett syndrome may stem, at least in part, from ciliary dysfunction.

RESULTS AND DISCUSSION

The CDKL Kinase Domain Contains an Unusual αJ Helix

CDKL family proteins contain a conserved N-terminal kinase domain and variable C termini (Figure S1). The kinase domains of CDKL1, CDKL2, CDKL3, and CDKL5 were crystallized using a phosphomimetic Asp-X-Glu (DXE) substitution and in the presence of identified inhibitors (Table S1). Their structures were solved at resolutions from 1.5 to 2.4 Å (Figure 1B; Table S2). The prototypical structure, exemplified by CDKL2, conformed to the classic bilobal kinase architecture (Figure 1C). Of note, the C-terminal lobe diverged at the two MAPK family docking sites, suggesting that CDKL family members mediate alternative protein interactions. First, the MAPK insert folded into a single large αG_1 helix and loop, whereas MAPKs typically contain two shorter helices for recruiting the substrate Asp-Glu-Phe (DEF) motif (Figure 1D). The typical MAPK insert also packed against the αG helix, orientated roughly perpendicular to the equivalent motif in CDKL2 (Figure 1D). Second, the CDKL2 kinase domain was extended at the C terminus by an unusual αJ helix that occupied a site equivalent to the MAPK common docking (CD) groove and, thus, occluded part of the recruitment site for the D(ocking) motifs of MAPK substrates (Figure 1D). Interestingly, the packing and orientation of the αJ helix was distinct from the C-terminal extensions of other kinases, such as PAK1, CDK2, BUB1, and NEK1 (Figure S2). Hence, it will be interesting to explore the proteomics of these kinases, with or without the αJ helix region, to uncover any specific protein interactions. Other divergent docking sites, such as the MAPK insert, further indicate the status of CDKL proteins as a distinct kinase family, for which key regulatory and substrate partners remain to be identified.

Small-Molecule Inhibitors Can Bind to Both Active and Inactive CDKL Conformations

The CDKL structures all contain broad spectrum ATP-competitive inhibitors that bind to the kinase hinge region via 3 hydrogen bonds (Figures S3A–S3C). CDKL2 co-crystallized in an inactive αC-out conformation with two inhibitors, CDK1/2 Inhibitor III and CHK1 inhibitor TCS2312 (Figure 2A). Their binding was stabilized by a collapsed P loop conformation that establishes potential allosteric sites for inhibitor design (Figures S3A and S3B). While an inactive conformation appears necessary for TCS2312 binding, the CDK1/2 Inhibitor III is compatible with an active kinase conformation, suggesting that the inactive configuration of CDKL2 is not driven solely by inhibitor interactions.

By contrast, the CDKL1, CDKL3, and CDKL5 structures displayed characteristics of active kinases. Inhibitor DJM2005 showed a preference for CDKL1, and it formed an extra H-bond to the catalytic loop residue N131 (Figure S3C). Inhibitor ASC67 was designed as an affinity reagent (Statsuk et al., 2008) and was among the top screening hits for all CDKLs tested (Table S1). Its nitrile H bonded with the catalytic lysines of CDKL3 and CDKL5 (Figure S3C). Catalytic site interactions may help support the active conformation of these kinases. Together, the structures and inhibitor screens suggest potential for generating isoform- as well as conformation-selective inhibitors of the CDKL family.

CDKL Structures Reveal Conformational Changes during Kinase Activation

Superposition of CDKL1 and CDKL2 highlighted conformational changes needed for kinase activation (Figure 2A). In CDKL2, the ATP-binding pocket was sterically occluded by the P loop and activation segment (αAct_1 and αAct_2). This forced the αC helix to swing outward, breaking the salt bridge between the catalytic lysine (K33) and glutamate (E51, αC). The CDKL1 structure showed an ~11-Å shift in αC position that restored the salt bridge (Figure 2A). In this active configuration, the P loop also formed the expected β1-β2 hairpin. However, the substrate-binding pocket was disrupted by a disordered activation segment, showing incomplete activation by the phosphomimetic DXE motif (Figure 2A).

The αJ Helix Is Critical for the Kinase Activity of CDKL2 and CDKL3

To date, no activating partners are known for the CDKL family. Structural comparisons with the αC PSTAIRE motif of CDK2

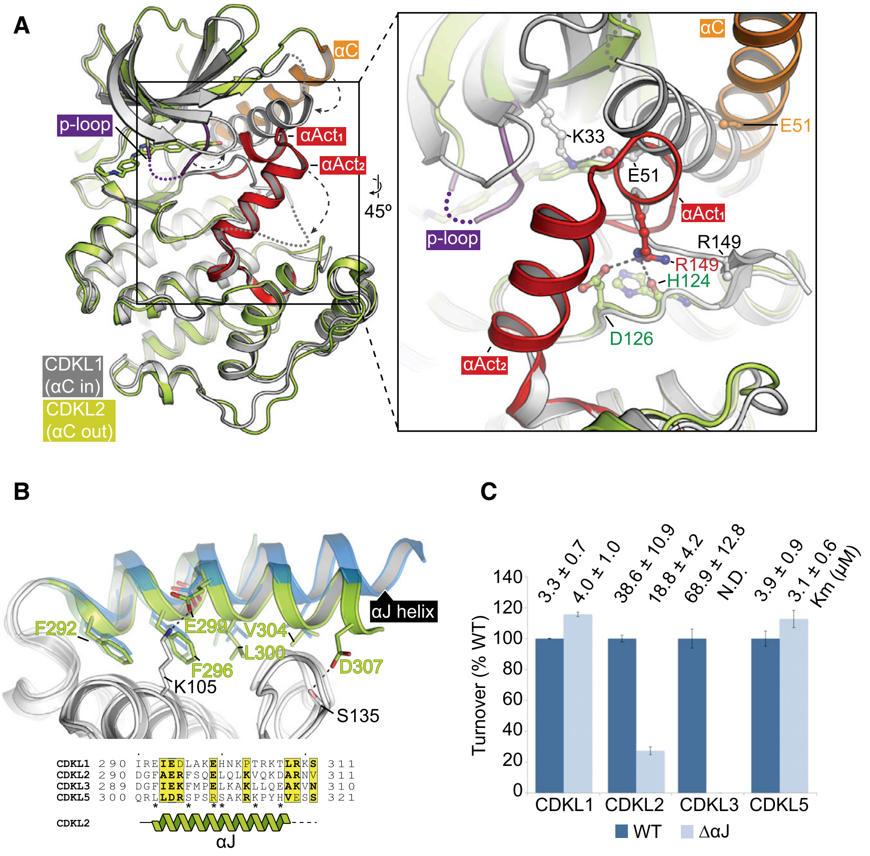

Figure 2. Structural Features Determining CDKL Activation

(A) Superposition of CDKL1 (αC-in, gray) and CDKL2 (αC-out, light green). Inset highlights changes in the positions of the αC and activation segments.

(B) Sequence and structural comparisons show the αJ conservation in CDKL2 (green) and CDKL3 (blue). An asterisk denotes αJ interactions with the kinase domain.

(C) A radiometric *in vitro* kinase assay reveals that the αJ region is critical for CDKL2 and CDKL3 activities but dispensable for CDKL1 and CDKL5. K_m values are shown for the Ime2 peptide substrate. N.D. denotes not determined for CDKL3(ΔαJ) due to diminished catalytic activity.

reveal several bulky substitutions in CDKLs that likely preclude binding to cyclins, although novel interaction partners cannot be excluded (Figure S3D). MAPKs establish a similar αC interaction intramolecularly through their C-terminal helix αL16. However, we found no evidence for an equivalent structural element in CDKLs. Instead, CDKL2 and CDKL3 showed an unusual amphipathic helix, αJ, while the constructs for CDKL1 and CDKL5 were truncated and lacked this region (Figure 2B).

To determine the functional relevance of the αJ, we expressed the wild-type (WT) proteins in yeast, and we performed *in vitro* kinase assays (Figure 2C). Proline-directed activity was observed against an Ime2 peptide substrate (RPRSPGARR), consistent with other CMGC kinases. Turnover was low (<10 phosphorylations/min) for all CDKLs, perhaps reflecting a requirement for activating partners. Notably, deleting the αJ region reduced the activities of CDKL2 and CDKL3, whereas CDKL1 and CDKL5 were largely unchanged (Figure 2C). These results are consistent with the observed structures, and they further show the importance of the unprecedented αJ helix for CDKL2 and CDKL3 function.

C. elegans CDKL-1 Localizes to the Ciliary Transition Zone but Appears Dispensable for Cilium Gate Function

To investigate the collective function of CDKL proteins, we chose to study the sole member encoded by *C. elegans*, CDKL-1. This kinase is most closely related to mammalian CDKL1–4 and more distantly related to CDKL5 (Figure 3A). The *cdkl-1* gene is specifically expressed in ciliated sensory neurons (Figure S4A), likely due to the presence of an X-box motif found in the promoters of most ciliary genes (Blacque et al., 2005). The CDKL-1 protein localizes to ciliary TZs in head (amphid) and tail (phasmid) neurons (Li et al., 2016) (Figure S4B), whose ascribed functions are as a membrane diffusion barrier, or ciliary gate, that maintains the protein composition of the organelle (Reiter et al., 2012).

Given its TZ localization, we wondered if CDKL-1 plays a role in ciliary gating. To test this, we probed if two proteins normally found at the periciliary membrane, namely TRAM-1a and RPI-2, could inappropriately enter the ciliary compartment in a strain lacking CDKL-1. TRAM-1a and RPI-2 entered cilia in most TZ mutants tested (Huang et al., 2011; Jensen et al., 2015; Williams et al., 2011). In contrast, they remained at the periciliary membrane in the *cdkl-1* mutant, as in the WT control (Figures S4C and S4D). We also queried for the leakage of ARL-13 from its normal localization (ciliary middle segment) to the periciliary membrane, as seen in various TZ mutants (Cevik et al., 2013; Li et al., 2016). ARL-13 ciliary localization was unchanged in the *cdkl-1* mutant, similar to WT (Figure S4E).

Although the *cdkl-1*(*tm4182*) mutant used above contains a large out-of-frame deletion (850 bp) of exons 3–7 and is likely null, we also made another *null* (*nx132*) mutant using CRISPR-Cas9, which has a 5-bp deletion in the first coding exon, causing an early stop (Figure 3B). TRAM-1a, RPI-2, and ARL-13 localization remained unperturbed in this *cdkl-1* mutant (Figures S4C–S4E), providing further evidence that CDKL-1 performs a non-canonical function unrelated to ciliary gating at the TZ.

CDKL-1 Modulates Cilium Length

Given that MAK, ICK, MOK, GSK3β, and CDKL5 regulate cilium length (Figure 1A), we hypothesized that *C. elegans* CDKL-1 plays a similar role. To test this, we expressed GFP-tagged IFT-20 in the bi-ciliated ADL neuron to measure cilium length accurately (Mohan et al., 2013). IFT-20::GFP, which marks the basal body and axoneme, was introduced into WT and *cdkl-1*

OPEN
ACCESS
CellPress

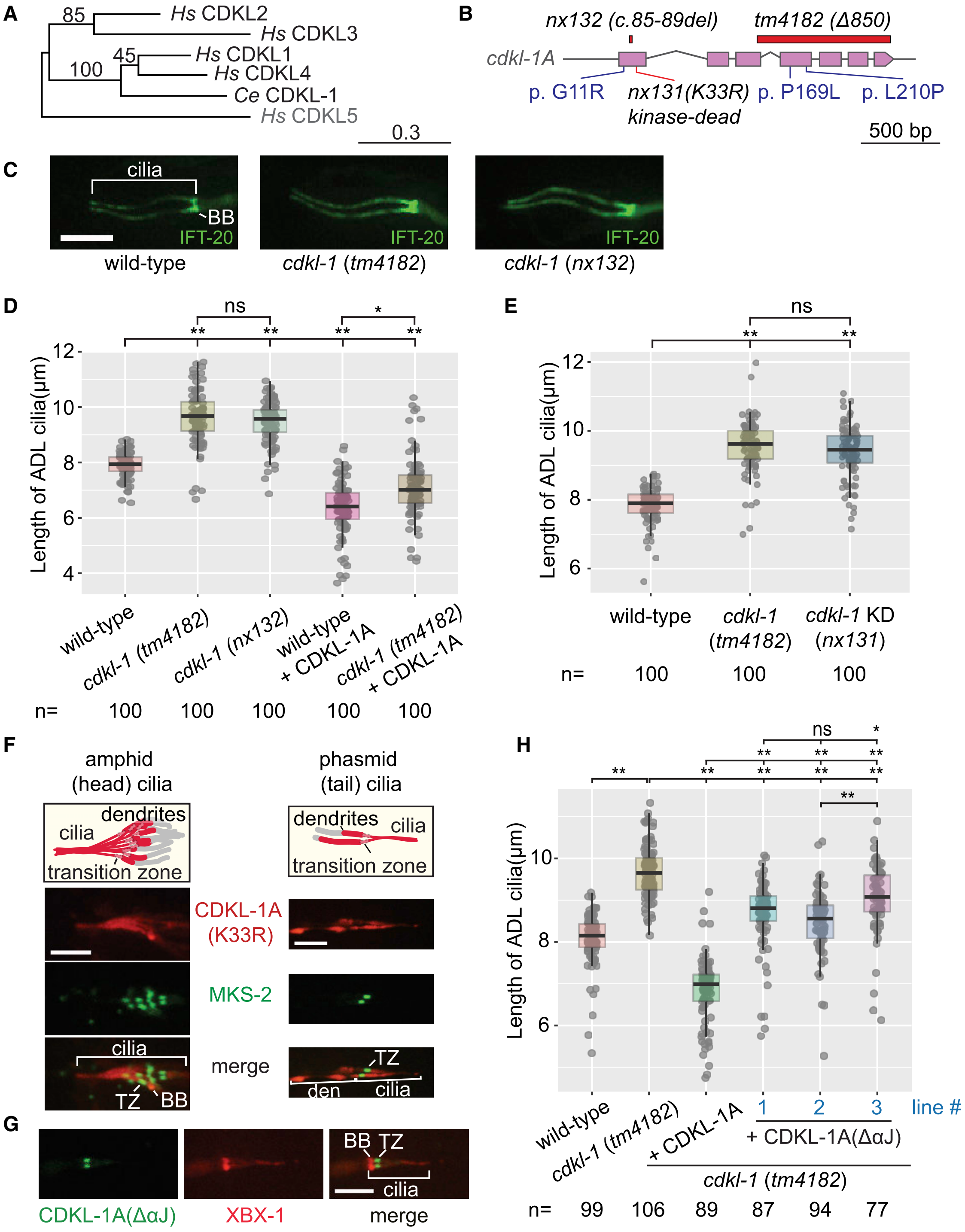

Figure 3. *C. elegans* CDKL-1 Requires Its Kinase Activity and C-Terminal Region (Including αJ Helix) to Regulate Cilium Length

(A) Phylogenetic relationship between *H. sapiens* (*Hs*) and *C. elegans* (*Ce*) CDKL proteins.

(B) Gene structure of *cdkl-1A*, highlighting the deletion or missense mutants analyzed.

(C) Representative images of the GFP-tagged IFT-20 marker expressed specifically in ADL neurons (L4 larvae), used to measure the length of cilia in WT and *cdkl-1* mutants (*tm4182* and *nx132*). ADL doublet cilia are longer in mutants than WT. BB, basal body. Scale bar, 4 μm.

(D) ADL cilia lengths (L4 larvae) of WT and *cdkl-1* mutants with/without expression of WT CDKL-1A construct. Each dot represents one cilium. Kruskal-Wallis test (Dunn Kruskal-Wallis multiple comparison [Holm-Sidak method]) was used for significance in (D), (E), and (H). *p < 0.01 and **p < 0.001; ns, not significant.

(E) ADL cilia length in WT, *cdkl-1* null (*tm4182*), and *cdkl-1* kinase-dead (KD) (*nx131*) mutant L4 larvae. Dot, one cilium. **p < 0.001; ns, not significant.

(legend continued on next page)

mutant (*tm4182* and *nx132*) animals. Whereas the median length of WT ADL cilia was 8.0 μm, *cdkl-1* mutant cilia were 9.6 μm, or ~20% longer (Figures 3C and 3D). We confirmed that the long ADL cilia correctly penetrated amphid channels, which are formed by sheath and socket cells (Figure S4F).

We sought to rescue the cilium length defect of *cdkl-1* mutants by expressing WT *cdkl-1*, but we found that this shortens ciliary length by ~11%, to 7.1 μm (Figures 3D and S4G). Similarly, overexpressing *cdkl-1* in a WT background reduced ciliary length. Hence, loss or increased levels of CDKL-1 activity led to longer or shorter cilia, suggesting that the correct level of CDKL-1 kinase activity is needed to maintain correct cilium length.

Phylogenetically, CDKL proteins are split into two ancestral groups, namely CDKL1–4 and CDKL5. Our results provide the first evidence for a CDKL1–4-related protein in cilium length regulation. As *C. elegans* only encodes one CDKL protein, related to CDKL1–4 (as in *Drosophila*), our results suggest that both CDKL5 and CDKL1–4 proteins may share functions in cilium length regulation. Vertebrates/mammals encode CDKL5 and CDKL1/2/3/4 proteins. This may indicate divergent functions for the different members, which may be cilium dependent and/or independent, including cell proliferation and tumorigenesis, neuronal differentiation, cognition, and learning (Gomi et al., 2010; Kilstrup-Nielsen et al., 2012; Liu et al., 2010; Sun et al., 2012).

CDKL-1 Kinase Activity Is Required for Cilium Length Control and Transition Zone Localization

We examined if CDKL-1 kinase activity is needed to regulate cilium length. Using CRISPR-Cas9, we generated a *cdkl-1* kinase-dead mutant (*nx131*) by converting a conserved lysine (K) in the ATP-binding site to arginine (R) (Figures 3B and S5D). Like the *cdkl-1* (*tm4182*) null mutant, the kinase-dead mutant exhibited cilia ~20% longer than WT (Figure 3E). In addition, the CDKL-1(K33R) protein no longer concentrated at the TZ; it dispersed in the dendrite and ciliary axoneme (Figure 3F). Thus, CDKL-1 kinase activity is critical for regulating cilium length and proper TZ localization of the protein. Whether CDKL-1 phosphorylates itself and/or target(s) within the TZ, which promotes TZ localization, is unclear. Future studies aimed at uncovering targets (perhaps IFT proteins), and interaction partners, will shed light on the mechanism by which CDKL-1 regulates cilium length.

C-terminal αJ Helix Region of CDKL-1 Is Needed to Maintain Ciliary Length

Since our structure-function studies uncovered the αJ helix as crucial for CDKL2/CDKL3 kinase activity, we probed its role in *C. elegans*. An mNeonGreen-tagged CDKL-1A(ΔαJ) protein variant was expressed, and it was found to localize correctly to the TZ, suggesting that this region does not overtly affect protein stability (Figure 3G). However, unlike WT CDKL-1A, the CDKL-1A(ΔαJ) protein only partially rescued the cilium length defect in a *cdkl-1* mutant, suggesting that the αJ helix region is important for cilium length control (Figures 3H and S4H).

CDKL-1 Variants Carrying Human CDKL5 Pathogenic Mutations Disrupt Ciliary Length Control

CDKL5 mutations, most occurring in the kinase domain, are linked to neurological disorders, including epilepsy, atypical Rett syndrome, and autism. We sought to model such CDKL5 mutations using *C. elegans* CDKL-1, but first we wanted to reveal a functional connection between human CDKL5 and cilia to ensure the relevance of our studies. To this end, we found that GFP-tagged human CDKL5 localizes at the basal body, as well as ciliary tip in ciliated RPE-1 cells (Figures 4A and 4B). In non-ciliated cells, GFP-CDKL5 localized to the centrosome in a microtubule-independent manner (Figure S5A). Compared to serum-starved WT RPE-1 cells, cells expressing GFP-CDKL5 exhibited compromised ciliogenesis (Figures 4C and S5C). This negative effect of GFP-CDKL5 overexpression was rescued by RNAi-mediated depletion of CDKL5 (Figures 4C, S5B, and S5C). Our findings confirm the first association between vertebrate/mammalian CDKL5 and cilia.

We chose to model three pathogenic CDKL5 missense mutations (G20R, P180L, and L220P) present in patients with epileptic encephalopathy, severe mental retardation, developmental delay, or spasms (Kilstrup-Nielsen et al., 2012). The residues are conserved in all human CDKL proteins and *C. elegans* CDKL-1 (Figures S1D and S5D). The corresponding mutations (G11R, P169L, and L210P) were introduced in *C. elegans* CDKL-1 to model their influence on TZ localization (Figure 3B). Strikingly, CDKL-1 proteins harboring the G11R or L210P mutations no longer concentrated at the TZ (Figure 4D). CDKL-1(G11R) was primarily dispersed in the ciliary axoneme, whereas CDKL-1(L210P) showed weak localization to cilia and periciliary membrane. In contrast, CDKL-1(P169L) localized to the TZ, similar to WT.

Next, we assessed the functionality of each CDKL-1 variant by testing their effect on ADL cilium length when expressed in a *cdkl-1* mutant. Relative to WT CDKL-1, expression of each variant gave statistically longer cilia in almost all lines, with the phenotypic severity ranked L210P > G11R > P169L (Figure 4E). Substituting proline in the L210P variant likely disrupted the αG helix, potentially causing protein misfolding (Figure S5E) and, thus, dysfunction due to mislocalization and/or loss of kinase activity. The G11R missense mutation was positioned at the start of the P loop GXGXXG motif, where a bulky substitution was predicted to preclude ATP binding (Figure S5E). This may have reduced CDKL-1 catalytic activity

(F) The kinase-dead variant CDKL-1A(K33R)::tdTomato no longer concentrates at the TZ (marked by MKS-2::GFP); it mislocalizes to dendrites (den) and cilia in amphid and phasmid neurons. BB, basal body. Scale bar, 4 μm.

(G) CDKL-1A(ΔαJ)::mNeonGreen protein predominantly accumulates at the TZ in cilia. The BB and ciliary axoneme are marked by XBX-1::tdTomato. Scale bar, 4 μm.

(H) ADL cilia lengths (L4 larvae) measured in WT, *cdkl-1* null (*tm4182*), and *cdkl-1* null (*tm4182*) expressing CDKL-1A or CDKL-1A(ΔαJ). Dot, one cilium. *$p < 0.05$ and **$p < 0.001$; ns, not significant.

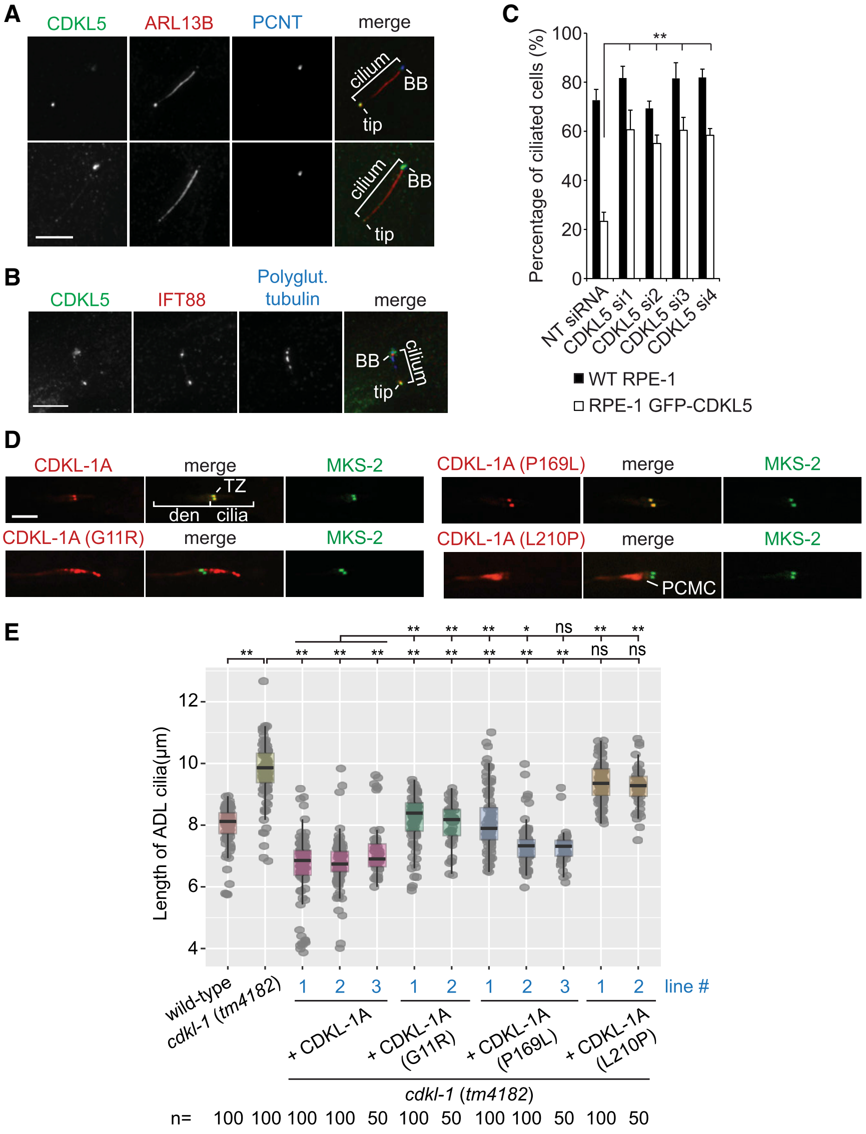

Figure 4. Human CDKL5 Localizes to Cilium and Affects Ciliogenesis When Overexpressed in RPE-1 Cells and Mutations Modeled in *C. elegans* CDKL-1 Disrupt Localization and/or Cilium Length Regulation

(A) Immunofluorescence analysis of serum-starved hTERT RPE-1 cells expressing GFP-CDKL5 stained with antibodies against GFP, ARL13B (cilium marker), and PCNT (centrosome marker). The top and bottom panels are representative images showing different levels of GFP-CDKL5 localization to the BB and cilium tip. Scale bar, 5 μm.

(B) Serum-starved hTERT RPE-1 GFP-CDKL5 cells were stained with antibodies against GFP, IFT88, and polyglutamylated tubulin (centriole/BB and axoneme marker). Scale bar, 5 μm.

(C) Bar graph shows mean percentage of ciliated cells (n > 300 cells per sample, 3 independent experiments) in the serum-starved populations (72 hr) of hTERT RPE-1 and hTERT RPE-1 GFP-CDKL5 cells transfected with the indicated siRNAs. Error bars indicate SD. **p < 0.01 (Student's two-tailed t test).

(D) WT CDKL-1A and the P169L variants localize to the TZ, whereas the G11R mutant localizes along the length of cilia, and the L210P mutant accumulates in dendrites (den) and is weakly present at the periciliary membrane compartment (PCMC). MKS-2 is a TZ marker. Scale bar, 4 μm.

(E) ADL cilia lengths (L4 larvae) of WT and strains expressing CDKL-1 variants. Expressing WT CDKL-1A leads to short cilia. Similar levels of length reduction are not seen upon expression of the G11R and L210P variants, or lines 1 and 2 of the P169L variant (line 3 is not significant), suggesting functional defects with these variants. Dot is one cilium. Significance (p value) was calculated by Dunn Kruskal-Wallis multiple comparison (Holm-Sidak adjustment). *p < 0.01 and **p < 0.001; ns, not significant.

and, similar to the kinase-dead mutant (Figure 3F), induced mislocalization. The weaker P169L phenotype may have reflected a partial loss of function (2 of 3 lines showed different ciliary lengths), with the protein remaining TZ localized (Figures 4D and 4E).

Together, our modeling of CDKL5 patient mutations using *C. elegans* CDKL-1 suggests that cilium length may, at least in part, cause the observed neurological phenotypes present in patients with CDKL5 mutations. Dissecting the relationship among kinase activity, TZ localization, and mechanism of ciliary length regulation will necessitate further studies.

Model for CDKL Protein Regulation of Cilium Length

One specific branch of the kinome (Figure 1A) is enriched with proteins having ciliary functions (Avasthi and Marshall, 2012). ICK, MOK, and MAK localize to cilia and negatively regulate their length (Broekhuis et al., 2014; Chaya et al., 2014; Omori et al., 2010). Notably, IFT components accumulate inside cilia when ICK or MAK are lost. Moreover, ICK phosphorylates a subunit of kinesin-2 and affects IFT speeds, consistent with the link between cilium length control and IFT. *Chlamydomonas* orthologs of CDKL5 and GSK3β (LF5/FLS1 and GSK3) also localize to, and control the length of, cilia (Hu et al., 2015; Tam et al., 2013; Wilson and Lefebvre, 2004). *Chlamydomonas* CDKL5 (LF5) localizes at the ciliary base and its disruption lengthens cilia (Tam et al., 2013), similar to loss of *C. elegans* CDKL-1. Interestingly, *Chlamydomonas* CDKL5 moves to ciliary tips in long-flagella (*lf*) mutants, suggesting a link to length control. Specifically, CDKL5 is influenced by LF3, whose loss causes IFT protein accumulation at ciliary tips (Tam et al., 2003). This is of interest, since human CDKL5 localizes at the base, and also tip, of cilia (Figures 4A, 4B, and 5). Hence, CDKL5 (and perhaps other CDKL proteins) influence IFT at the base, or tip, of cilia to regulate cilium length. Consistent

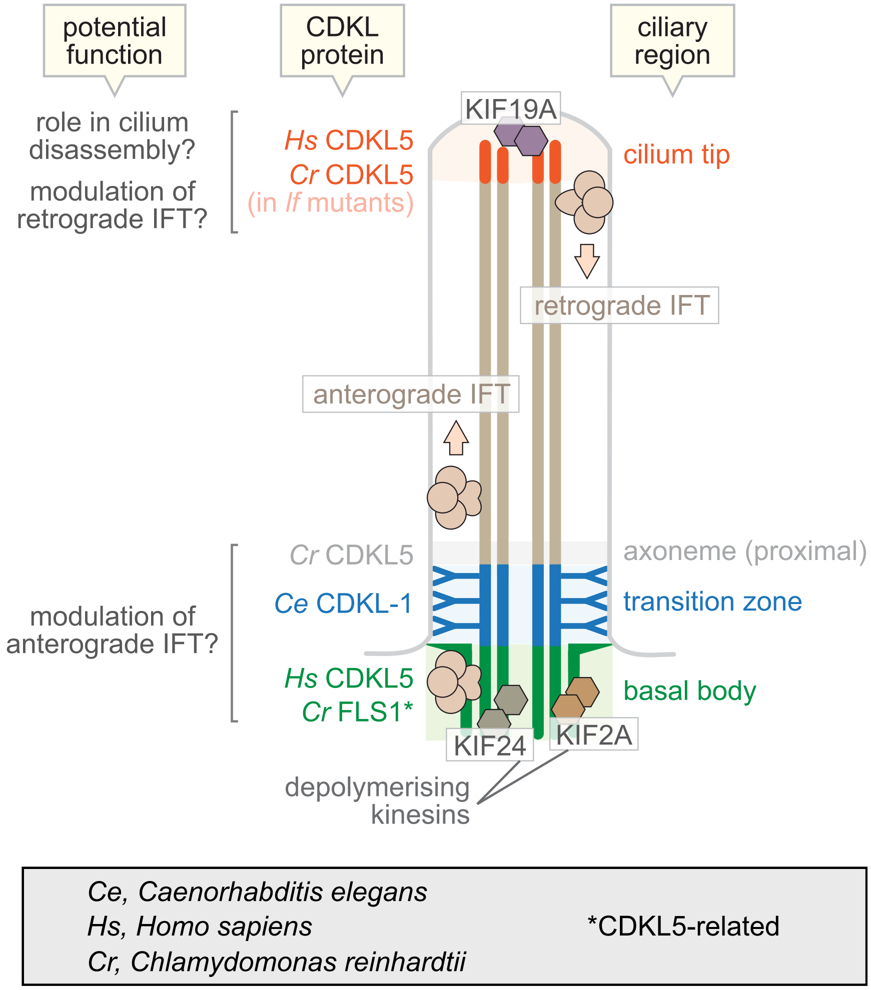

Figure 5. Model for CDKL Protein Ciliary Localization and Potential Roles in Cilium Length Regulation

CDKL proteins localize to the base and/or tip of cilia, and they likely influence IFT (anterograde/retrograde) machinery and/or depolymerizing kinesins to regulate cilium growth and disassembly, respectively, and thus control cilium length.

with such a role, overexpressing CDKL5 impairs cilium formation in RPE-1 cells.

Interestingly, *C. elegans* CDKL-1 localizes to the TZ, whereas human CDKL5 and another CDKL5-related *Chlamydomonas* protein (FLS1) are at the basal body (Hu et al., 2015) (Figure 5). This might reflect different functions of CDKL1–4 or CDKL5 family members, although the *Chlamydomonas* CDKL5 is present just distal to the TZ. Hence, CDKL proteins exist in three regions at the ciliary base—basal body, TZ, and distal to the TZ (Figure 5). At any of these locations, CDKL proteins may be well positioned to interact with the IFT machinery. Such a transient interaction has been described for the TZ protein B9D1 (Zhao and Malicki, 2011). Another possibility, not mutually exclusive, is that CDKL5 and perhaps other CDKL proteins act via depolymerizing kinesin(s). This is reported for *Chlamydomonas* FLS1, which phosphorylates a kinesin-13 member in flagella (Hu et al., 2015). In mammals, two cilium base-localized kinesin-13 proteins, KIF2A and KIF24, regulate cilium disassembly/length control (Kim et al., 2015; Kobayashi et al., 2011; Miyamoto et al., 2015) (Figure 5). CDKL proteins may also influence another depolymerizing kinesin, KIF19A, found at the ciliary tip (Niwa et al., 2012). In sum, CDKL proteins may control cilium structure and length through the IFT machinery and/or depolymerizing kinesins, at the ciliary base and/or tip (Figure 5). Understanding their ciliary roles may be relevant to human neurological disorders, including epilepsy.

EXPERIMENTAL PROCEDURES

Cloning of Human CDKL Kinase Domains

The CDKL1 kinase domain sequence (UniProt: Q00532; residues 1–303; T159D, Y161E, and N301D) was cloned into the bacterial vector pNIC-CTHF. The CDKL2 (UniProt: Q92772; residues 1–308; T159D and Y161E), CDKL3 (UniProt: Q8IVW4; residues 1–324; T158D and Y160E), and CDKL5 (UniProt: O76039; residues 1–303; T169D and Y171E) domains were cloned into the baculoviral vector pFB-LIC-Bse. More details are in the Supplemental Experimental Procedures.

In Vitro Kinase Assays

Kinase activity was profiled in 50 mM HEPES (pH 7.5), 50 mM NaCl, 10 mM $MgCl_2$, 500 μM ATP, 83.3 μg/mL BSA, 0.833% glycerol, and 0.2 μCi [γ-^{32}P]ATP. Minimal kinase concentrations sufficient for signal (0.05–10 μM) were determined empirically. See also the Supplemental Experimental Procedures.

Construction of *C. elegans* cdkl-1A Translational Fusions

For the various *cdkl-1A* (Y42A5A.4A) translational fusions, all introns and exons of *cdkl-1A*, including its 1.8-kb 5′ UTR, were amplified from the genomic DNA, and they were fused to GFP or tdTomato with the *unc-54* 3′ UTR or *cdkl-1* 3′ UTR without any fluorescent proteins tags. The mutations found in CDKL5 human patients (G20R, P180L, and L220P) or kinase-dead (CDKL5 K42R) mutations were introduced into the *cdkl-1* gene by replacing the corresponding residues, and they were fused to tdTomato to assess localization. Plasmids encoding tdTomato-tagged or untagged CDKL-1A variants harboring CDKL5 mutations, and those encoding CDKL-1A(ΔαJ) (residues 1–286) with/without mNeonGreen, were prepared with the CloneJET PCR Cloning Kit.

C. elegans Strains and Imaging

All nematode strains (Table S3) were maintained at 20°C and imaged using a spinning-disc confocal microscope, as described in Li et al. (2016).

ADL Ciliary Length Measurement and Statistical Analysis

ADL cilia lengths (from basal body to tip) of L4 larvae were measured and plotted using Dot and Boxplots in R software. The distribution of each dataset was determined by Shapiro-Wilk test. The statistical significance (p value) was calculated by the Dunn's Kruskal-Wallis Multiple Comparisons with Holm-Sidak adjustment.

Human CDKL5 Constructs

A Gateway entry clone with the coding sequence of human CDKL5 (NM_003159.2) was obtained from the LTRI plasmid repository, and it was used to clone CDKL5 in fusion with GFP in the pcDNA5-FRT/TO-GFP vector. The GFP-CDKL5 fusion was subsequently subcloned into the lentiviral vector pHR-SIN-SFFV to generate the pHR-SIN-SFFV-GFP-CDKL5 plasmid.

Mammalian RNAi and Statistical Analyses

To silence CDKL5, hTERT RPE-1 and hTERT-RPE-1 GFP-CDKL5 cells (5×10^4 cells seeded in 12-well plates) were transfected with 40 nM (final concentration) of 4 small interfering RNAs (siRNAs) targeting CDKL5 obtained from Dharmacon (ON-TARGET plus) using Lipofectamine RNAiMAX transfection reagent (Invitrogen). The Luciferase GL2 Duplex non-targeting siRNA from Dharmacon was used as a negative control. The cells were transfected and serum-starved for 72 hr to induce the formation of primary cilia. The p values are from two-tailed unpaired Student's t tests.

DATA AND SOFTWARE AVAILABILITY

The accession numbers for the structures reported in this paper are PDB: 4AGU, 4AAA, 4BBM, 3ZDU, and 4BGQ.

SUPPLEMENTAL INFORMATION

Supplemental Information includes Supplemental Experimental Procedures, five figures, and three tables and can be found with this article online at https://doi.org/10.1016/j.celrep.2017.12.083.

ACKNOWLEDGMENTS

We thank Diamond Light Source for beamtime (proposals mx442, mx6391, and mx8421) and the staff of beamlines I02, I03, I04-1, and I24 for their help with data collection. The Structural Genomics Consortium (SGC) is a registered charity (number 1097737) funded by AbbVie, Bayer Pharma AG, Boehringer Ingelheim, Canada Foundation for Innovation, Eshelman Institute for Innovation, Genome Canada, Innovative Medicines Initiative (EU/EFPIA) (ULTRA-DD grant 115766), Janssen, MSD, Merck KGaA, Novartis Pharma AG, Ontario Ministry of Economic Development and Innovation, Pfizer, São Paulo Research Foundation-FAPESP, and Takeda and Wellcome (106169/ZZ14/Z). ASC67 and DJM2005 were provided by Kevan Shokat. Additional funding was provided by the Canadian Institutes of Health Research (CIHR; grants MOP142243 and MOP82870 to M.R.L. and MOP142492 to L.P.) and Bowes Research Fellowship to L.J.H. L.P. holds a Canada Research Chair in Centrosome Biogenesis and function. K.P. is the recipient of a Vanier Canada Graduate Scholarship. M.R.L. acknowledges a senior investigator award from the Michael Smith Foundation for Health Research.

AUTHOR CONTRIBUTIONS

P.C., K.P., C.L., L.J.H., A.N.B., J.G., L.P., and M.R.L. designed the research. P.C., K.P., J.G., C.L., C.J.H., and T.D.S. performed the experiments and data analysis. L.J.H., A.N.B., L.P., and M.R.L. supervised the project. P.C., K.P., C.L., C.J.H., A.N.B., J.G., and M.R.L. made figures, and P.C., K.P., L.J.H., A.N.B., J.G., and M.R.L. wrote the manuscript.

DECLARATION OF INTERESTS

The authors declare no competing interests.

Received: March 21, 2017
Revised: November 7, 2017
Accepted: December 22, 2017
Published: January 23, 2018

REFERENCES

Avasthi, P., and Marshall, W.F. (2012). Stages of ciliogenesis and regulation of ciliary length. Differentiation *83*, S30–S42.

Bengs, F., Scholz, A., Kuhn, D., and Wiese, M. (2005). LmxMPK9, a mitogen-activated protein kinase homologue affects flagellar length in Leishmania mexicana. Mol. Microbiol. *55*, 1606–1615.

Berman, S.A., Wilson, N.F., Haas, N.A., and Lefebvre, P.A. (2003). A novel MAP kinase regulates flagellar length in Chlamydomonas. Curr. Biol. *13*, 1145–1149.

Blacque, O.E., and Sanders, A.A. (2014). Compartments within a compartment: what *C. elegans* can tell us about ciliary subdomain composition, biogenesis, function, and disease. Organogenesis *10*, 126–137.

Blacque, O.E., Perens, E.A., Boroevich, K.A., Inglis, P.N., Li, C., Warner, A., Khattra, J., Holt, R.A., Ou, G., Mah, A.K., et al. (2005). Functional genomics of the cilium, a sensory organelle. Curr. Biol. *15*, 935–941.

Bradley, B.A., and Quarmby, L.M. (2005). A NIMA-related kinase, Cnk2p, regulates both flagellar length and cell size in Chlamydomonas. J. Cell Sci. *118*, 3317–3326.

Broekhuis, J.R., Leong, W.Y., and Jansen, G. (2013). Regulation of cilium length and intraflagellar transport. Int. Rev. Cell Mol. Biol. *303*, 101–138.

Broekhuis, J.R., Verhey, K.J., and Jansen, G. (2014). Regulation of cilium length and intraflagellar transport by the RCK-kinases ICK and MOK in renal epithelial cells. PLoS ONE *9*, e108470.

Burghoorn, J., Dekkers, M.P., Rademakers, S., de Jong, T., Willemsen, R., and Jansen, G. (2007). Mutation of the MAP kinase DYF-5 affects docking and undocking of kinesin-2 motors and reduces their speed in the cilia of Caenorhabditis elegans. Proc. Natl. Acad. Sci. USA *104*, 7157–7162.

Cao, M., Li, G., and Pan, J. (2009). Regulation of cilia assembly, disassembly, and length by protein phosphorylation. Methods Cell Biol. *94*, 333–346.

Carvalho-Santos, Z., Azimzadeh, J., Pereira-Leal, J.B., and Bettencourt-Dias, M. (2011). Evolution: Tracing the origins of centrioles, cilia, and flagella. J. Cell Biol. *194*, 165–175.

Castrén, M., Gaily, E., Tengström, C., Lähdetie, J., Archer, H., and Ala-Mello, S. (2011). Epilepsy caused by CDKL5 mutations. Eur. J. Paediatr. Neurol. *15*, 65–69.

Cevik, S., Sanders, A.A., Van Wijk, E., Boldt, K., Clarke, L., van Reeuwijk, J., Hori, Y., Horn, N., Hetterschijt, L., Wdowicz, A., et al. (2013). Active transport and diffusion barriers restrict Joubert Syndrome-associated ARL13B/ARL-13 to an Inv-like ciliary membrane subdomain. PLoS Genet. *9*, e1003977.

Chaya, T., Omori, Y., Kuwahara, R., and Furukawa, T. (2014). ICK is essential for cell type-specific ciliogenesis and the regulation of ciliary transport. EMBO J. *33*, 1227–1242.

Fuchs, C., Trazzi, S., Torricella, R., Viggiano, R., De Franceschi, M., Amendola, E., Gross, C., Calzà, L., Bartesaghi, R., and Ciani, E. (2014). Loss of CDKL5 impairs survival and dendritic growth of newborn neurons by altering AKT/GSK-3β signaling. Neurobiol. Dis. *70*, 53–68.

Gomi, H., Sassa, T., Thompson, R.F., and Itohara, S. (2010). Involvement of cyclin-dependent kinase-like 2 in cognitive function required for contextual and spatial learning in mice. Front. Behav. Neurosci. *4*, 17.

Hilton, L.K., Gunawardane, K., Kim, J.W., Schwarz, M.C., and Quarmby, L.M. (2013). The kinases LF4 and CNK2 control ciliary length by feedback regulation of assembly and disassembly rates. Curr. Biol. *23*, 2208–2214.

Hsu, L.S., Liang, C.J., Tseng, C.Y., Yeh, C.W., and Tsai, J.N. (2011). Zebrafish cyclin-dependent protein kinase-like 1 (zcdkl1): identification and functional characterization. Int. J. Mol. Sci. *12*, 3606–3617.

Hu, Z., Liang, Y., He, W., and Pan, J. (2015). Cilia disassembly with two distinct phases of regulation. Cell Rep. *10*, 1803–1810.

Huang, L., Szymanska, K., Jensen, V.L., Janecke, A.R., Innes, A.M., Davis, E.E., Frosk, P., Li, C., Willer, J.R., Chodirker, B.N., et al. (2011). TMEM237 is mutated in individuals with a Joubert syndrome related disorder and expands the role of the TMEM family at the ciliary transition zone. Am. J. Hum. Genet. *89*, 713–730.

Husson, H., Moreno, S., Smith, L.A., Smith, M.M., Russo, R.J., Pitstick, R., Sergeev, M., Ledbetter, S.R., Bukanov, N.O., Lane, M., et al. (2016). Reduction of ciliary length through pharmacologic or genetic inhibition of CDK5 attenuates polycystic kidney disease in a model of nephronophthisis. Hum. Mol. Genet. *25*, 2245–2255.

Jensen, V.L., Li, C., Bowie, R.V., Clarke, L., Mohan, S., Blacque, O.E., and Leroux, M.R. (2015). Formation of the transition zone by Mks5/Rpgrip1L establishes a ciliary zone of exclusion (CIZE) that compartmentalises ciliary signalling proteins and controls PIP2 ciliary abundance. EMBO J. *34*, 2537–2556.

Keeling, J., Tsiokas, L., and Maskey, D. (2016). Cellular mechanisms of ciliary length control. Cells *5*, E6.

Kilstrup-Nielsen, C., Rusconi, L., La Montanara, P., Ciceri, D., Bergo, A., Bedogni, F., and Landsberger, N. (2012). What we know and would like to know about CDKL5 and its involvement in epileptic encephalopathy. Neural Plast. *2012*, 728267.

Kim, S., Lee, K., Choi, J.H., Ringstad, N., and Dynlacht, B.D. (2015). Nek2 activation of Kif24 ensures cilium disassembly during the cell cycle. Nat. Commun. *6*, 8087.

Kobayashi, T., Tsang, W.Y., Li, J., Lane, W., and Dynlacht, B.D. (2011). Centriolar kinesin Kif24 interacts with CP110 to remodel microtubules and regulate ciliogenesis. Cell *145*, 914–925.

Li, C., Jensen, V.L., Park, K., Kennedy, J., Garcia-Gonzalo, F.R., Romani, M., De Mori, R., Bruel, A.L., Gaillard, D., Doray, B., et al. (2016). MKS5 and CEP290 dependent assembly pathway of the ciliary transition zone. PLoS Biol. *14*, e1002416.

Liang, Y., Meng, D., Zhu, B., and Pan, J. (2016). Mechanism of ciliary disassembly. Cell. Mol. Life Sci. *73*, 1787–1802.

Lin, H., Zhang, Z., Guo, S., Chen, F., Kessler, J.M., Wang, Y.M., and Dutcher, S.K. (2015). A NIMA-related kinase suppresses the Flagellar instability associated with the loss of multiple axonemal structures. PLoS Genet. *11*, e1005508.

Liu, Z., Xu, D., Zhao, Y., and Zheng, J. (2010). Non-syndromic mild mental retardation candidate gene CDKL3 regulates neuronal morphogenesis. Neurobiol. Dis. *39*, 242–251.

May-Simera, H.L., and Kelley, M.W. (2012). Cilia, Wnt signaling, and the cytoskeleton. Cilia *1*, 7.

Meng, D., and Pan, J. (2016). A NIMA-related kinase, CNK4, regulates ciliary stability and length. Mol. Biol. Cell *27*, 838–847.

Miyamoto, T., Hosoba, K., Ochiai, H., Royba, E., Izumi, H., Sakuma, T., Yamamoto, T., Dynlacht, B.D., and Matsuura, S. (2015). The microtubule-depolymerizing activity of a mitotic kinesin protein KIF2A drives primary cilia disassembly coupled with cell proliferation. Cell Rep. *10*, 664–673.

Mohan, S., Timbers, T.A., Kennedy, J., Blacque, O.E., and Leroux, M.R. (2013). Striated rootlet and nonfilamentous forms of rootletin maintain ciliary function. Curr. Biol. *23*, 2016–2022.

Mukhopadhyay, S., and Rohatgi, R. (2014). G-protein-coupled receptors, Hedgehog signaling and primary cilia. Semin. Cell Dev. Biol. *33*, 63–72.

Niwa, S., Nakajima, K., Miki, H., Minato, Y., Wang, D., and Hirokawa, N. (2012). KIF19A is a microtubule-depolymerizing kinesin for ciliary length control. Dev. Cell *23*, 1167–1175.

Oh, E.C., and Katsanis, N. (2012). Cilia in vertebrate development and disease. Development *139*, 443–448.

Omori, Y., Chaya, T., Katoh, K., Kajimura, N., Sato, S., Muraoka, K., Ueno, S., Koyasu, T., Kondo, M., and Furukawa, T. (2010). Negative regulation of ciliary length by ciliary male germ cell-associated kinase (Mak) is required for retinal photoreceptor survival. Proc. Natl. Acad. Sci. USA *107*, 22671–22676.

Phirke, P., Efimenko, E., Mohan, S., Burghoorn, J., Crona, F., Bakhoum, M.W., Trieb, M., Schuske, K., Jorgensen, E.M., Piasecki, B.P., et al. (2011). Transcriptional profiling of C. elegans DAF-19 uncovers a ciliary base-associated protein and a CDK/CCRK/LF2p-related kinase required for intraflagellar transport. Dev. Biol. *357*, 235–247.

Reiter, J.F., and Leroux, M.R. (2017). Genes and molecular pathways underpinning ciliopathies. Nat. Rev. Mol. Cell Biol. *18*, 533–547.

Reiter, J.F., Blacque, O.E., and Leroux, M.R. (2012). The base of the cilium: roles for transition fibres and the transition zone in ciliary formation, maintenance and compartmentalization. EMBO Rep. *13*, 608–618.

Shalom, O., Shalva, N., Altschuler, Y., and Motro, B. (2008). The mammalian Nek1 kinase is involved in primary cilium formation. FEBS Lett. *582*, 1465–1470.

Sohara, E., Luo, Y., Zhang, J., Manning, D.K., Beier, D.R., and Zhou, J. (2008). Nek8 regulates the expression and localization of polycystin-1 and polycystin-2. J. Am. Soc. Nephrol. *19*, 469–476.

Statsuk, A.V., Maly, D.J., Seeliger, M.A., Fabian, M.A., Biggs, W.H., 3rd, Lockhart, D.J., Zarrinkar, P.P., Kuriyan, J., and Shokat, K.M. (2008). Tuning a three-component reaction for trapping kinase substrate complexes. J. Am. Chem. Soc. *130*, 17568–17574.

Sun, W., Yao, L., Jiang, B., Shao, H., Zhao, Y., and Wang, Q. (2012). A role for Cdkl1 in the development of gastric cancer. Acta Oncol. *51*, 790–796.

Sung, C.H., and Leroux, M.R. (2013). The roles of evolutionarily conserved functional modules in cilia-related trafficking. Nat. Cell Biol. *15*, 1387–1397.

Tam, L.W., Dentler, W.L., and Lefebvre, P.A. (2003). Defective flagellar assembly and length regulation in LF3 null mutants in Chlamydomonas. J. Cell Biol. *163*, 597–607.

Tam, L.W., Wilson, N.F., and Lefebvre, P.A. (2007). A CDK-related kinase regulates the length and assembly of flagella in Chlamydomonas. J. Cell Biol. *176*, 819–829.

Tam, L.W., Ranum, P.T., and Lefebvre, P.A. (2013). CDKL5 regulates flagellar length and localizes to the base of the flagella in Chlamydomonas. Mol. Biol. Cell *24*, 588–600.

Williams, C.L., Li, C., Kida, K., Inglis, P.N., Mohan, S., Semenec, L., Bialas, N.J., Stupay, R.M., Chen, N., Blacque, O.E., et al. (2011). MKS and NPHP modules cooperate to establish basal body/transition zone membrane associations and ciliary gate function during ciliogenesis. J. Cell Biol. *192*, 1023–1041.

Wilson, N.F., and Lefebvre, P.A. (2004). Regulation of flagellar assembly by glycogen synthase kinase 3 in Chlamydomonas reinhardtii. Eukaryot. Cell *3*, 1307–1319.

Yang, Y., Roine, N., and Mäkelä, T.P. (2013). CCRK depletion inhibits glioblastoma cell proliferation in a cilium-dependent manner. EMBO Rep. *14*, 741–747.

Yee, K.W., Moore, S.J., Midmer, M., Zanke, B.W., Tong, F., Hedley, D., and Minden, M.D. (2003). NKIAMRE, a novel conserved CDC2-related kinase with features of both mitogen-activated protein kinases and cyclin-dependent kinases. Biochem. Biophys. Res. Commun. *308*, 784–792.

Zhao, C., and Malicki, J. (2011). Nephrocystins and MKS proteins interact with IFT particle and facilitate transport of selected ciliary cargos. EMBO J. *30*, 2532–2544.

Cell Reports, Volume *22*

Supplemental Information

CDKL Family Kinases Have Evolved

Distinct Structural Features and Ciliary Function

Peter Canning, Kwangjin Park, João Gonçalves, Chunmei Li, Conor J. Howard, Timothy D. Sharpe, Liam J. Holt, Laurence Pelletier, Alex N. Bullock, and Michel R. Leroux

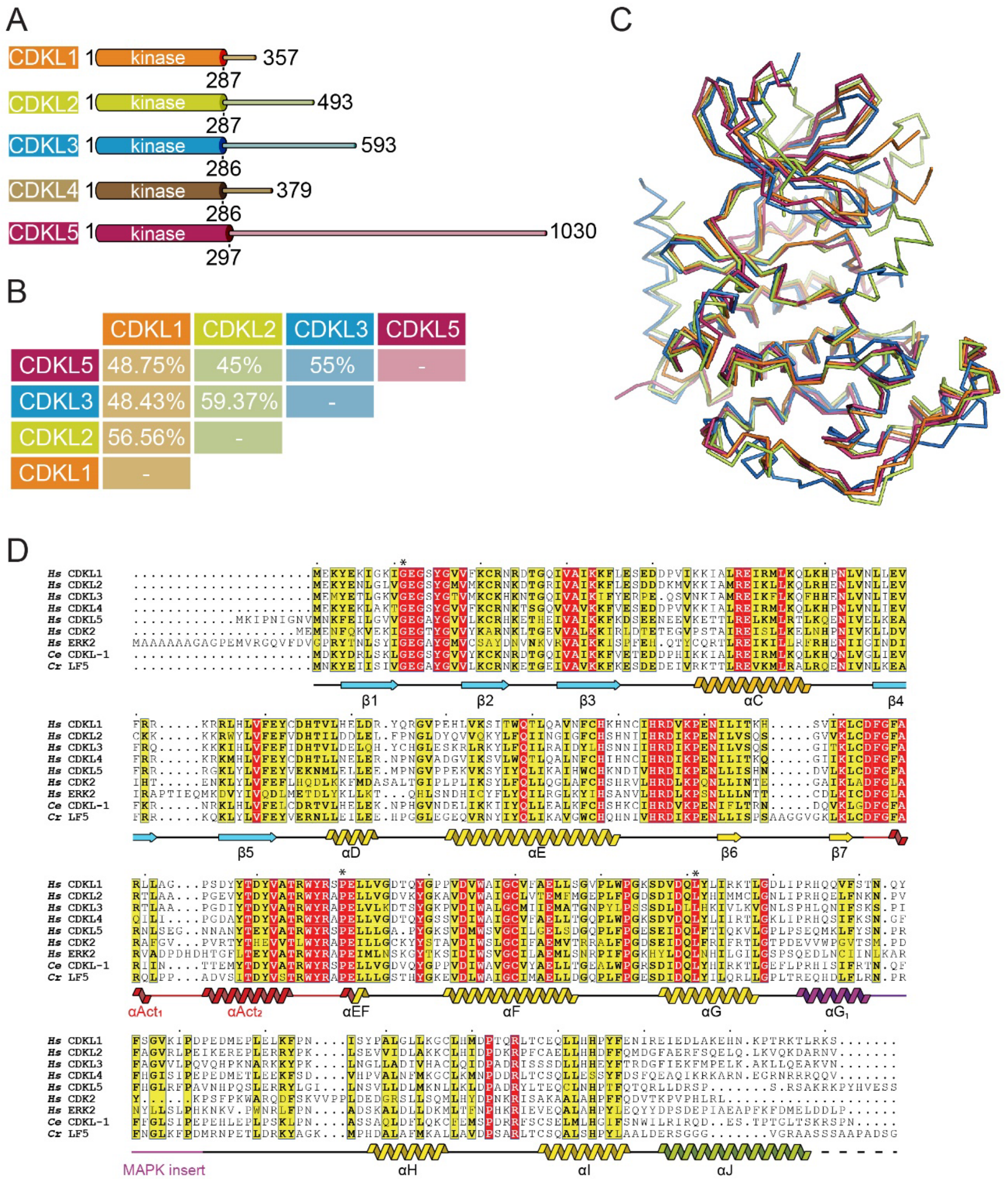

Figure S1. Comparison of the CDKL kinase domain structures. Related to Figure 1.
(**A**) The predicted kinase domains (UNIPROT annotations) of human CDKL1-5 are shown relative to the size of the full-length proteins
(**B**) Pairwise sequence identities between the kinase domains of the four CDKL family members for which structures were solved.
(**C**) Superposition of Cα backbones of all four CDKL kinase domains colored as panel (**B**).
(**D**) Sequence alignment of the kinase domains of *H. sapiens* (*Hs*) CDKL1-5, CDK2, ERK2 (MAPK1), *C. elegans* (*Ce*) CDKL-1 and *Chlamydomonas reinhardtii* (*Cr*) LF5. The secondary structure elements from the CDKL2 complex with TCS2312 are shown. Asterisks mark the sites of disease-causing mutations analyzed in this study.

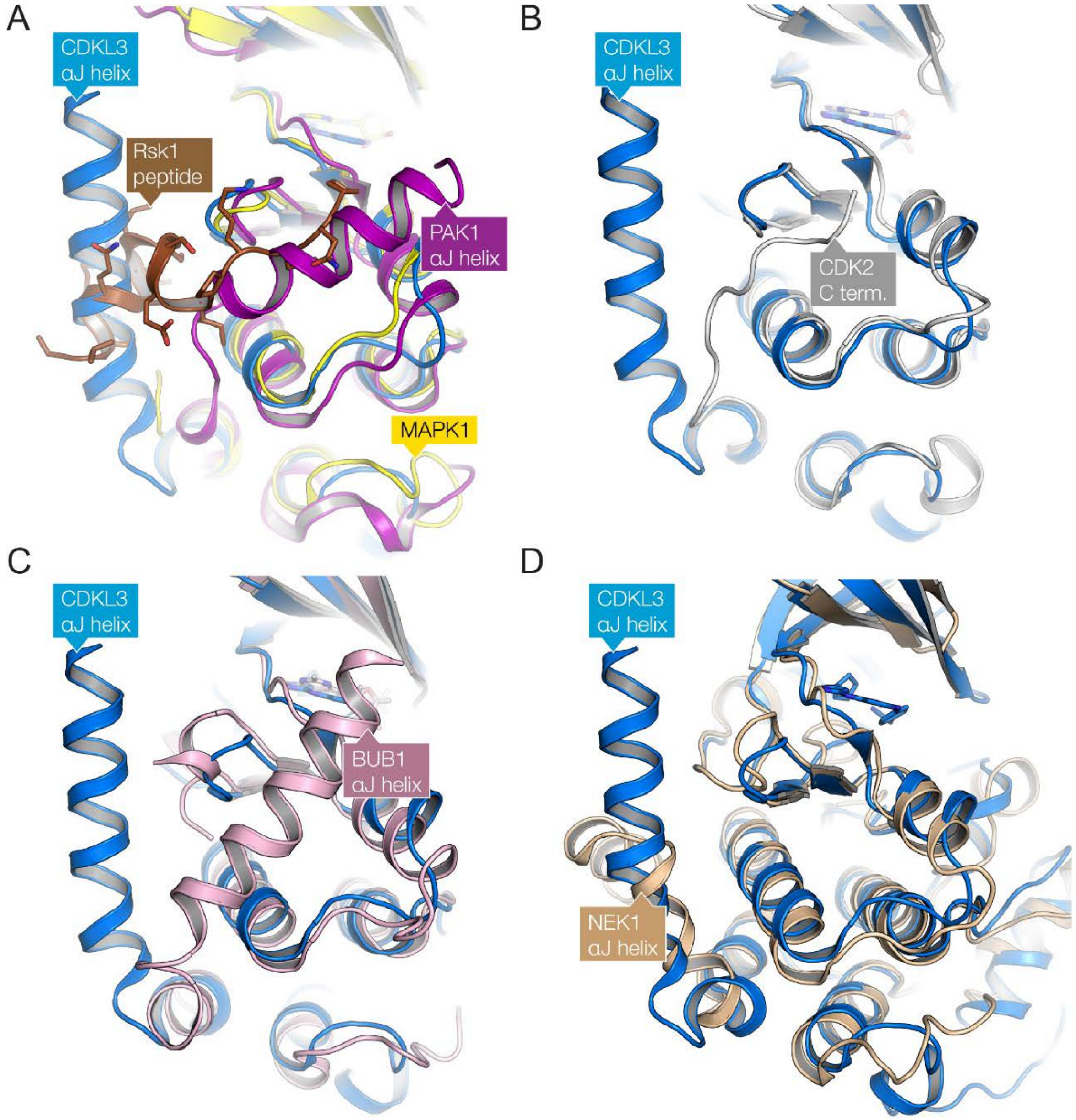

Figure S2. The CDKL αJ helix occupies an unusual position compared to the C-terminal extensions in other protein kinases. Related to Figure 1.
(**A**) Comparison of the MAPK1 (ERK2) peptide docking site with other kinases. MAPK1 (yellow, PDB ID: 3TEI), CDKL3 (blue) and PAK1 (magenta, PDB ID: 1F3M) are shown as cartoons and superimposed, and the MAPK1-bound Rsk1 peptide is shown as a cartoon with stick side-chains. PAK1 is displayed as a representative member of the STE family kinases.
(**B**) Superposition of the CDKL3 (blue) and CDK2 (gray, PDB ID: 1QMZ) structures. The CDK2 C-terminus occupies a site similar to the Rsk1 peptide bound to MAPK1, whereas this site is largely vacated in the CDKL3 structure by the unusual positioning of the αJ helix.
(**C**) Superposition of the CDKL3 (blue) and BUB1 (pink, PDB ID: 4QPM) structures. The BUB1 C-terminus occupies a similar position to PAK1 above, but displays a longer helix. BUB1 is an example of a remote kinase falling outside the major kinase subfamilies.
(**D**) Superposition of the CDKL3 (blue) and NEK1 (bronze, PDB ID: 4APC) structures. The NEK1 C-terminus folds more similarly to CDKL3 at the N-terminus of the αJ helix, but then turns perpendicularly to follow the path of the αE helix. NEK kinases also form a remote kinase clade falling outside the major kinase subfamilies.

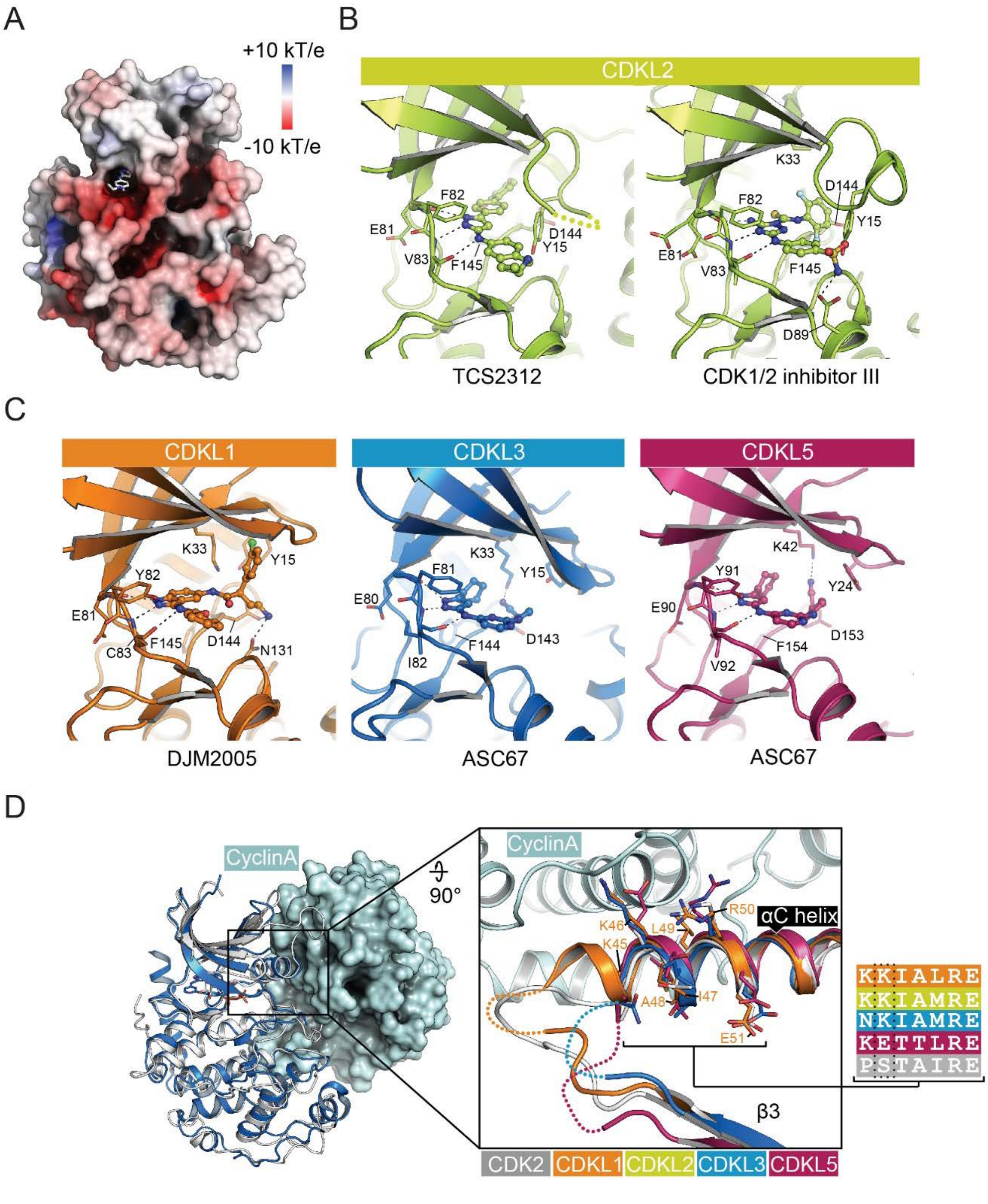

Figure S3. Inhibitor interactions and partial conservation of the CDK2 PSTAIRE motif in the CDKL family structures. Related to Figure 2.
(**A**) Surface representation of CDKL2 (PDB ID: 4BBM) colored by electrostatic potential.
(**B**) The ATP-binding pocket of CDKL2 with the two bound inhibitors. The Kinase domain is shown as a cartoon and the inhibitors as ball-and-stick representations. Key residues involved in inhibitor binding are shown as sticks and labeled.
(**C**) The ATP binding pockets of CDKL1, 3 and 5, shown as described for (**B**).
(**D**) Comparison of the CDKL3 structure (blue) with the structure of CDK2 (gray) bound to Cyclin A (pale blue surface representation) (PDB ID: 1FIN). The inset panel shows the superposition of the αC region of CDK2 and

indicated CDKLs; the alignment panel shows the sequences of these proteins across this region. Side chains are shown as sticks and numbering shown for residues in CDKL1. The sidechains marked in bold have been modeled as they were not built during crystal structure refinement due to poor electron density.

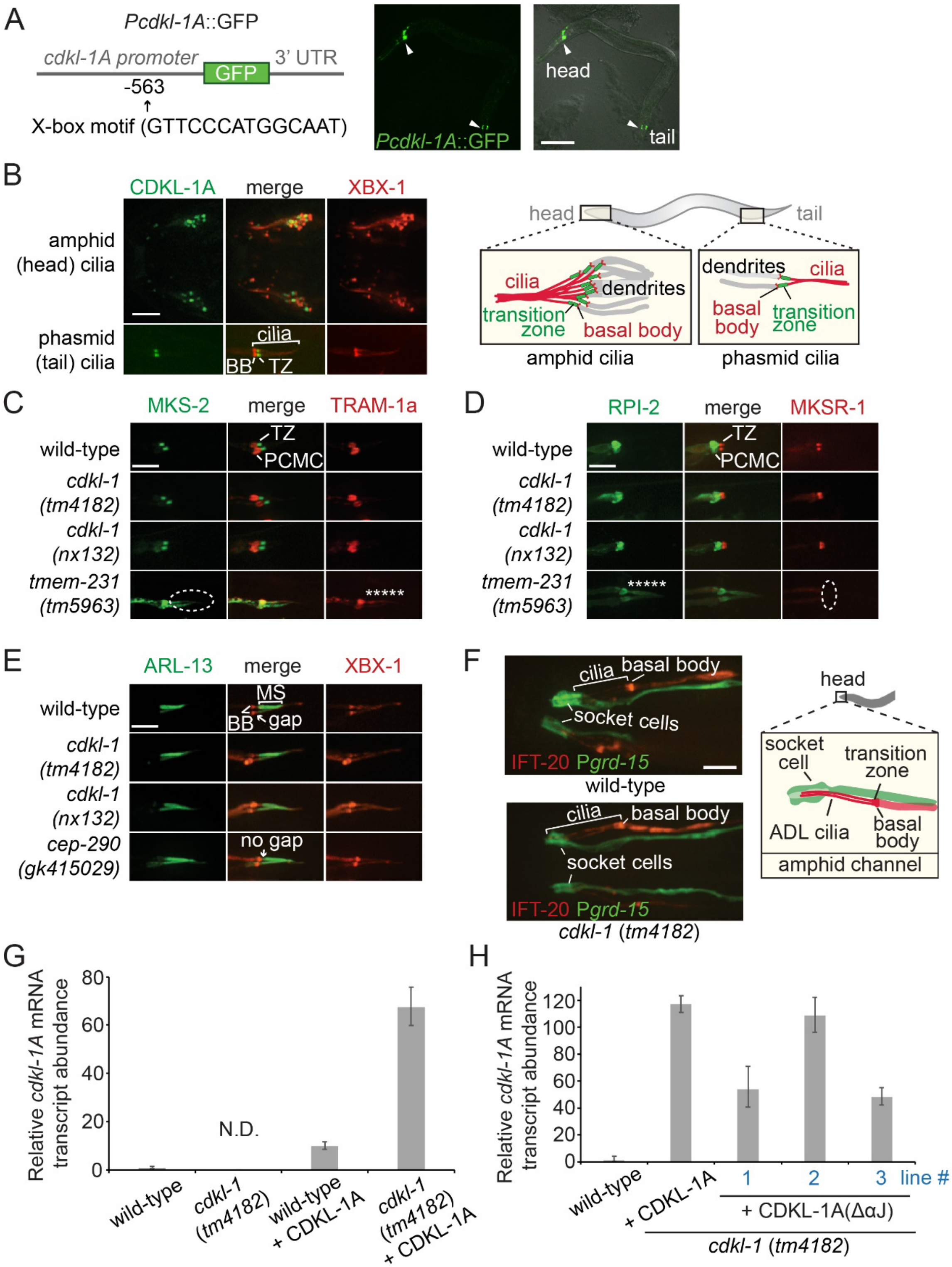

Figure S4. CDKL-1, the sole *C. elegans* CDKL family member, localizes at the transition zone but does not appear to influence cilium gate function. Related to Figure 3.

(**A**) The *C. elegans cdkl-1A* promoter-GFP transcriptional fusion shown in the schematic (and containing a ciliogenic X-box regulatory motif) is specifically expressed in head and tail ciliated sensory neurons (arrow head), as shown in the fluorescent and DIC overlay images. Scale bar, 100 μm.
(**B**) GFP-tagged CDKL-1A specifically localizes at the transition zone (TZ) in head and tail cilia; the tdTomato-XBX-1 IFT protein serves to mark the basal body (BB) and axonemes in head (amphid) and tail (phasmid) sensory neuron cilia (see schematic). Scale bar, 4 μm.
(**C-E**) Disruption of CDKL-1 does not visibly influence cilium gate function. The plasma membrane proteins TRAM-1a (**C**) and RPI-2 (**D**) concentrate just outside of cilia at the periciliary membrane compartment (PCMC) in wild-type animals and *cdkl-1* mutants, indicating the presence of a normal diffusion barrier; these proteins leak into cilia in transition zone (TZ) mutant (*tmem-231*) (asterisk) where TZ function is known to be compromised. In the *tmem-231* mutant, the TZ protein co-markers, MKS-2 and MKSR-1, mislocalize (dotted ellipse). ARL-13 (**E**) localizes correctly in the middle segment (MS) in wild-type and *cdkl-1* mutants, indicating an intact ciliary gate. However, ARL-13 leaks out of cilium in the TZ mutant known to affect gate function, *cep-290* (*gk415029*). XBX-1 marks the basal body (BB) and axoneme. PCMC, periciliary membrane compartment. Scale bar, 4 μm.
(**F**) The cilia of ADL in L3 larvae of wild-type and *cdkl-1* (*tm4182*) mutant are correctly guided into amphid channels (Inglis et al., 2007). The schematic depicts how ADL cilia (red) penetrate an amphid channel formed by the sheath and socket (green) cells. The fluorescence images from wild-type or *cdkl-1* mutant animals show a cilium marker (tdTomato-tagged IFT-20 expressed in the neuron using the P*srh-220* promoter) and socket cell marker (GFP expressed using the *grd-15* promoter). Scale bar, 4μm.
(**G-H**) Relative mRNA transcript level of *cdkl-1A* or *cdkl-1A(ΔαJ)* in indicated strains. The gene expression was normalized to a reference gene (*tba-1*). The level of *cdkl-1A* transcript in wild-type is set to 1. N.D., no detection

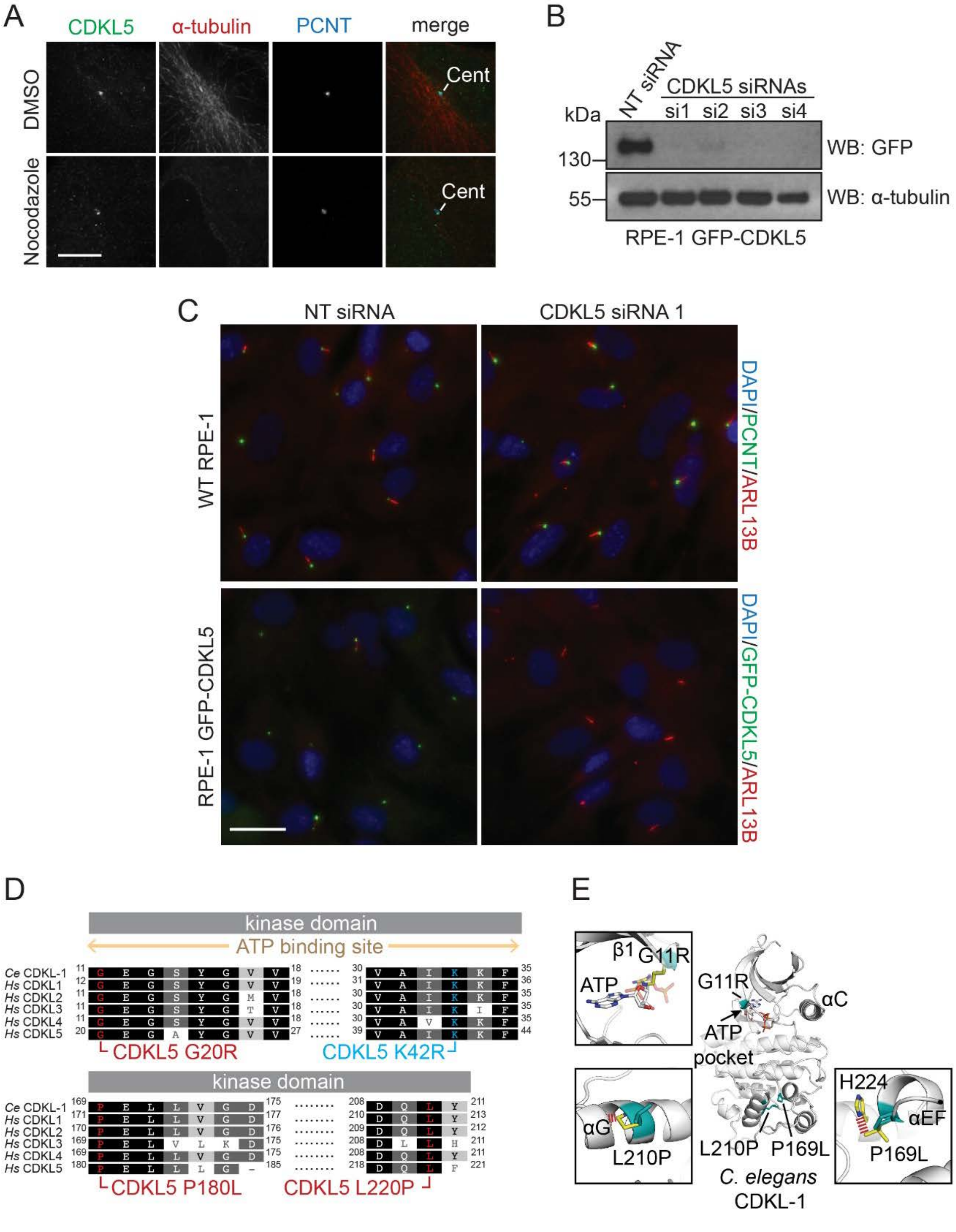

Figure S5. Negative effect on ciliogenesis of human CDKL5 overexpression in RPE-1 cells and the structural modeling of human CDKL5 pathogenic mutations in *C. elegans* CDKL-1. Related to Figure 4.
(**A**) Immunofluorescence analysis of hTERT RPE-1 GFP-CDKL5 cells were treated with DMSO or 30μM nocodazole for 1h after which they were fixed and stained with antibodies against GFP, α-tubulin and PCNT. Cent, centrosome, Scale bar, 10μm.
(**B**) Western blot analysis of GFP-CDKL5 levels in hTERT RPE-1 GFP-CDKL5 cells transfected with control non-targeting (NT) or CDKL5-directed siRNAs for 72h.

(**C**) Immunofluorescence analysis of control (NT siRNA) and CDKL5 siRNA treated hTERT RPE-1 and hTERT RPE-1 GFP-CDKL5 cells transfected with the indicated siRNAs for 72h and stained with the indicated antibodies. DNA was stained with DAPI. Scale bar, 25µm.
(**D**) Conserved CDKL5 missense mutations associated with neurological disorders (red) and catalytic lysine residue (blue) highlighted in a partial sequence alignment of human (*Hs*) and *C. elegans* (*Ce*) CDKL kinase domains.
(**E**) Locations of missense mutation residues (cyan) in a homology model of the *C. elegans* CDKL-1A structure (gray) prepared using SWISS-MODEL (Biasini et al., 2014). Inset boxes show wild-type (cyan) and mutant side chains (yellow) modelled using the DUET server (Pires et al., 2014). Steric clashes are indicated by red bars.

Table S1. Selected thermal shift screening results. Related to Figure 1 and Supplemental Experimental Procedures.

**CDKL1**			
Compound	Supplier	Supplier ID	Tm shift (°C)
ASC67	K. Shokat UCSF	ASC67	8.4
DJM2005	K. Shokat UCSF	DJM2005	7.5
K252a	Calbiochem	420298	6.9
Staurosporine	AXXORA	S-9300	6.6
Indirubin E804	Calbiochem	402081	6.1
TCS 2312	Tocris	3038	5.6
Cdk1/2 Inhibitor III	Calbiochem	217714	4.9
PI3K Inhibitor IV	Calbiochem	528111	4.7
Cdk2/9 Inhibitor	Calbiochem	238806	4.3
Curcumin	Calbiochem	239802	3.9
Indirubin-3monoxime	Calbiochem	402085	3.8
Wee1/Chk1 inhibitor	Calbiochem	681637	3.4
GSK-3 inhibitor X	Calbiochem	361551	3.2
IKK Inhibitor VII	Calbiochem	401486	3.1
JNK inhib V	Calbiochem	420129	3.1
BIM 8	AXXORA	B-7457	2.7
BIM 9	AXXORA	B-7334	2.2
VEGFR 2/3 Inhibitor	Calbiochem	676499	1.9
Quercetin	Calbiochem	551600	1.6
GSK inhibitor XIII	Calbiochem	361555	1.5
**CDKL2**			
Compound	Supplier	Supplier ID	Tm shift (°C)
TCS 2312	Tocris	3038	6.6
ASC67	K. Shokat UCSF	ASC67	5.8
K252a	Calbiochem	420298	5.6
Curcumin	Calbiochem	239802	5.5
Flt3 Inhibitor III	Calbiochem	343022	5.5
Cdk1/2 Inhibitor III	Calbiochem	217714	5.4
Dovitinib	LC Labs	D-3608	4.0
Cdk2 Inhibitor II	Calbiochem	219445	3.6
BIM 9	AXXORA	B-7334	3.6

Quercetin	Calbiochem	551600	3.4
SU9516	Calbiochem	572650	3.3
Cdk2/9 Inhibitor	Calbiochem	238806	3.1
GSK-3 Inhibitor XVI	Calbiochem	361559	3.0
SB 218078	Calbiochem	559402	2.6
Staurosporine	AXXORA	S-9300	2.5
Aurora/Cdk Inhibitor	Calbiochem	189406	2.2
Oxindole I	Calbiochem	499600	2.2
Indirubin E804	Calbiochem	402081	2.1
DJM2005	K. Shokat UCSF	DJM2005	2.1
PF 573228	Tocris	3239	2.0
**CDKL3**			
Compound	Supplier	Supplier ID	Tm shift (°C)
ASC67	K. Shokat UCSF	ASC67	7.1
K252a	Calbiochem	420298	5.4
Cdk2/9 Inhibitor	Calbiochem	238806	5.3
Quercetin	Calbiochem	551600	5.1
PI3K Inhibitor VIII	Calbiochem	528116	5.0
Curcumin	Calbiochem	239802	4.5
SU9516	Calbiochem	572650	4.2
SB 218078	Calbiochem	559402	3.6
DJM2005	K. Shokat UCSF	DJM2005	3.1
Apigenin	Calbiochem	178278	2.6
Raf1 kinase inhibitor II	Calbiochem	553011	2.4
Cdk1/2 Inhibitor III	Calbiochem	217714	2.3
Indirubin E804	Calbiochem	402081	2.2
Flt3 Inhib III	Calbiochem	343022	2.2
Staurosporine	AXXORA	S-9300	2.0
Debromohymenialdisine	Calbiochem	252010	1.8
Cdc7/CDK9 inhibitor	Calbiochem	217707	1.7
Alsterpaullone	Calbiochem	126870	1.6
Cdk2 Inhibitor II	Calbiochem	219445	1.4
TCS 2312	Tocris	3038	0.4
**CDKL5**			
Compound	Supplier	Supplier ID	Tm shift (°C)

ASC67	K. Shokat UCSF	ASC67	7.4
K252a	Calbiochem	420298	5.3
Wee1/Chk1 inhibitor	Calbiochem	681637	5.3
Indirubin E804	Calbiochem	402081	5.0
SU9516	Calbiochem	572650	4.6
BIBX1382	Calbiochem	324832	4.5
Flavopiridol	AXXORA	ALX-430	4.5
Quercetin	Calbiochem	551600	4.5
Curcumin	Calbiochem	239802	4.2
Staurosporine	AXXORA	S-9300	4.0
Apigenin	Calbiochem	178278	3.8
Cdk2/9 Inhibitor	Calbiochem	238806	3.8
Cdk1/2 Inhibitor III	Calbiochem	217714	3.3
Oxindole I	Calbiochem	499600	3.3
BIM I	Calbiochem	203291	3.3
Cdc7/CDK9 inhibitor	Calbiochem	217707	3.3
5-Iodotubercidin	Calbiochem	407900	3.2
Alsterpaullone	Calbiochem	126870	2.9
TCS 2312	Tocris	3038	0.3
DJM2005	K. Shokat UCSF	DJM2005	0.1

Table S2. Crystallographic data collection and refinement statistics. Related to Figure 1.

Protein	CDKL1	CDKL2	CDKL2	CDKL3	CDKL5
**Inhibitor**	DJM2005	Cdk1/2 Inhibitor III	TCS2312	ASC67	ASC67
**Data collection**					
**Beamline**	I24	I02	I04-1	I04	I03
**Wavelength (Å)**	0.9778	0.9795	0.9173	0.9611	0.9795
**Resolution range (Å)**	29.78 - 2.4 (2.53 - 2.4)	42.14 - 1.6 (1.69 - 1.6)	39.39 - 2.0 (2.072 - 2.0)	54.54 - 2.2 (2.27 - 2.2)	55.02 - 2.0 (2.05 - 2.0)
**Space group**	$P\ 3_2$	$P\ 2_1\ 2_1\ 2_1$	$P\ 1$	$P\ 3_1\ 2\ 1$	$P\ 2_1\ 2_1\ 2_1$
**Cell dimensions**					
**a, b, c (Å)**	124 124 49.3	67.8 70.2 83.7	31.6 68 82.3	63.9 63.9 163.6	51.9 65.5 102.9
**α, β, γ (°)**	90 90 120	90 90 90	71.5 89.9 89	90 90 120	90 90 90
**Total reflections**	157705 (22867)	733038 (60837)	148975 (11552)	106415 (9026)	293728 (15016)
**Unique reflections**	33165 (4877)	53461 (7693)	42854 (3326)	20446 (1723)	23934 (2187)
**Multiplicity**	4.8 (4.7)	13.7 (7.9)	3.5 (3.5)	5.2(5.2)	12.2 (8.9)
**Completeness (%)**	99.98 (100.00)	100.00 (100.00)	97.6 (98.4)	99.9 (100)	99.04 (92.55)
**Mean I/sigma(I)**	8.2 (2)	13.4 (2.1)	11.2 (2.4)	10.7 (2.5)	22.6 (4.8)
**Wilson B-factor**	44.82	20.24	16.14	32.45	24.76
**R-meas**	0.123 (0.777)	0.148 (1.223)	0.153 (1.017)	0.117 (0.782)	0.079 (0.414)
**CC1/2**	0.93 (0.738)	0.999 (0.645)	0.945 (0.696)	0.997 (0.906)	0.999 (0.935)
**Phasing model(s)**	4AAA	3NIZ	4AAA	4AAA, 4AGU	4BBM
**Mols ASU**	3	1	2	1	1
**Refinement**					
**R-work**	0.1865	0.1803	0.2425	0.2142	0.1738
**R-free**	0.2261	0.2082	0.269	0.2625	0.2014
**Number of atoms**	6810	2826	4956	2516	2413
**macromolecules**	6399	2515	4686	2319	2217
**ligands**	96	52	80	38	44
**water**	315	259	190	159	152
**Protein residues**	831	295	606	297	276
**RMS(bonds) (Å)**	0.013	0.014	0.009	0.013	0.009
**RMS(angles) (Å)**	1.66	1.53	1.24	1.49	1.29
**Ramachandran favored (%)**	98	98	97	97	98
**Ramachandran outliers (%)**	0	0	0	0	0

**Clashscore**	1.89	4.49	2.37	2.81	2
**Average B-factor (Å²)**	60.7	28.8	29.6	56.9	29.5
**macromolecules**	60.6	28	29.6	57.6	29
**ligands**	64.4	27.7	32.4	45.9	32.2
**solvent**	60.5	36.4	29.5	49.5	35.9
**PDB ID**	4AGU	4AAA	4BBM	3ZDU	4BGQ

Table S3. ***C. elegans*** **strains used in this study. Related to Experimental Procedures.**

Strain	Genotype
N2	*Bristol wild-type*
MX1420	*nxEx172[Pbbs-8::tram-1::tdtomato; Pbbs-8::mks-2::gfp; rol-6(su1006)]*
MX1388	*nxEx231[arl-13::gfp; Posm-5::xbx-1::tdtomato; rol-6(su1006)]*
MX1813	*tmem-231*(*tm5963*); *nxEx172[Pbbs-8::tram-1::tdtomato; Pbbs-8::mks-2::gfp; rol-6(su1006)]*
MX1924	*raIs12964[Pgrd-15::gfp]; nxEx256[Psrh-220::ift-20::tdtomato; rol-6(su1006)]*
MX1929	*nxEx969[cdkl-1A::gfp; xbx-1::tdtomato; rol-6(su1006)]*
MX1932	*nxEx250[rpi-2::gfp; mksr-1::tdtomato; rol-6(su1006)]*
MX1938	*cdkl-1(tm4182); nxEx250[rpi-2::gfp; mksr-1::tdtomato; rol-6(su1006)]*
MX1940	*tmem-231*(*tm5963*); *nxEx250[rpi-2::gfp; mksr-1::tdtomato; rol-6(su1006)]*
MX1943	*cdkl-1(tm4182); nxEx231[arl-13::gfp; Posm-5::xbx-1::tdtomato; rol-6(su1006)]*
MX1965	*cep-290*(*gk415029*); *nxEx231[arl-13::gfp; Posm-5::xbx-1::tdtomato; rol-6(su1006)]*
MX2100	*cdkl-1(tm4182); nxEx172[Pbbs-8::tram-1::tdtomato; Pbbs-8::mks-2::gfp; rol-6(su1006)]*
MX2102	*cdkl-1(tm4182)*
MX2246	*cdkl-1(nx132); nxEx172[Pbbs-8::tram-1::tdtomato; Pbbs-8::mks-2::gfp; rol-6(su1006)]*
MX2247	*cdkl-1(nx132); nxEx231[arl-13::gfp; Posm-5::xbx-1::tdtomato; rol-6(su1006)]*
MX2277	*cdkl-1(nx131) (c. G90T, A98G)(p.K33R)*
MX2278	*cdkl-1(nx132) (c. 85ATTGT89del)*
MX2293	*nxEx258[cdkl-1A(K33R)::tdtomato; Pbbs-8::mks-2::gfp; rol-6(su1006)]*
MX2301	*nxEx1193[cdkl-1A(G11R)::tdtomato; Pbbs-8::mks-2::gfp; rol-6(su1006)]*
MX2303	*nxEx1195[cdkl-1A(L210P)::tdtomato; Pbbs-8::mks-2::gfp; rol-6(su1006)]*
MX2304	*nxEx1329[cdkl-1A::tdtomato; Pbbs-8::mks-2::gfp; rol-6(su1006)]*
MX2362	*nxEx1232[cdkl-1A(P169L)::tdtomato; Pbbs-8::mks-2::gfp; rol-6(su1006)]*
MX2397	*cdkl-1(nx132); nxEx250[rpi-2::gfp; mksr-1::tdtomato; rol-6(su1006)]*
MX2415	*cdkl-1(tm4182); raIs12964[Pgrd-15::gfp]; nxEx256[Psrh-220::ift-20::tdtomato; rol-6(su1006)]*
MX2426	*nxIs30[Psrh-220::IFT-20::gfp; cc::gfp]*
MX2427	*cdkl-1(nx132); nxIs30[Psrh-220::ift-20::gfp; cc::gfp]*
MX2428	*cdkl-1(nx131); nxIs30[Psrh-220::ift-20::gfp; cc::gfp]*
MX2429	*cdkl-1(tm4182); nxIs30[Psrh-220::ift-20::gfp; cc::gfp]*
MX2447	*cdkl-1(tm4182); nxIs30[Psrh-220::ift-20::gfp; cc::gfp]; nxEx261[cdkl-1A; coel::rfp]*
MX2471	*nxEx1282[Pcdkl-1A::gfp; rol-6(su1006)]*
MX2527	*nxIs30[Psrh-220::ift-20::gfp; cc::gfp]; nxEx261[cdkl-1A; coel::rfp]*
MX2538	*cdkl-1(tm4182); nxIs30[Psrh-220::ift-20::gfp; cc::gfp]; nxEx1344[cdkl-1A(G11R); coel::rfp] line1*
MX2539	*cdkl-1(tm4182); nxIs30[Psrh-220::ift-20::gfp; cc::gfp]; nxEx1345[cdkl-1A(G11R); coel::rfp] line2*
MX2540	*cdkl-1(tm4182); nxIs30[Psrh-220::ift-20::gfp; cc::gfp]; nxEx1346[cdkl-1A(P169L); coel::rfp] line1*
MX2541	*cdkl-1(tm4182); nxIs30[Psrh-220::ift-20::gfp; cc::gfp]; nxEx1347[cdkl-1A(L210P); coel::rfp] line1*
MX2542	*cdkl-1(tm4182); nxIs30[Psrh-220::ift-20::gfp; cc::gfp]; nxEx1348[cdkl-1A(L210P); coel::rfp] line2*
MX2544	*cdkl-1(tm4182); nxIs30[Psrh-220::ift-20::gfp; cc::gfp]; nxEx2544[cdkl-1A(P169L); coel::rfp] line3*
MX2546	*cdkl-1(tm4182); nxIs30[Psrh-220::ift-20::gfp; cc::gfp]; nxEx2546[cdkl-1A(P169L); coel::rfp] line2*
MX2560	*cdkl-1(tm4182); nxIs30[Psrh-220::ift-20::gfp; cc::gfp]; nxEx2560[cdkl-1A; coel::rfp] line1*
MX2561	*cdkl-1(tm4182); nxIs30[Psrh-220::ift-20::gfp; cc::gfp]; nxEx2561[cdkl-1A; coel::rfp] line2*
MX2642	*cdkl-1(tm4182); nxIs30[Psrh-220::ift-20::gfp; cc::gfp]; nxEx2642[cdkl-1A; coel::rfp] line3*

MX2761	*nxEx2761[cdkl-1A(ΔαJ)::mNeonGreen; Posm-5::xbx-1::tdTomato; rol-6(su1006)]*
MX2764	*cdkl-1(tm4182); nxIs30[Psrh-220::ift-20::gfp; cc::gfp]; nxEx2764[cdkl-1A(ΔαJ); coel::rfp] line1*
MX2765	*cdkl-1(tm4182); nxIs30[Psrh-220::ift-20::gfp; cc::gfp]; nxEx2765[cdkl-1A(ΔαJ); coel::rfp] line2*
MX2766	*cdkl-1(tm4182); nxIs30[Psrh-220::ift-20::gfp; cc::gfp]; nxEx2766[cdkl-1A(ΔαJ); coel::rfp] line3*

SUPPLEMENTAL EXPERIMENTAL PROCEDURES

Cloning of human CDKL kinase domains

Bacmids were prepared in *E. coli* strain DH10Bac and used to generate baculoviruses in Sf9 insect cells. For activity assays, wild-type sequences (residues corresponding to CDKL1, 1-357; CDKL1(ΔαJ), 1-287; CDKL2, 1-312; CDKL2(ΔαJ), 1-287; CDKL3, 1-313; CDKL3(ΔαJ), 1-286; CDKL5, 1-831; CDKL5(ΔαJ), 1-299) were cloned into a 2 μm P_{GAL1}-kinase-TAP plasmid (pRSAB1234 backbone, originally a gift from Erin O'Shea) for expression in *S. cerevisiae.*

Expression and purification of human CDKL kinase domains

For structural studies, CDKL1 was expressed in *E. coli* strain BL21(DE3)-R3-pRARE using 0.4 mM isopropyl 1-thio-β-D-galactopyranoside for overnight induction at 18°C. Harvested cells were resuspended in binding buffer (50 mM HEPES pH 7.5, 500 mM NaCl, 5% glycerol, 5 mM Imidazole) supplemented with protease inhibitor cocktail set V (Calbiochem) at 1:1000 dilution and 1 mM tris(2-carboxyethyl)-phosphine (TCEP). To express CDKL2, CDKL3 and CDKL5, baculoviruses were used to infect Sf9 cells grown in suspension at a density of $2x10^6$ cells/mL in Insect-Xpress media (Lonza). Cells were incubated at 27°C and harvested 72 hr post-infection. Harvested cells were resuspended in binding buffer supplemented similarly with protease inhibitors and 1 mM TCEP. All cells were disrupted by sonication or high-pressure homogenization. Polyethylenimine (PEI) was added to a final concentration of 0.5% to precipitate DNA and the cell lysate clarified by centrifugation at 21,000 RPM at 4°C for 1 hr. Proteins were purified by nickel-affinity and size exclusion chromatography. Tobacco etch virus protease A (TEV) was used to proteolytically cleave the polyhistidine tag overnight at 4°C from all proteins except CDKL2.

For activity assays, W303 *S. cerevisiae* strains were grown overnight to log phase in SC-URA media containing 2% raffinose (Sigma), and then expression of N-terminal kinase domains was induced by addition of 2% galactose (Sigma) for 4 hr at 30°C. Cells were harvested by centrifugation at 8000 RPM, cell pellet washed and resuspended in 1x cell volume of lysis buffer containing 25 mM HEPES pH 8.0, 300 mM NaCl, 0.1% NP-40, 30 mM EGTA, 1 mM EDTA, and a protease/phosphatase inhibitor set was added immediately prior to harvest including 80 mM β-glycerophosphate, 50 mM NaF, 1 mM DTT, 1 mM Na_3O_4V, and 1 mM PMSF. The cell slurry was slowly dripped into liquid nitrogen to produce frozen pellets. These pellets were then pulverized in a cryogenic ball mill (Retsch MM301 with 50 ml stainless steel grinding jars) by five rounds of agitation at 15 Hz for 2 min, re-cooling the grinding jars in liquid N_2 after each cycle. The grindate was then thawed and cell debris was cleared by centrifugation at 8000 rpm for 30 min followed by sequential filtration through 2.7 and 1.6 μm Whatman GD/X filters (GE). C-terminally TAP-tagged kinases were immobilized on IgG Sepharose 6 Fast Flow beads (GE). These beads (~500 μL slurry per 1 L culture) had been pre-equilibrated in lysis buffer with inhibitors and were then incubated with lysate for 1 hr at 4°C. Bound beads were then loaded into a disposable Bio-Spin column (cat. #732-6008; BioRad) by pipette and washed with 20 mL total wash buffer (lysis buffer + 10% glycerol, 1 mM DTT) at 4°C. The column was then rotated for 20 min at 23°C in 700 μL wash buffer as a final wash to mimic elution conditions. The bound protein was then cleaved from the IgG beads by TEV protease in 600 μL elution buffer (0.21 mg/mL TEV protease [QB3 MacroLab, UC Berkeley], 25 mM HEPES pH 8.0, 310 mM NaCl, 0.09% NP-40, 26.9 mM EGTA, 0.9 mM EDTA, 1 mM DTT, and 10% glycerol) for 1 hr at 23°C.

Thermal melting shift assay

Proteins were buffered in 10 mM HEPES pH 7.5, 500 mM NaCl, 5% glycerol and a 1:1000 dilution of SYPRO Orange fluorescent dye (Invitrogen, CA). Proteins at 2 μM were mixed with inhibitor compounds at 10 μM and thermal melting shift (Tm Shift) assays run using a Mx3005p real-time PCR machine (Agilent) as described (Niesen et al., 2007).

Structure determination

Crystals were grown using the sitting-drop vapor-diffusion technique and cryo-protected by addition of 25% ethylene glycol before vitrification in liquid nitrogen. Crystallization conditions and co-crystallized inhibitors are listed in Table EV5. Diffraction data were collected at 100K using beam lines at Diamond Light Source, UK. Data were indexed and integrated using XDS (Kabsch, 2010) or MOSFLM (Leslie and Powell, 2007) and scaled using SCALA (Evans, 2006; Evans, 2011) or AIMLESS (Evans and Murshudov, 2013) in the CCP4 suite of programs (Winn et al., 2011). Phases were found using molecular replacement in PHASER (McCoy et al., 2007). SCULPTOR was used to optimize PDB entries for use as search models. The structures were built using PHENIX. AUTOBUILD

(Adams et al., 2010) and then refined and modified using alternate rounds of REFMAC5 (Murshudov et al., 2011) or BUSTER (Bricogne et al., 2011) and COOT (Emsley and Cowtan, 2004; Emsley et al., 2010). TLS groups were determined using the TLSMD server (Painter and Merritt, 2006). The refined structures were validated with MolProbity (Chen et al., 2010) and the atomic coordinate files deposited in the Protein Data Bank (PDB) with Autodep (Yang et al., 2004). Structure figures were prepared with PyMOL (The PyMOL Molecular Graphics System, Version 1.2r3pre, Schrödinger, LLC) and electrostatic surface maps calculated using DelPhi (Li et al., 2012). Sequence alignments were prepared with Clustal Omega (Sievers and Higgins, 2014) and ESPript (http://espript.ibcp.fr) (Robert and Gouet, 2014).

In vitro kinase assays
Peptide substrate (acetyl-RPRSPGARR-amide) was obtained from Tufts University Core Facility and added to a final concentration of 200 μM to start the reaction, or at variable concentration for Michaelis–Menten curves. Reactions were aliquoted onto Whatman P81 phosphocellulose (GE) strips, which were then quenched and washed 5 x in 75 mM phosphoric acid to remove free [γ-^{32}P] ATP. Samples were dried on a slab gel dryer (Model 1125B; BIORAD) and exposed to a phosphor screen (Molecular Dynamics) to determine the rate of [γ-^{32}P] ATP incorporation. Phosphor screens were analyzed with a Typhoon 9400 scanner (Amersham) using ImageQuant software (GE). Final Image quantification was performed using ImageJ (http://imagej.nih.gov/ij/). Data were fit by nonlinear regression to the Michaelis–Menten model $V_0 = V_{max}*[S]/K_M+[S]$ using Prism (GraphPad software) and Matlab (MathWorks).

C. elegans mutant and transgenic strains
C. elegans cep-290 (*gk415029*), *tmem-231* (*tm5963*) and *cdkl-1 (tm4182)* mutant strains were obtained from the *C. elegans* Gene Knockout Consortium or National BioResource Project. *cdkl-1* kinase-dead (*nx131*, p. K33R) and *cdkl-1* null (*nx132,* c. 85-89 deletion) mutants were generated with the CRISPR/CAS9 system (Friedland et al., 2013; Kim et al., 2014). All mutant strains were outcrossed to N2 (wild-type) strain at least five times and genotyped with single-worm PCR. The *cdkl-1* CRISPR guide RNA sequence (5’-AGGGATACTGGACAAATTG-3’) was predicted by the CRISPR Design Tool (http://crispr.mit.edu/) and was substituted for *unc-119* sequence in pU6::unc-119 small-guide RNA (sgRNA) vector (Addgene #46169) (Friedland et al., 2013) by site-directed mutagenesis using overlap extension PCR (Zoller, 1991). The *nx131* allele was made by CRISPR-mediated homologous recombination. 2Kb homologous donor DNA template of *cdkl-1A* harboring two mutations, V30V (silence mutation in PAM site) and K33R (kinase-dead), was amplified by stitch-PCR (Reikofski and Tao, 1992) and cloned into a pJET1.2 vector. 50ng/μl each of *cdkl-1* sgRNA vector, Cas9 plasmid (Addgene #46168), pRF4::*rol-6(su1006)* and with or without the *cdkl-1* donor plasmid were mixed and injected into 20 young adult worms. F1 heterozygous (roller) and F2 homozygous (non-roller) worms of either the *nx131* or the *nx132* mutant were identified by tetra-primer ARMS-PCR (Ye et al., 2001) and confirmed by DNA sequencing.

Transgenic animals carrying separate extrachromosomal DNA arrays with different mutant backgrounds were created by worm microinjection method and classical genetic methods. The extrachromosomal array [*Psrh220::ift-20::gfp*; *cc::gfp*] was integrated into the chromosome with 1.5 krads of X-ray irradiation for 135 seconds at 145kilovolts/5milliamps (TORREX150D X-Ray Inspection System) for ADL ciliary length measurement and analysis (McKay et al., 2003). The X-ray integrated strains were outcrossed to wild-type for six times to remove X-ray-induced mutations.

Lentiviral production and generation of the hTERT RPE-1 GFP-CDKL5 stable cell line
For the production of lentiviral particles, HEK293T cells were co-transfected with pHR-SIN-SFFV-GFP-CDKL5 and the second generation packaging (pCMV-dR8.74psPAX2) and envelope (pMD2.G) plasmids using the Lipofectamine® 3000 transfection reagent (Invitrogen) according to the manufacturer’s instructions. Lentiviral particles in conditioned media from HEK 293T cells, collected at 48h post transfection, were used to transduce hTERT RPE-1 cells. GFP positive cells were cell sorted to establish the final hTERT RPE-1 GFP-CDKL5 cell line.

Human cell lines and growth conditions
HEK 293T cells were grown in Dulbecco's modified Eagle's medium (DMEM) supplemented with 10% fetal bovine serum (FBS) and GlutaMAX™. hTERT RPE-1 and hTERT RPE-1 GFP-CDKL5 were grown in DMEM/F12 medium supplemented with 10% FBS. To induce ciliogenesis, hTERT RPE-1 and hTERT RPE-1 GFP-CDKL5 cells were cultured in DMEM/F12 medium without FBS. All cells were cultured in a 5% CO_2 humidified atmosphere at 37°C.

Western blot analyses
For western blot analysis, the cells were lysed in Laemmli buffer and treated with benzonase nuclease (Sigma-Aldrich). Whole cell lysates were incubated at 95ºC for 5 min and loaded onto a 10% SDS-PAGE gel. The proteins were separated by electrophoresis and then transferred to a PVDF membrane (Immobilon-P, Millipore). Membranes were incubated with primary antibodies against GFP (Roche, 11 814 460 001) and α-tubulin (Sigma-Aldrich, T6199) in TBST (TBS, 0.1% Tween-20) in 5% skim milk powder (Bioshop). Blots were washed 3x 10 min in TBST, and then incubated with secondary HRP-conjugated antibodies. After being washed again in TBST, the western blots were developed using SuperSignal reagents from Thermo Scientific.

Nocodazole treatment
hTERT RPE-1 GFP-CDKL5 cells were incubated in the presence of DMSO (control) or 30µM nocodazole for 1 h to promote the depolymerization of microtubules. After the treatment, the cells were washed with PBS and processed for immunofluorescence analysis.

Immunofluorescence microscopy
For immunofluorescence, the cells were fixed with ice-cold methanol (10 min at -20°C), blocked with 0.2% Fish Skin Gelatin (Sigma-Aldrich) in 1x PBS (20 min), incubated with the primary antibodies in blocking solution (1h), washed with blocking solution and incubated with fluorophore-conjugated secondary antibodies and DAPI (0.1µg/ml) in blocking solution (1h). After a final wash in blocking solution the coverslips were mounted on glass slides by inverting them onto mounting solution (ProLong Gold antifade, Molecular Probes). The primary antibodies used were: anti-GFP (Roche, 11 814 460 001), anti-ARL13B (Proteintech, 17711-1), anti-PCNT (Santa Cruz Biotechnology, sc-28143), anti-IFT88(Proteintech, 13967-1-AP), anti-polyglutamylated tubulin (GT335, AdipoGen AG-20B-0020), and α-tubulin (Sigma-Aldrich, T6199). The secondary antibodies used were: Alexa488/594/647 anti-mouse, anti-rabbit and anti-goat antibodies from Life Technologies. The cells were imaged either with a 20 x air objective (0.75 NA; **Figure S5C**) or a 60 x oil-immersion objective (1.42 NA; **Figures 4A,B** and **S5A**) on a Deltavision Elite DV imaging system equipped with a sCMOS 2048x2048 pixels2 camera (GE Healthcare). When the 60 x objective was used, z stacks (0.2 µm apart) were collected, deconvolved using softWoRx (v5.0, Applied Precision) and are shown as maximum intensity projections (pixel size 0.1064 µm).

Phylogenetic analysis
The phylogram showing human and *C. elegans* CDKL protein evolutionary relationships was generated using PHYML (bootstrap 1,000) at http://www.atgc-montpellier.fr/phyml and www.phylogeny.fr (Altschul et al., 1997; Chevenet et al., 2006; Dereeper et al., 2010; Dereeper et al., 2008; Edgar, 2004; Guindon et al., 2010). Branch support values (%) are displayed. Protein sequences: *Hs* CDKL1 (Uniprot Q00532), *Hs* CDKL2 (Q92772), *Hs* CDKL3 (Q8IVW4), *Hs* CDKL4 (Q5MAI5), *Hs* CDKL5 (O76039) and *Ce* CDKL-1 (Q9U2H1).

Quantitation of cdkl-1A transcript
cdkl-1A transcript levels were measured using quantitative real-time PCR. cDNA was prepared using Invitrogen SuperScript III reverse transcriptase by following manufacturer's protocol. The RT-PCR was performed using SsoAdvanced Universal SYBR Green Supermix (Biorad) and the StepOne Real-Time PCR System. The relative levels of *cdkl-1A* mRNA transcripts were compared to wild-type which was set to 1. Primers: tba-1 forward (tcaacactgccatcgccgcc) and reverse (tccaagcgagaccaggcttcag), cdkl-1 forward (agatttcggatttgctcgaa) and reverse (gcatatacacatcctacagccc).

SUPPLEMENTAL REFERENCES

Adams, P.D., Afonine, P.V., Bunkoczi, G., Chen, V.B., Davis, I.W., Echols, N., Headd, J.J., Hung, L.W., Kapral, G.J., Grosse-Kunstleve, R.W., et al. (2010). PHENIX: a comprehensive Python-based system for macromolecular structure solution. Acta Crystallogr. D Biol. Crystallogr. *66*, 213-221.

Altschul, S.F., Madden, T.L., Schaffer, A.A., Zhang, J., Zhang, Z., Miller, W., and Lipman, D.J. (1997). Gapped BLAST and PSI-BLAST: a new generation of protein database search programs. Nucleic Acids Res. *25*, 3389-3402.

Biasini, M., Bienert, S., Waterhouse, A., Arnold, K., Studer, G., Schmidt, T., Kiefer, F., Gallo Cassarino, T., Bertoni, M., Bordoli, L., et al. (2014). SWISS-MODEL: modelling protein tertiary and quaternary structure using evolutionary information. Nucleic Acids Res. *42*, W252-258.

Brenner, S. (1974). The genetics of Caenorhabditis elegans. Genetics *77*, 71-94.

Bricogne, G., Blanc, E., Brandl, M., Flensburg, C., Keller, P., Paciorek, W., Roversi, P., Sharff, A., Smart, O.S., Vonrhein, C., et al. (2011). BUSTER, Version 2.10.0 Ed. Global Phasing Ltd., Cambridge, UK.

Chen, V.B., Arendall, W.B., 3rd, Headd, J.J., Keedy, D.A., Immormino, R.M., Kapral, G.J., Murray, L.W., Richardson, J.S., and Richardson, D.C. (2010). MolProbity: all-atom structure validation for macromolecular crystallography. Acta Crystallogr. D Biol. Crystallogr. *66*, 12-21.

Chevenet, F., Brun, C., Banuls, A.L., Jacq, B., and Christen, R. (2006). TreeDyn: towards dynamic graphics and annotations for analyses of trees. BMC Bioinformatics *7*, 439.

Dereeper, A., Audic, S., Claverie, J.M., and Blanc, G. (2010). BLAST-EXPLORER helps you building datasets for phylogenetic analysis. BMC Evol. Biol. *10*, 8.

Dereeper, A., Guignon, V., Blanc, G., Audic, S., Buffet, S., Chevenet, F., Dufayard, J.F., Guindon, S., Lefort, V., Lescot, M., et al. (2008). Phylogeny.fr: robust phylogenetic analysis for the non-specialist. Nucleic Acids Res. *36*, W465-469.

Edgar, R.C. (2004). MUSCLE: multiple sequence alignment with high accuracy and high throughput. Nucleic Acids Res. *32*, 1792-1797.

Emsley, P., and Cowtan, K. (2004). Coot: model-building tools for molecular graphics. Acta Crystallogr. D Biol. Crystallogr. *60*, 2126-2132.

Emsley, P., Lohkamp, B., Scott, W.G., and Cowtan, K. (2010). Features and development of Coot. Acta Crystallogr. D Biol. Crystallogr. *66*, 486-501.

Evans, P. (2006). Scaling and assessment of data quality. Acta Crystallogr. D Biol. Crystallogr. *62*, 72-82.

Evans, P.R. (2011). An introduction to data reduction: space-group determination, scaling and intensity statistics. Acta Crystallogr. D Biol. Crystallogr. *67*, 282-292.

Evans, P.R., and Murshudov, G.N. (2013). How good are my data and what is the resolution? Acta Crystallogr. D Biol. Crystallogr. *69*, 1204-1214.

Friedland, A.E., Tzur, Y.B., Esvelt, K.M., Colaiacovo, M.P., Church, G.M., and Calarco, J.A. (2013). Heritable genome editing in C. elegans via a CRISPR-Cas9 system. Nat Methods *10*, 741-743.

Guindon, S., Dufayard, J.F., Lefort, V., Anisimova, M., Hordijk, W., and Gascuel, O. (2010). New algorithms and methods to estimate maximum-likelihood phylogenies: assessing the performance of PhyML 3.0. Syst. Biol. *59*, 307-321.

Inglis, P.N., Ou, G., Leroux, M.R., and Scholey, J.M. (2007). The sensory cilia of Caenorhabditis elegans. WormBook, 1-22.

Kabsch, W. (2010). Xds. Acta Crystallogr. D Biol. Crystallogr. *66*, 125-132.

Kim, H., Ishidate, T., Ghanta, K.S., Seth, M., Conte, D., Jr., Shirayama, M., and Mello, C.C. (2014). A co-CRISPR strategy for efficient genome editing in Caenorhabditis elegans. Genetics *197*, 1069-1080.

Leslie, A.G.W., and Powell, H.R. (2007). Processing diffraction data with MOSFLM. (Netherlands: Springer).

Li, L., Li, C., Sarkar, S., Zhang, J., Witham, S., Zhang, Z., Wang, L., Smith, N., Petukh, M., and Alexov, E. (2012). DelPhi: a comprehensive suite for DelPhi software and associated resources. BMC Biophys. *5*, 9.

McCoy, A.J., Grosse-Kunstleve, R.W., Adams, P.D., Winn, M.D., Storoni, L.C., and Read, R.J. (2007). Phaser crystallographic software. J Appl Crystallogr *40*, 658-674.

McKay, S.J., Johnsen, R., Khattra, J., Asano, J., Baillie, D.L., Chan, S., Dube, N., Fang, L., Goszczynski, B., Ha, E., et al. (2003). Gene expression profiling of cells, tissues, and developmental stages of the nematode C. elegans. Cold Spring Harb. Symp. Quant. Biol. *68*, 159-169.

Murshudov, G.N., Skubak, P., Lebedev, A.A., Pannu, N.S., Steiner, R.A., Nicholls, R.A., Winn, M.D., Long, F., and Vagin, A.A. (2011). REFMAC5 for the refinement of macromolecular crystal structures. Acta Crystallogr. D Biol. Crystallogr. *67*, 355-367.

Niesen, F.H., Berglund, H., and Vedadi, M. (2007). The use of differential scanning fluorimetry to detect ligand interactions that promote protein stability. Nat. Protoc. *2*, 2212-2221.

Painter, J., and Merritt, E.A. (2006). Optimal description of a protein structure in terms of multiple groups undergoing TLS motion. Acta Crystallogr. D Biol. Crystallogr. *62*, 439-450.

Pires, D.E., Ascher, D.B., and Blundell, T.L. (2014). DUET: a server for predicting effects of mutations on protein stability using an integrated computational approach. Nucleic Acids Res. *42*, W314-319.

Reikofski, J., and Tao, B.Y. (1992). Polymerase chain reaction (PCR) techniques for site-directed mutagenesis. Biotechnol Adv *10*, 535-547.

Robert, X., and Gouet, P. (2014). Deciphering key features in protein structures with the new ENDscript server. Nucleic Acids Res. *42*, W320-324.

Sievers, F., and Higgins, D.G. (2014). Clustal Omega, accurate alignment of very large numbers of sequences. Methods Mol. Biol. *1079*, 105-116.

Winn, M.D., Ballard, C.C., Cowtan, K.D., Dodson, E.J., Emsley, P., Evans, P.R., Keegan, R.M., Krissinel, E.B., Leslie, A.G., McCoy, A., et al. (2011). Overview of the CCP4 suite and current developments. Acta Crystallogr. D Biol. Crystallogr. *67*, 235-242.

Yang, H., Guranovic, V., Dutta, S., Feng, Z., Berman, H.M., and Westbrook, J.D. (2004). Automated and accurate deposition of structures solved by X-ray diffraction to the Protein Data Bank. Acta Crystallogr. D Biol. Crystallogr. *60*, 1833-1839.

Ye, S., Dhillon, S., Ke, X., Collins, A.R., and Day, I.N. (2001). An efficient procedure for genotyping single nucleotide polymorphisms. Nucleic Acids Res. *29*, E88-88.

Zoller, M.J. (1991). New molecular biology methods for protein engineering. Curr. Opin. Biotechnol. *2*, 526-531.
